# MNMST: topology of cell networks leverages identification of spatial domains from spatial transcriptomics data

**DOI:** 10.1186/s13059-024-03272-0

**Published:** 2024-05-23

**Authors:** Yu Wang, Zaiyi Liu, Xiaoke Ma

**Affiliations:** 1 School of Computer Science and Technology, Xidian University, No.2 South Taibai Road, Xi’an, 710071 Shaanxi China; 2 Key Laboratory of Smart Human-Computer Interaction and Wearable Technology of Shaanxi Province, Xidian University, No.2 South Taibai Road, Xi’an, 710071 Shaanxi China; 3 Department of Radiology, Guangdong Provincial People’s Hospital (Guangdong Academy of Medical Sciences), Southern Medical University, 106 Zhongshan Er Road, Guangzhou, 510080 Guangdong China; 4 Guangdong Provincial Key Laboratory of Artificial Intelligence in Medical Image Analysis and Application, Guangdong Provincial People’s Hospital, Guangdong Academy of Medical Sciences, 106 Zhongshan Er Road, Guangzhou, 510080 Guangdong China

**Keywords:** Spatial transcriptomics, Spatial domain, Network model, Joint learning, Topological structure, Integrative analysis

## Abstract

Advances in spatial transcriptomics provide an unprecedented opportunity to reveal the structure and function of biology systems. However, current algorithms fail to address the heterogeneity and interpretability of spatial transcriptomics data. Here, we present a multi-layer network model for identifying spatial domains in spatial transcriptomics data with joint learning. We demonstrate that spatial domains can be precisely characterized and discriminated by the topological structure of cell networks, facilitating identification and interpretability of spatial domains, which outperforms state-of-the-art baselines. Furthermore, we prove that network model offers an effective and efficient strategy for integrative analysis of spatial transcriptomics data from various platforms.

**Supplementary Information:**

The online version contains supplementary material available at 10.1186/s13059-024-03272-0.

## Background

Complex tissues are typically composed of many cell types (sub-populations) located at various positions to execute the corresponding biological functions [1–3]. Thus, it is of great significance to simultaneously exploit the expression and spatial information of cells, which is the foundation for understanding the underlying mechanisms of biology systems [4]. For example, the combination of expression and spatial information of cells provides an effective strategy to precisely characterize the heterogeneity of tumor micro-environment, which is critical for diagnosis and therapy of cancers [5]. Fortunately, advances in biological techniques ensure the generation of expression profiles of cells while retaining spatial relative context in situ, known as spatial transcriptomics, which presents unprecedented opportunities to explore the organization and function of tissues in a spatial context. On the basis of principles of preserving spatial information, current technologies are broadly classified into two categories, i.e., imaging- and next-generation sequencing (NGS)-based methods [6]. The typical imaging-based approaches include fluorescence in situ hybridization (FISH) [7] and its variants [8–10], which are criticized for their limited capacity to detect RNA transcripts. To address this issue, NGS-based methods employ spatial barcoding and NGS, including Slide-seq [11], Stereo-seq [12], and 10 $\times$ Visium [13]. The accumulated spatial transcriptomics data provide a great opportunity to investigate the functions and cellular structure of tissues by exploiting interesting patterns and features that cannot be identified from other data.

Spatial domains, that are regions where cells inside are coherent with similar expression profiles and spatial proximity, are one of the typical patterns in spatial transcriptomics data. They are the prerequisites for downstream analysis, such as tracking disease progression [14] and tissue development [8]. According to strategies of dissection, available methods for the identification of spatial domains are divided into two categories, i.e., biological experiments and computational approaches. Specifically, former methods employ anatomy to obtain the spatial information of cells and manually select spatial domains. This approach is precise and reliable given that expertise knowledge is seamlessly incorporated. However, these methods are criticized for efficiency because manual selection mode poses a great challenge in terms of time and finance. Computational methods, which identify spatial domains with machine learning, provide an alternative to overcome limitations of biological experiment-based methods.

Specifically, unsupervised clustering is a widely adopted strategy for identifying spatial domains in spatial transcriptomics data. Current computational methods for spatial domain identification are roughly categorized into two classes, i.e., non-spatial and spatial clustering algorithms. Non-spatial clustering methods, such as DRjCC [15], SCANPY [16], K-means, and Louvain [17], are deliberately designed for transcriptomics data without spatial information. These methods are criticized for their undesirable performance because identified spatial domains deviate from tissue sections, highlighting the importance of spatial information for the analysis of spatial transcriptomics data.

However, spatial clustering integrates gene expression and spatial location to address the aforementioned issues. This immediate purpose is to account for spatial dependency of gene expression, which results in better matching spatial location. In comparison to non-spatial clustering, spatial clustering significantly enhances the performance of algorithms, which indicates that spatial information plays an indispensable role in the identification of spatial domains. The greatest difference among these algorithms lies in how to obtain and integrate spatial and transcriptional features of cells. For example, BayesSpace [18] and SpatialPCA [19] employ Hidden-Markov random field to enforce physically proximal cells belonging to the same domains by assigning higher probability to proximal cells. However, these algorithms achieve an excellent performance if and only if cell types are well separated, that is the division boundary is very clear. Actually, the boundaries of spatial domains are unclear because of the hierarchical structure of cell types.

To exploit the latent structure of cells, SpaGCN [20] and STAGATE [21] adopt graph neural networks to learn the topological structure of cells, which can be concatenated with transcriptional features to facilitate identification of spatial domains. These algorithms differ greatly in strategies used to learn graph features of cells for characterizing spatial information. For example, SEDR [22] employs auto-encoder to learn spatial embedding, whereas CCST [23] addresses complex global cell interactions across tissues. BANKSY [24] simultaneously considers transcriptome of cells and their local neighbors, and DeepST [25] integrates transcriptomics and morphological features of cells with data augmentation, which provides a compatible strategy for spatial domain. SPARK-X [26] and SpatialDE [27] detect spatial variable genes to link spatial domains with biological functions for further integrating knowledge from multiple resources.

Although great efforts have been devoted to identifying spatial domains, many urgent but unaddressed problems exist. Current algorithms directly integrate the spatial and expression data. However, they are criticized for failing to remove heterogeneity of spatial transcriptomics data, which results in an undesirable performance. Second, the transcriptional and spatial features of cells are independently learned, where the relations among spatial and expression information are neglected, which leads to a failure to precisely model spatial domains. Third, available algorithms are devoted to characterize and identify spatial domains with deep features of cells, reducing interpretability of cell features. Finally, vast majority of algorithms are designed for specific platforms, which hinders the application and transplanting to others. Thus, precisely modeling and identifying spatial domains from spatial transcriptomics data is still challenging.

**Fig. 1:**
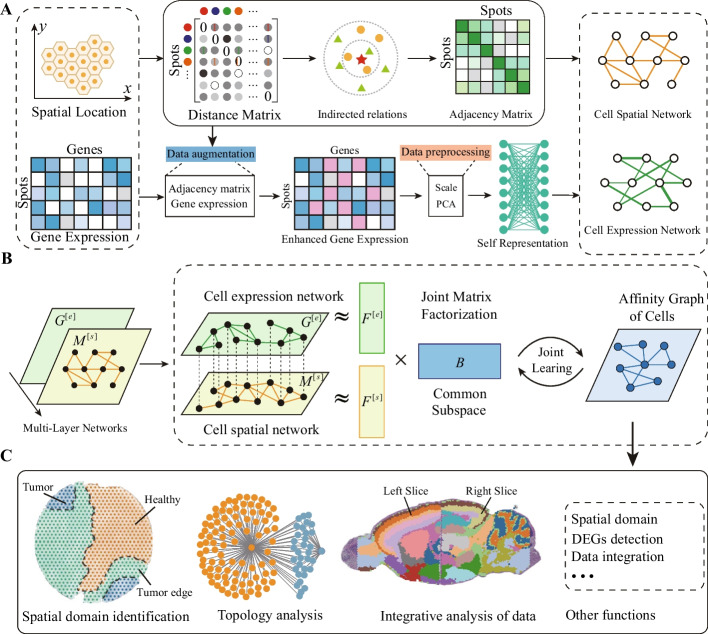
Overview of multi-layer network model (MNMST) for spatial domain identification. (A) Cell multi-layer network construction. MNMST constructs cell spatial network by exploiting indirect relations among cells and learns cell expression network by using self-representation learning (SRL) with local preservation constraint. (B) Spatial and expression feature learning. MNMST jointly factorizes cell multi-layer networks with non-negative matrix factorization by projecting cells into a common subspace to learn compatible features of cells. It automatically learns cell expression networks by utilizing SRL with local preservation constraint by exploiting augmented expression profiles of cells. (C) Down-stream analysis of affinity graph of cells, including spatial domain identification, topology analysis, and integrative analysis of spatial transcriptomics data

We propose a multi-layer network model for spatial transcriptomics data (MNMST) to accurately identify spatial domains with joint learning for addressing the aforementioned issues. As shown in Fig. 1, MNMST consists of three major components: multi-layer network construction, spatial and expression feature learning, and spatial domain identification. Specifically, MNMST first constructs a network for spatial and expression data to overcome the heterogeneity of spatial transcriptomics data. This procedure results in homogeneous multi-layer networks. Subsequently, it transforms spatial domain identification into multi-layer network clustering problem. Then, it employs joint learning to integrate constructed multi-layer networks by projecting cells into a subspace, where the low-dimensional and compatible features of cells are learned. To enhance the interpretability of features, MNMST employs self-representation learning (SRL) to construct an affinity graph of cells with cell features. Experimental results demonstrate that the proposed multi-layer network model not only outperforms state-of-the-art algorithms on identification of spatial domains, but also covers all spatial transcriptomics platforms, including 10 $\times$ Visium, osmFISH, STARMap, Stereo-seq, and Slide-seq V2. These results further prove that MNMST achieves the best performance for spatial domains identification, demonstrating that MNMST is promising for analyzing spatial transcriptomics data and understanding of tissue architecture and functions.

## Results

### Overview of the multi-layer network model for spatial domain identification

To smooth the reading and understanding of this study, we first describe the rationale of MNMST in this section (more technical description can be referred in the “Methods” section). For the sake of convenience, we use cells to represent measurement units in spatial transcriptomics, which can be freely replaced to spots in various platforms, such as 10$\times$ Visium.

For any spatial transcriptomics data, the traditional clustering approaches [16, 28, 29] first perform feature learning for spatial and expression information of cells and then identify spatial domains by clustering the low-dimensional features of cells. However, these algorithms are criticized for the undesirable performance because features of cells fail to model and characterize the intrinsic and indirect relations among cells. To overcome this limitation, network-based algorithms, such as DeepST [25], SpaGCN [20], and STAGATE [21], first construct cell graph(s) by integrating spatial, expression information, etc. And, these algorithms then learn features by exploiting topological structure of the constructed cell networks, thereby dramatically enhancing performance of spatial domain identification.

However, these network-based algorithms still have several typical limitations that urgently need to be addressed. First of all, current algorithms construct cell network by calculating distances of cell pairs with concatenated cells features of spatial, expression, and morphological information, which are criticized for ignoring heterogeneity of spatial transcriptomics data, reducing quality and reliability of cell networks. Second, even though many alternatives are available for measuring distances among cells, it is difficult to select an appropriate manner to precisely characterize distances of cells (how measurement of distance effects performance of algorithms is studied in the “Systematic investigation of parameters of multi-layer network model” section). Finally, the learned low-dimensional features of cells only preserve the topological structure of cell networks, which neglect relations among spatial and expression information, failing to fully characterize the structure of spatial domains.

To address these issues, we propose a multi-layer network model (MNMST) to characterize and identify spatial domains in spatial transcriptomics data by integrating gene expression and spatial information of cells (Fig. 1). Specifically, to remove heterogeneity of spatial transcriptomics data, MNMST constructs two cell networks for spatial and expression data, respectively. In this case, MNMST converts the heterogeneous data integration problem into the homogeneous graphs clustering problem. Furthermore, MNMST learns features of cells by jointly exploiting topological structure of cell spatial and expression network, where the relation between spatial and expression information is implicitly learned, thereby improving quality of features of cells. In details, as shown in Fig. 1, MNMST consists of three major components, i.e., cell multi-layer network construction, cell feature learning, and spatial domain identification. On multi-layer network construction issue, MNMST first employs Euclidean distance to construct cell spatial network by exploiting indirect topological structure. Then, it automatically learns cell expression networks by utilizing self-representation learning (SRL) with local preservation constraint, where cell spatial information is incorporated to augment expression profiles of cells (Fig. 1 (A); see the “Methods” section). In this case, MNMST not only avoids selecting measurements of distances for cells, but also enhances quality of constructed graph.

On the cell feature learning issue, current algorithms independently learn spatial and expression features of cells, which ignores the latent relations among them. MNMST simultaneously learns spatial and expression features of cells to overcome this limitation. It does so by jointly decomposing cell multi-layer networks with nonnegative matrix factorization, which ensures the compatibility and quality of features (Fig. 1 (B)). MNMST automatically learns an affinity graph of cells with low-rank and sparse constraint to perform spatial domain identification. This way improves interpretability of spatial domains (Fig. 1 (B)). On the basis of affinity graph, MNMST identifies spatial domains and downstream analysis (Fig. 1 (C)).

Notice that, as far as we know, MNMST is the first multi-layer network model for spatial domain identification, which provides an alternative for modeling and analyzing spatial transcriptomics data.

### Benchmarking MNMST against feature-based state-of-the-art baselines

**Fig. 2:**
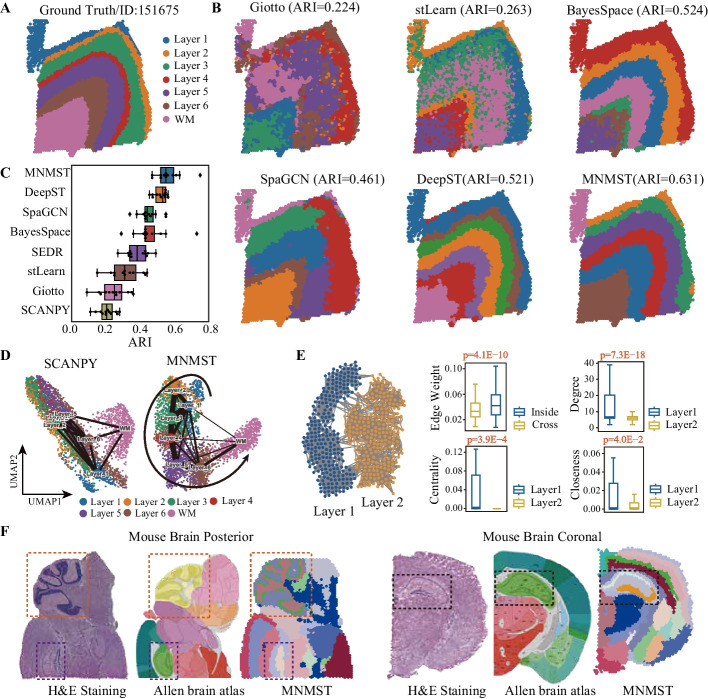
Multi-layer network model enhances spatial domain identification in human brain tissue. **A** Ground truth of spots is mapped to spatial position in slice 151675 of manually annotated DLPFC data [30], which consists of six cortical layers (L1–L6) and white matter (WM). **B** Visualization of spatial domains identified by state-of-the-art methods, including Giotto, stLearn, SpaGCN, BayesSpace, and MNMST, for slice 151675 of DLPFC. **C** Performance of algorithms for all 12 DLPFC slices, where *x*-axis denotes adjusted rand index (ARI) to quantify similarity of the predicted spatial layers and manually annotated layers. The center line, box limits, and whiskers denote the median, upper and lower quartiles, and 1.5 $\times$ interquartile range, respectively. **D** UMAP visualizations of PAGA graphs of slice 151675 for SCANPY (left) and MNMST (right). **E** Topology of sub-graph induced by L1 and L2 layers in cell affinity graph learned by MNMST (left), and distribution of edge weight, degree, eigenvector centrality, and closeness of cells in L1/L2 layers (right, Mann-Whitney *U* test for significance). **F** Spatial domains for sagittal posterior and coronal regions in mouse brain. H&E staining generated from raw data (left), corresponding anatomical Allen Mouse Brain Atlas (middle, https://atlas.brain-map.org/), and identified spatial domains (right). The black box denotes the cornu ammonis and dentate gyrus areas in the coronal portion, orange one denotes the cerebellar cortex, and purple box denotes dentate gyrus areas in the sagittal posterior

Human dorsolateral prefrontal cortex (DLPFC) data [30] consist of manually annotated cortical layers (L1–L6) and white matter (WM) of 12 slices with gene markers and cytoarchitecture. These data are generated with 10 $\times$ Visium platform, serving as a publicly available benchmarking dataset for spatial domain identification (Fig. 2A). Seven feature-based state-of-the-art algorithms, namely SCANPY (with Leiden clustering) [16], Giotto [29], stLearn [28], BayesSpace [18], SpaGCN [20], DeepST [25], and SEDR [22], are selected as baselines to fully validate performance of MNMST. Notably, SCANPY is non-spatial, and others are spatial-based methods. Adjusted rand index (ARI) [31] is selected to measure the performance of algorithms (details in the “Methods” section).

Figure 2A visualizes slice 151675 of DLPFC with seven layers labeled with different colors, and MNMST is much more precise to identify these layers than baselines (Fig. 2B). Specifically, ARI of MNMST is 0.631, whereas that is 0.224 for Giotto, 0.263 for stLearn, 0.524 for BayesSpace, 0.461 for SpaGCN, and 0.521 for DeepST, respectively. Interestingly, SpaGCN, DeepST, and MNMST dramatically outperform others, demonstrating that topological structure of cell networks is also critical for characterizing spatial domains. However, DeepST and SpaGCN independently learn spatial and expression features of cells and explore relations of cell features with post-processing techniques. However, they ignore the latent relations among features. MNSMT jointly learns features of cells by decomposing matrices associated with multi-layer networks to overcome this limitation, where the latent relations of features are implicitly explored, thereby enhancing quality of features. Notice that MNMST is the only algorithm to discriminate L1 and L2 cortical layers, which cannot be delineated by others (Fig. 2B).

Furthermore, the proposed algorithm is also superior to baselines on spatial domain identification for other slices of DLPFC (Additional file 1: Figs. S1-S2), and Fig. 2C summarizes performance of various algorithms for DLPFC data, where *x*-axis denotes ARI to measure similarity of the predicted spatial layers and manually annotated layers. These results demonstrate that spatial algorithms significantly outperform non-spatial methods, showing that spatial information is critical need for spatial domain identification. In details, ARI of SCANPY is 0.208 ± 0.049 (median ± standard deviation), whereas ARI of the worst spatial algorithm is 0.250 ± 0.078. Specifically, ARI of MNMST is 0.553 ± 0.073, whereas ARI of the best baseline DeepST is 0.515 ± 0.001. Moreover, MNMST obtains the best performance in slice 151670 (Additional file 1: Fig. S1, ARI = 0.750), 151674 (Additional file 1: Fig. S2, ARI = 0.610), 151675 (Additional file 1: Fig. S2, ARI = 0.631), and 151676 (Additional file 1: Fig. S2, ARI = 0.588). These results prove that MNMST is superior to DeepST and SpaGCN, showing that the proposed multi-layer network model is much precise than current network ones. To investigate why multi-layer network model is more discriminative than baselines for spatial domain identification, PAGA [32] is employed to infer relations of identified spatial domains, where organization of various cortical layers is derived. Figure 2D illustrates trajectory of layers, where spatial domains identified by MNMST are well discriminated, and those by SCANPY are mixed, demonstrating that network model precisely exploits intrinsic structure of spatial domains.

Strikingly, we find that spatial domains can be clearly characterized by topology of cell affinity graph learned by MNMST. Figure 2E (left) visualizes topology structure of sub-graphs included by L1 and L2 layer, where connectivity is strong within layers, and weak across layers, corresponding to clusters of graphs. Four topological indexes, namely edge weight, degree (sum of weights on adjacent edges to a vertex), eigenvector centrality (importance of vertices with the largest eigenvector), and closeness (number of edges through a vertex), are selected to validate the difference between L1 and L2 layers for investigating the relation of spatial domains and topological structure of the affinity graph (right panel of Fig. 2E). Specifically, weights on edges across layers are much lower than those inside layers (*p *= 4.1E−10, Mann-Whitney *U* test). Furthermore, degrees, centrality, and closeness of cells in L1 and L2 layers significantly differ (degree: *p *= 7.3E−18, centrality: *p *= 3.9E−4, closeness: *p *= 4.0E−2, Mann-Whitney *U* test). These results demonstrate that the topological structure of cell networks is highly associated with spatial domains, providing an alternative for analyzing spatial transcriptomics data.

Next, spatial transcriptomics data of mouse brain tissue generated by 10 $\times$ Visium platform is also adopted to evaluate the performance of MNMST. Brain anatomical references from the Allen Mouse Brain Atlas [33] are selected as baselines. MNMST precisely detects the cornu ammonis in mouse brain, and cerebellar cortex region in the sagittal posterior (Fig. 2F), which matches reference annotations [33]. Spatial domains identified by various algorithms for mouse brain tissue, and Silhouette Coefficient (SC) and Davies-Bouldin (DB) scores of them demonstrate that stLearn, DeepST, and MNMST are comparable (Additional file 1: Fig. S3). stLearn integrates spatial, transcriptional, and morphological information to comprehensively characterize structure of spatial domains in brain, whereas MNMST and DeepST fully exploit topological structure of cell networks for spatial domains. However, boundary of spatial domains identified by stLearn is non-smooth because it fails to fully exploit relations among spatial, transcriptional, and morphological information. These results show that the proposed multi-layer network model is also promising for characterizing complex spatial structure in mouse brain slices.

### Systematic investigation of parameters of multi-layer network model

MNMST learns features of cells with matrix factorization for multi-layer networks, implying that the theoretical complexity of MNMST is expensive (Additional file 1: Section 1.5). The comparative comparison of various algorithms on the time and space complexity is performed with different spatial transcriptomics data, where Giotto and BayesSpace are slower than stLearn and MNMST, and SCANPY, SpaGCN, and DeepST are faster than others (Additional file 1: Section 1.6 and Fig. S4). Specifically, running time of MNMST for DLPFC data is 4.3 ± 2.5 minutes, while that is 10.6 ± 2.9 (Giotto), 6.3 ± 2.2 (BayesSpace), 3.6 ± 0.4 (stLearn), 2.2 ± 0.4 (DeepST), 1.4 ± 0.3 (SpaGCN), 0.6 ± 0.1 (SEDR), and 0.1 ± 0.0 (SCANPY) respectively (Additional file 1: Fig. S4 A1). And, these results further demonstrate that MNMST achieves the best performance by reaching a good tradeoff between space and running time, proving its superiority for spatial domain identification. Furthermore, MNMST can also be accelerated with hardware, extending its applicability to large data (Additional file 1: Section 1.6 and Fig. S4).

**Fig. 3:**
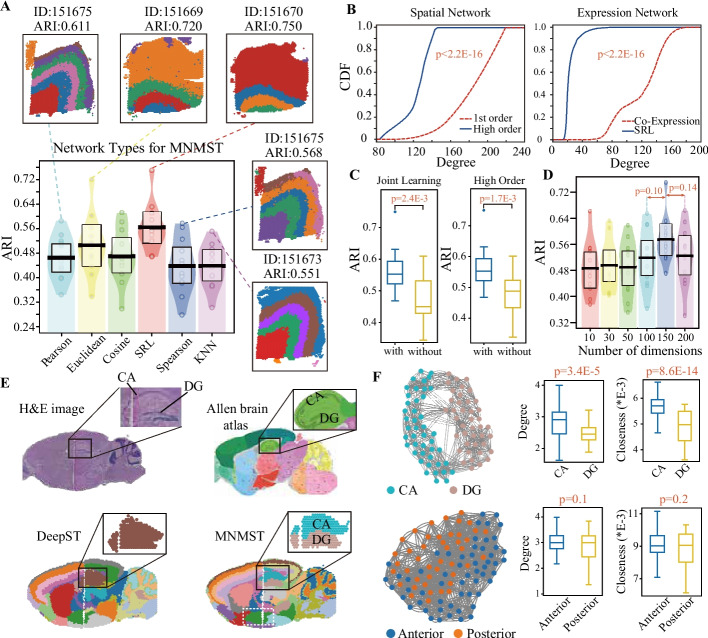
Systematic parameter analysis of multi-layer network model, and integrative analysis of spatially omics data. **A** Pirateplot of ARIs of MNMST with various types of cell networks for 12 DLPFC slices, where *y*-axis represents ARI, and *x*-axis denotes networks. The slice corresponding to the best performance for each types of cell networks are visualized. Pirateplot: points, raw data; center line, median; band, inference interval. **B** Cumulative distribution function (CDF) of degree of 1-st and high order cell spatial network (left), and of cell co-expression and expression network learned by MNMST (right), where *x*-axis denotes degree, and *y*-axis represents the probability for a random variable whose degree is less or equal to the given degree (Kolmogorov-Smirnov test for significance). **C** Distributions of ARIs of MNMST with and without joint learning (left), and distributions of ARI of MNMST with high- and 1st-order structure of cell spatial network (right, Student’s *t*-test for significance). The lower and upper hinges correspond to the first and third quartiles and the center refers to the median value. The upper (lower) whiskers extend from the hinge to the largest (smallest) value no further (at most) than 1.5$\times$ interquartile range from the hinge. **D** ARI pirateplot of MNMST vs the number of dimensions of cell features (Student’s *t*-test for significance). Pirateplot points, center line, and band are defined the same as in **A**. **E** H&E images of mouse anterior and posterior brain Visium data of 10 $\times$ Genomics, which are horizontally aligned (top left). The corresponding anatomical Allen Mouse Brain Atlas (top right, https://atlas.brain-map.org/). Spatial domains identified by DeepST and MNMST (bottom), where regions surrounded by squares are domains split by different slices. **F** Topological structure of cells in cornu ammonis (*CA*) and dentate gyrus (*DG*) domains (top left). Distribution of degrees of cells (top middle) and closeness (top right) in *CA* and *DG* domains. Topological structure of cells in domain split by slices (surrounded by white dashed line in **E**, bottom left), and distributions of degrees (bottom middle) and closeness (bottom right) of cells in anterior and posterior regions (Student’s *t*-test for significance). Boxplot hinges, median, and whiskers are defined the same as in **C**

MNMST constructs the cell affinity network with self-representation learning (SRL) and sparsity constraints, whereas baselines directly utilize cell co-expression networks by calculating the similarity of cells [16, 25]. Then, we check how performance of MNMST changes by replacing the learned cell affinity network with five cell co-expression networks (Spearman, Pearson, KNN, Cosine, and Euclidean distance). Figure 3A depicts ARIs of MNMST with various cell networks on DLPFC data (SRL: 0.553 ± 0.073 vs KNN: 0.446 ± 0.069, *p *= 3.5E−4, Student’s *t*-test). SRL achieves the highest ARI value than others, which indicates that MNMST is more precise on characterizing the structure of spatial domains than current baselines. Furthermore, various cell co-expression networks result in distinct hierarchical structure for the same slice (151675), where ARI of Pearson is 0.611 and that of KNN is 0.435 for slice 151675. Therefore, spatial domains cannot be fully characterized with cell co-expression network. Then, we investigate the difference between cell networks learned by MNMST and those used by baselines by checking the degree distribution of networks with cumulative distribution function (CDF), which calculates probability of random variable if it is less or equal a threshold. Figure 3B demonstrates that the learned networks significantly differ from these used by baselines (p<2.2E-16, Kolmogorov-Smirnov test), accounting for why the proposed algorithm is promising for spatial domain identification.

Since MNMST jointly learns features of cells, we compare the ARI of MNMST with/without joint learning (Fig. 3C left panel), where joint learning strategy is promising for learning features of cells (joint = 0.553 ± 0.073 vs non-joint = 0.450 ± 0.078, *p*-value = 2.4E−3, Student’s *t*-test). It shows that joint learning of cell spatial and expression networks enhances quality of features of cells because it implicitly reconciles spatial and expression information. Since MNMST utilizes high-order topology structure of cell spatial network for spatial domains (details in the “Methods” section), we also compare the ARI of MNMST with 1st-order and high-order structures of networks. Right panel of Fig. 3C shows that the high-order structure of networks is significantly more precise than 1st-order for spatial domain identification (high-order: 0.553 ± 0.073 vs 1st-order: 0.488 ± 0.077, *p *= 1.7E−3, Student’s* t*-test). The reason is that the relations among cells in spatial transcriptomics data are non-linear, which cannot be fully characterized with the low-order structure.

We systematically evaluate the hyperparameters of MNMST with various spatial transcriptomics data. How dimensional of cell features effect performance of MNMST is shown in Fig. 3D, where the number of dimensions in [100, 200] is a good choice because performance of MNMST is stable (Student’s *t*-test). Furthermore, parameter effect analysis not only demonstrate that MNMST is quite stable, but also suggest the values of parameters for typical spatial transcriptomics data (Additional file 1: Fig. S5 and Section 1.7).

### Multi-layer network provides an effective and efficient strategy for integrative analysis of spatial transcriptomic data

Extensive applications of spatial sequencing technologies generate a great mount of spatially omics data, which poses a great challenge on their integrative analysis. Given that current algorithms fail to integrate spatially omics data, it is interesting to design algorithms for integrating multiple datasets from various technologies. MNMST provides a novel strategy for integrative analysis of spatial transcriptomics data from three perspectives, i.e., joining multiple sections of whole tissues, integrating multiple slides of specific tissues, and integrating datasets from various batches.

Large tissues require multiple slides to cover different sections, and we employ PASTE [34] to horizontally align the tissue slices for ensuring their spatial adjacency. Then, MNMST directly performs spatial domain identification on aligned slices. Mouse brain Visium datasets of 10 $\times$ Genomics (https://www.10xgenomics.com/), including anterior and posterior brain slices, are selected to validate performance of algorithms, where aligned slices are shown in Fig. 3E (left top panel), and zoomed regions correspond to *CA* (cornu ammonis) and *DG* (dentate gyrus). The annotation of domains for aligned slices is from the Allen Mouse Brain Atlas (right top panel of Fig. 3E). Visualization of spatial domains obtained by DeepST and MNMST is shown in Fig. 3E (bottom panels). MNMST significantly outperforms baselines since it precisely discriminates *CA* and *DG* regions, where these domains are mixed (Additional file 1: Fig. S6). It demonstrates that MNMST preserves the structure of spatial domains across various slices, which implies that the multi-layer network model is promising for integrating spatially omics data.

Visualization of topological structure of the affinity graph of cells is shown in Fig. 3F (left top panel), where cells with the same color form a domain, to determine the reason behind the superiority of MNMST over baselines. Obviously, *CA* and *DG* correspond to two clusters in the affinity graph learned by MNMST, indicating that the topological structure of cells facilitates the identification of spatial domains. Specifically, degrees of cells in *CA* are significantly higher than those of *DG* (top middle panel of Fig. 3F, *CA*: 2.7 ± 0.4 vs *DG*: 2.4 ± 0.1, *p *= 3.4E−5, Student’s *t*-test), as well as in closeness (top right panel of Fig. 3F, *p *= 8.6E−5, Student’s *t*-test). Therefore, MNMST maintains consistence of the separation boundary of spatial domains across various slices in terms of topological structure of affinity graph. Moreover, we investigate whether MNMST can preserve the structure of domains split by various slices (surrounded by white dashed line in the right bottom panel of Fig. 3F). Visualization of the topological structure of affinity graph is shown in Fig. 3F (left bottom panel), where cells are mixed given that they belong to the same spatial domain. Topological structure of cells in the anterior and posterior slice is subtle (bottom panel of Fig. 3F). These results prove that MNMST is efficient for horizontally integrating spatially omics data.

Then, we hypothesize that vertically adjacent slices are very similar, and integrate them to facilitate spatial domain identification. The proposed multi-layer network model naturally provides an effective strategy for integration of multiple vertically adjacent slices. Specifically, MNMST first stacks multiple slices into one as input, and then learns the affinity graph from the stacked slice to promote identification of spatial domains. Experiments demonstrate that integrative analysis enhances performance of spatial domain identification on DLPFC data (151673, 151674, 151675 and 151676, Additional file 1: Fig. S7 A). In details, ARI of spatial domains after integration is 0.570 for slice 151673, whereas it is 0.554 before integration, which shows that MNMST precisely captures complementary information from adjacent slices to promote spatial domain identification.

Moreover, we also investigate capability of MNMST for vertical integration with MERFISH data, where DeepST and MNMST achieve the comparable performance, demonstrating that multi-layer network model is insensitive to platforms (ARI: 0.493 vs 0.500, Additional file 1: Fig. S7B). These experiments for integrative analysis of spatial transcriptomics data are independently performed by MNMST. Thus, it is natural to ask whether MNMST can combine with other algorithms, such as PASTE [34], to perform integrative analysis. In other words, MNMST takes data integrated by PASTE [34] as inputs and then performs spatial domain identification. Notably, MNMST outperforms DeepST on the center slice generated by PASTE, where ARI of MNMST and DeepST is 0.597 and 0.537 respectively, demonstrating that multi-layer network model is more precise to capture intrinsic structure of spatial domains (Additional file 1: Fig. S7C). Furthermore, MNMST also achieves an excellent performance on the vertical stacking slices generated by PASTE (Additional file 1: Fig. S7D).

Then, we investigate the capability of MNMST to remove batch effect from spatial transcriptomics datasets. Up-to-date methods (DeepST, stLearn, and SEDR) address these issues with additional computational modules, while MNMST can be directly applied without modifying algorithms. Specifically, MNMST first stacks multiple slices from various batches into one slice with SCANPY [16] and then constructs cell spatial network and cell expression network for the integrated slice, where cross-slice spatial proximity of spots enhances consistence of features of adjacent spots, thereby removing batch effect at some extent. With four DLPFC slices (1516173-151676), MNMST achieves the best performance, where the ARI of MNMST is 0.609 (ARI of DeepST: 0.448, ARI of stLearn: 0.248, and ARI of SEDR: 0.421, Additional file 1: Fig. S8A). Interestingly, integration of multiple slices enhances the performance of spatial domain identification, which shows that MNMST can effectively remove batch effect by retaining biological content. Except for DLPFC data, mouse slices with breast cancer are aligned vertically using PASTE, where there is obvious batch effect (Additional file 1: Fig. S8B). It is observed that spatial domains in vertically adjacent slices are highly consistent after integration, whereas these domains are mixed without integration (Additional file 1: Fig. S8). These results demonstrate that MNMST effectively leverages spatial information to correct batch effects in spatial transcriptomics data.

### Multi-layer network model precisely dissects cancer and non-cancer spatial domains

**Fig. 4:**
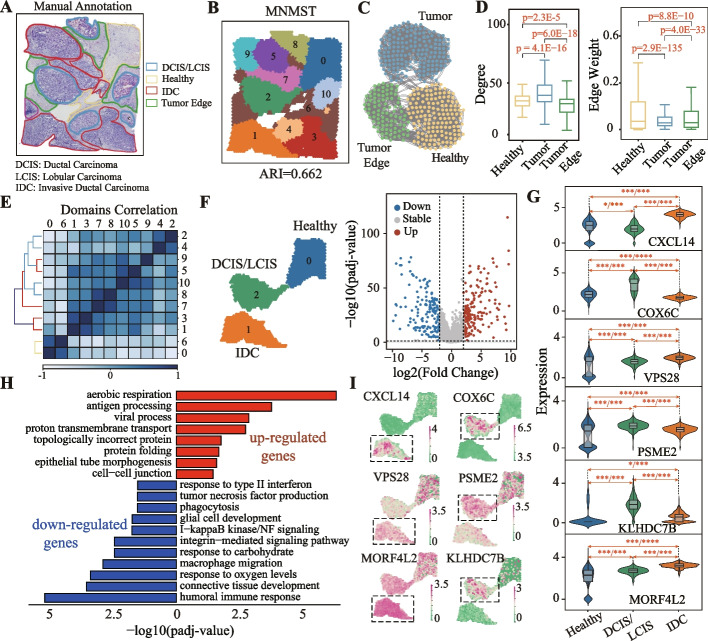
MNMST precisely dissects cancer and non-cancer spatial domains. **A** Visium spatial transcriptomics data of breast cancer samples annotated by pathologists with different regions, i.e., IDC (invasive ductal carcinoma), DCIS (ductal carcinoma in situ), LCIS (lobular carcinoma in situ), tumor edge, and healthy. **B** Spatial domains identified by MNSMT on human breast cancer with *domains* = 11. **C** Visualization of topological structure of sub-graph induced by cells in healthy, tumor, and tumor edge domains identified by MNMST. **D** Distribution of degrees (left) and edge weight (right) of cells in cell affinity graph learned by MNMST for healthy, tumor, and tumor edge domains (Student’s *t*-test for significance. Box plot: center line, median; box, interquartile range; and whiskers, minimum-maximum range). **E** Heatmap of Pearson correlation coefficient among domains. **F** Differential expression analysis among domains 0, 1, and 2, which corresponds to Healthy, IDC, and DCIS/LCIS regions (left); volcano graph of differentially expressed genes (DEGs) between domains 1 and 2, where* x*-axis denotes log2(Fold Change) and *y*-axis represents -log10(*p*-value) (right). **G** Distributions of expression levels of DEGs ($$\vert$$log fold change$$\vert \ge 2$$) between domains 1 and 2 (left), and spatial distribution of expression of DEGs between domains 1 (orange) and 2 (green), where $$*$$ denotes* p *< 5E−2, $$**$$ *p *< 1E−2, and $$***$$ *p *< 1E−3 (Wilcoxon rank-sum test. Dashed line in violin plot: $$\frac{1}{4}$$ and $$\frac{3}{4}$$ quantiles. Concrete line: median). **H** Gene ontology enrichment analysis of DEGs between domains 1 and 2, where red denotes functions enriched by upregulated genes, and blue by downregulated ones. **I** Spatial distribution of expression of DEGs between domains 1 and 2

Spatial transcriptomics technologies are successfully applied to cancers, and the generalization power of the proposed multi-layer network model with cancer data can be naturally investigated. The public spatial transcriptomics data of human breast cancer generated by 10 $\times$ Visium, consisting of 3798 spots and 36,601 genes, is selected to validate the capability of identifying cancer-related spatial domains (Fig. 4A). The dataset is manually annotated by pathologists based on H&E image and the spatial expression of reported breast cancer marker genes, which consists of 20 regions and 4 main morphotypes, i.e., ductal carcinoma in situ/lobular carcinoma in situ (DCIS/LCIS), healthy tissue (Healthy), invasive ductal carcinoma (IDC), and tumor edge (Fig. 4A). Spatial domains identified by MNMST are highly consistent with the manual annotations, with an ARI of 0.662 (Fig. 4B), whereas domains obtained by baselines exhibit less regional continuity and more outliers (Additional file 1: Fig. S9). These finding implies that multi-layer network model is also promising for characterizing and extracting cancer spatial domains.

However, state-of-the-art algorithms fail to precisely identify tumor edge regions because transition from healthy to tumor is difficult to characterize and capture, which poses a great challenge in learning discriminative features of cells. The proposed multi-layer network model captures differences among various domains because indirect topological relations among cells are exploited. Specifically, the topological structure of sub-graph induced by cells in healthy, tumor edge, and tumor domains is visualized in Fig. 4C, where spatial domains correspond to clusters in the learned affinity graph. Specifically, distributions of degrees of cells in these three domains differ greatly (Fig. 4C left, Healthy: 33.2 ± 11.7 vs Tumor: 42.0 ± 17.6, *p *= 4.1E−16, Tumor edge: 28.7 ± 10.0 vs Tumor: 42.0 ± 17.6, *p *= 6.0E−18, Student’s *t*-test), as well as in closeness (Fig. 4C right). These results further demonstrate that the multi-layer network model is also effective to characterize cancer spatial domains.

Heterogeneity of tumors is critical for the diagnosis and therapy of cancers [35]. Therefore, we hypothesize that tumor heterogeneity can also be reflected by cancer spatial domains. The Pearson correlation coefficient among domains is shown in Fig. 4D, where domains are clearly divided into two groups, i.e., tumor (domain 0, 6), and non-tumor (the rest ones). This result indicates that spatial domains can serve as bio-markers to characterize tumor heterogeneity. Moreover, we perform differentially expressed analysis and gene ontology enrichment analysis to identify differentially expressed genes (DEGs) by comparing transcriptional differences among spatial domain 0 (Healthy), spatial domain 1 (IDC), and spatial domain 2 (DCIS/LCIS) (Fig. 4F, details in the “Methods” section).

To obtain intra-tumoral transcriptional differences of genes, we detect 396 DEGs ($\vert$log fold change$\vert$ $\ge$ 2, adjusted *p *< 5.0E−2, Wilcoxon rank-sum test for significance corrected by Benjamini-Hochberg test) between domains 2 and 1 (Fig. 4E–G, Additional file 1: Fig. S10 and Additional file 2). Functions enriched by DEGs are listed in Fig. 4H, where upregulated genes are involved in immune-related pathways, and downregulated ones are associated with signal pathways of fibroblast proliferation (*p *= 4.2E5, Hyper-geometric test). *CXCL14* encodes secreted proteins involved in immuno-regulatory and inflammatory processes, which promotes tumor growth in breast cancer [36], and *COX6C* serves as a bio-marker for cancer therapy [37]. Interestingly, these genes are captured with spatial domains as shown in Fig. 4I, where *CXCL14* is upregulated in IDC, and *COX6C* is depressed in IDC. Furthermore, *APOE*, *APOC1*, *C1QA*, *C1QB*, and *NUPR1* are indicative markers of variations in the infiltration of tumor-associated macrophages (TAM) [38, 39], where TAM infiltration is known to be associated with unfavorable survival outcomes in solid tumors. This association is owing to its role in promoting tumor angiogenesis, which induces tumor migration, invasion, and metastasis [40–42].

Then, we investigate whether spatial domain DEGs are associated with survival time of patients by employing Kaplan-Meier survival analysis with gene expression profiles and clinical data of breast cancer in The Cancer Genome Atlas (TCGA). We find that 31 of 396 DEGs separate patients into high- and low-risk groups with significant survival time (Additional file 1: Fig. S12). For example, *MORF4L2*, *PKG1*, *VPS28*, and *KLHDC7B* predict the survival time of patients, indicating that cancer domains also facilitate the identification of bio-markers for breast cancer. For example, it is proven that *KLHDC7B* regulates interferon signaling pathway, which is critical for breast cancer tumorigenesis [43].

Analogously, we perform differential expression analysis by comparing domain 1 vs 0 and identify some interesting DEGs that are highly associated with breast cancer (Additional file 1: Fig. S10, Additional file 2,3). For example, *PRDX1* participates in the signaling pathway of fibroblast proliferation, which serves as a bio-marker to characterize progression and metastasis of human breast cancer [44]. Additionally, upregulated *B2M* is also associated with a poorer prognosis [45], and *NECTIN2* is a potential target of antibody therapy for breast cancer [46]. Furthermore, gene analysis between domains 2 and 0 indicate that *NUPR1* is upregulated, which is linked to chemotherapy resistance of breast cancer (Additional file 1: Fig. S10 and S12, Additional file 2) [47]. These results demonstrate that multi-layer network model also effectively identifies cancer spatial domains, which may shed light on revealing underlying mechanisms of cancers.

### Multi-layer network model is applicable for spatial omics data with various platforms

Except for the 10 $\times$ Genomics Visium platform, we further determine the applicability of the proposed multi-layer network model with spatial transcriptomics data from three additional platforms, i.e., imaging-based molecular data (mFISH [48, 49]) and high-resolution spatial transcriptomics data (Stereo-seq [12] and Slide-SeqV2 [50]).

**Fig. 5:**
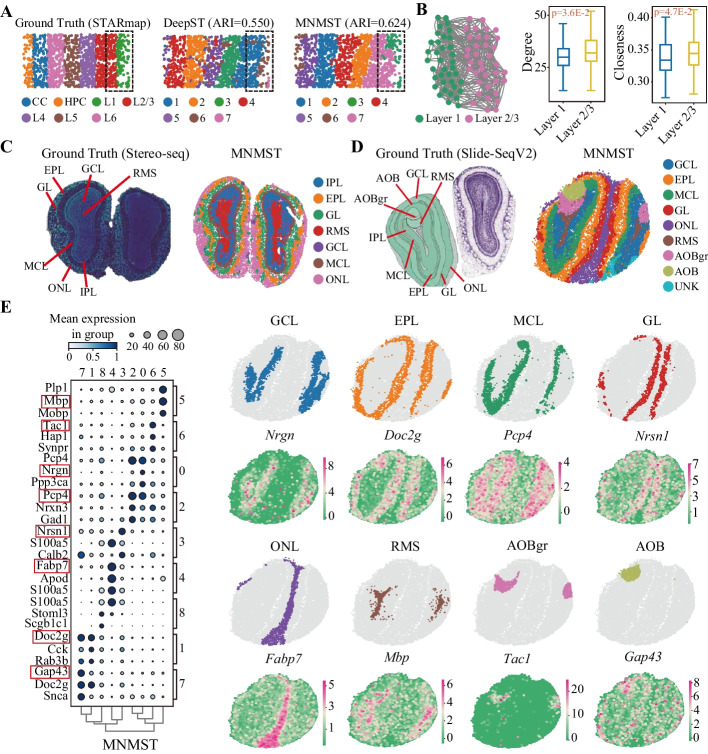
Multi-layer network model handles spatial data with various platforms. **A** Visualization of lattice-shaped STARmap data for mouse visual cortex [48] with 7 layers, such as CC, HPC, L1, L2/3, L4–L6 (left). Spatial domains identified by DeepST (middle, ARI = 0.550) and MNMST (right, ARI = 0.624), where L1 and L2/3 domains are surrounded by a dashed squares. **B** Topological structure of the affinity graph for cells in Layer1 and Layer 2/3 (left), and distributions of degrees (middle) and closeness (right) between layer 1 and layer 2/3 (Student’s *t*-test for significance. Box plot: center line, median; box, interquartile range; and whiskers, minimum-maximum range). **C** Visualization of spatial transcriptomics data generated with Stereo-seq platform (left), and spatial domains identified by MNMST (right), where RMS denotes rostral migratory stream, ONL is olfactory nerve layer, IPL is internal plexiform layer, GL is glomerular layer, MCL is mitral cell layer, GCL is granule cell layer, and EPL is external plexiform layer. **D** Visualization of spatial transcriptomics data generated with Slide-SeqV2 platform (left), and spatial domains identified by MNMST (right). **E** Dotplot of top 3 DEGs of domains in mouse olfactory bulb data generated from Slide-SeqV2 (left). Scatter plot of spatial domains generated by MNMST (right, including genes *Nrgn*, *Doc2g*, *Pcp4*, *Nrsnl*, *Fabp7*, *Mbp*, *Tacl*, and *Gap43*)

The imaging-based molecular data consist of lattice- and non-lattice-shaped structure, and the lattice-shaped STARmap data for mouse visual cortex is selected, which contains 1207 cells, 1020 genes in each cells, and 7 layers as shown in Fig. 5A. MNMST outperforms baselines with an ARI of 0.624 (Fig. 5A right, Additional file 1: Fig. S14A), whereas that of DeepST is 0.550. Notably, MNMST successfully identifies *L1* and *L2/3* layers, whilst baselines fail to discriminate these layers (surrounded by a dashed square in Fig. 5A). We further investigate the difference of cells in *L1* and *L2/3* layers in terms of topological structure of networks, where distributions of degrees of cells significantly differ (degree L1: 30.9 ± 8.6 vs L2/3: 34.1 ± 10.9, *p *= 3.6E−5, Student’s *t*-test), so does closeness. These results demonstrate that MNMST can also precisely model spatial domains in lattice-shaped STARmap data.

Given that some out-of-date algorithms, such as BaysecSpace [18], are deliberately designed for lattice-shaped spatial datasets, we evaluate performance of MNMST with non-lattice-shaped spatial transcriptomics data generated by osmFISH [49], where spatial domains are labeled with different colors (Additional file 1: Fig. S14B). MNMST substantially outperforms baselines for non-linear and non-convex spatial domains with ARI = 0.600, whereas that of DeepST 0.490 (Additional file 1: Fig. S14C). Specifically, MNMST effectively distinguishes layer 2–3 lateral and medial regions that cannot be discriminated by baselines. The visualization of topological structure of different spatial domains demonstrate that these regions are precisely modeled by the cell affinity graph learned by MNMST, and cells within different domains significantly differ in terms of topological indexes (Additional file 1: Fig. S14D). For example, significant difference of cell degree between *Lateral* and *Medial* regions is observed (*Lateral*: 2.0 ± 0.7 vs *Medial*: 2.9 ± 1.0, *p *= 2.5E−35, Student’s *t*-test). These results prove that the multi-layer network model is also effective for identifying spatial domains in imaging-based molecular platform.

Then, we apply MNMST to the Stereo-seq data from mouse olfactory bulb tissues [12], which fulfills subcellular spatial resolution using DNA nano-ball patterned array chips. It contains the rostral migratory stream (RMS), granule cell layer (GCL), internal plexiform layer (IPL), mitral cell layer (MCL), external plexiform layer (EPL), and olfactory nerve layer (ONL) (Fig. 5C left panel). MNMST precisely identifies spatial domains, where the laminar organization is consistent with annotation of layers (Fig. 5D right panel). In particular, MNMST accurately recognizes the narrow tissue structure MCL, which is validated with the expression of mitral cell marker *Scg2*. Moreover, we identify bio-marker genes for other domains, including *Pcp4* for IPL, *Olfm1* for EPL, and *Cck* for GL, respectively (Additional file 1: Fig. S14C).

Finally, we apply MNMST to a mouse olfactory bulb slice profiled by Slide-SeqV2 [50], where the annotation is from the Allen Reference Atlas [33] (Fig. 5D left panel). Spatial domains obtained by MNMST are highly consistent with annotations (Fig. 5D right panel). Specifically, MNMST accurately identifies the accessory olfactory bulb (AOB), and granular layer of the accessory olfactory bulb (AOBgr). We examine the expression levels of bio-marker genes for each domain to validate the identified spatial domains, and we find that these domains identified by MNMST are well supported by the spatial variable genes (Fig. 5E). For example, *Pcp4* is upregulated in MCL, which is consistent with previous biological experiments [51]. Collectively, these results demonstrate that the multi-layer network model precisely identifies domains in spatial domains regardless of platforms, showing the superiority of network-based model.

## Discussion

In this study, we propose a novel multi-layer network model (MNMST) to integrate gene expression and spatial information of spatial transcriptomics data for spatial domain identification. MNMST is extensively evaluated on various data from various species and tissues. Extensive experimental results demonstrate that MNMST not only precisely identifies spatial domains (Fig. 2), but also is applicable to spatial transcriptomics data generated from various platforms, such as 10 $\times$ Genomics, mFISH, Stereo-seq, and Slide-SeqV2 (Fig. 5). Furthermore, MNMST precisely dissects cancer-related domains from cancer spatial transcriptomics data (Fig. 4).

The proposed multi-layer network model constructs the cell spatial and expression network and then performs feature learning and spatial domain identification by exploiting the structure of networks. MNMST serves as a flexible framework for spatial transcriptomics data, which can be easily extended for particular situations. First, although MNMST automatically learns cell multi-layer networks from spatial transcriptomics data, it also takes available networks as inputs. Therefore, MNMST offers users an opportunity to construct cell networks by incorporating expert-knowledge with particular backgrounds. Second, MNMST simultaneously addresses spatial transcriptomics data regardless of platforms such that users can directly apply it to available data without modifying the structure of algorithms. Third, MNMST learns an affinity graph of cells, where spatial domains correspond to clusters of the graph. It therefore improves the interpretability of spatial domains, facilitating the down-stream analysis of spatial transcriptomics data. Finally, although MNMST mainly focuses on the integration of gene expression profiles and spatial information to identify spatial domains, it can be easily extended to integrate supplementary information, such as morphological information, by constructing additional networks.

Rapid developments of spatial transcriptomics technologies can measure large number of cells with high spatial resolutions at an unprecedent speed, which consequently results in the explosion of data. However, cell multi-layer networks constructed by MNMST is proportional to the number of cells, which poses a great challenge in accelerating MNMST for large- and super large-scale cell networks with millions of cells, hampering its application to very large data. Therefore, it is one of important issues to accelerate the proposed multi-layer network model. We already show that hardware acceleration is a good choice (Additional file 1: Section 1.6 and Fig. S4C). There are multiple possible strategies to address this issue, i.e., parallel computation or distributed learning systems, as well as approximating computation. Moreover, integrating genomics and spatial omics to further enhance the resolutions and annotation of spatial domains is also interesting.

## Conclusion

In conclusion, we design a promising and novel approach MNMST that accurately identifies spatial domains with the network-based model,. This method facilitates the identification of tissue organization and enables the discovery of corresponding gene markers, providing an effective and efficient model to understand complex biological systems in a spatial context. In contrast to existing approaches, MNMST demonstrates its superiority for spatial domain identification and allows for the integrative analysis of spatial transcriptomics across multiple tissue sections. We demonstrate the benefits of MNMST through comprehensive experiments with various spatial transcriptomics data generated by different platforms, providing a novel multi-layer network model for spatial transcriptomics data.

## Methods

### Data preprocessing

For all datasets, spots (cells) outside the primary tissue regions are removed. By using SCANPY package [16], the raw expression data is filtered and normalized with log-transformed according to library sizes. By following Seurat [52], genes that are expressed in less than 10 spots are also filtered. Only top 3000 genes remain for downstream analysis according to variance of gene expression, and expression of each spot is enhanced with its adjacent ones with BANKSY [24]. Principal component analysis (PCA) is employed for dimension reduction on the augmented gene expression profiles to obtain the low-dimensional cell expression matrix $X\in R^{n\times \iota }$, where *n* and $\iota$ is the number of cells and dimensions respectively (usually $\iota$=50 [20, 25]).

### Construction of cell multi-layer networks

MNMST constructs a graph for each of spatial and expression information in spatial transcriptomics data, resulting in the multi-layer network $\mathcal {G}=\{G^{[s]}, G^{[e]}\}$, where $G^{[s]}$ is the cell spatial network, and $G^{[e]}$ corresponds to the cell expression network. The adjacent matrix of a cell network *G* is denoted by $W\in R^{n\times n}$, where element $w_{ij}$ denotes weight on edge connecting the *i*-th and *j*-th cell. Thus, constructing a network is equivalent to obtaining its adjacent matrix.

On the construction of cell spatial network $G^{[s]}$, MNMST first constructs a initial cell spatial network $W^{[s]}$ with using *K*-nearest neighbor algorithm (by following Squidpy [53], the number of neighbors *k* is 6 for 10 $\times$ Genomics data, 8 for Slide-seq and Stereo-seq data, and 15 for imaging-based platforms), where weight $w_{ij}^{[s]}$ for the *i*-th and *j*-th cell is inverse proportional to the spatial Euclidean distance between them (denoted by $r_{ij}$), i.e., $w_{ij}^{[s]}=(r_{ij})^{-1}$. Recently, evidence demonstrates that indirect relations, such as multiple hops among cells, is more precise characterize topological structure of network [54]. MNMST takes pointwise mutual information (PMI) matrix [55] as adjacent matrix of cell spatial network $G^{[s]}$ (denoted by $M^{[s]}$). And, element $m_{ij}^{[s]}$ of $M^{[s]}$ is defined as

1 $$\begin{aligned} m_{ij}^{[s]}=log \frac{w_{ij}^{[s]} \sum _l d_l}{d_i d_j} - log\kappa \end{aligned}$$

where $d_l$ is the degree of l-th cell in network $W^{[s]}$, and $\kappa$ is the number of non-negative samples (default set to 1). In this study, we refer PMI matrix $M^{[s]}$ as the high-order topological structure of network *G*.

On the construction cell expression network $G^{[e]}$, the cell expression matrix *X* is first augmented with the initial cell spatial network $W^{[s]}$ according to Ref. [24]. Then, MNMST utilizes sparse self-representation learning (SRL) [56–58] with local preservation constraint [59] to automatically learn adjacent matrix $W^{[e]}$, which is under the assumption that each cell can be well represented with its neighbors. And, the objective function for constructing cell expression network $G^{[e]}$ is formulated as a minimization problem as

2 $$\begin{aligned}{} & {} \min \left\| X-XW^{[e]} \right\| ^2+\alpha \left\| W^{[e]} \right\|_{1}+\beta {Tr \left(W^{[e]}L{W^{[e]}}^{\prime} \right)}\nonumber \\{} & {} \text {s.t.} \quad W^{[e]}\ge 0, \quad diag \left(W^{[e]}\right) = 0, \end{aligned}$$

where $L^{[e]}=D^{[e]}-W^{[e]}$ is the Laplacian matrix, *Tr*(*A*) denotes the trace of matrix *A* (i.e., $Tr(A)=\sum _{i}a_{ii}$), $\Vert \cdot \Vert ^2$ and $\Vert \cdot \Vert _1$ are $l_2-$ and $l_1$-norm respectively, and $\alpha$ and $\beta$ are parameters. The constraint $diag(W^{[e]})=0$ avoids trivial solutions (formulation and optimization of Eq. 2 are shown in Additional file 1: Section 1.1 and 1.2).

### Spatial and transcriptional feature learning by factorizing cell multi-layer networks

Given cell multi-layer networks, the most intuitive strategy is to independently learn cell features for each graph, which are adopted by current algorithms because of simplicity. However, this strategy is criticized for the low quality of cell features considering that it neglects relations between cell spatial and expression network. In our previous study [60], we demonstrate that jointly factorizing multi-layer networks is promising in capturing the relations of various graph, which significantly enhances the quality of features. Herein, MNMST utilizes a joint model with non-negative matrix factorization [61] to decompose the cell multi-layer network as

3 $$\begin{aligned}{} & {} \min \left\| W^{[e]}-B{F^{[e]}} \right\| ^2+ \left\| M^{[s]}-B{F^{[s]}} \right\|^2\nonumber \\{} & {} \text {s.t.} \quad B \ge 0, F^{[e]} \ge 0, F^{[s]} \ge 0, \end{aligned}$$

where matrix *B* denotes the shared feature of cells.

Similar to Eq. 2, MNMST automatically learns an affinity graph based on matrix *B* with the denoising model and self-representation learning, which is formulated as

4 $$\begin{aligned} \min \Vert Z\Vert _*+\Vert E\Vert _{2,1}, \quad \text {s.t.} \quad B^{'} = B^{'}Z+ E, \end{aligned}$$

where $B'$ denotes the transpose of *B*, *E* is the error matrix, $\Vert Z\Vert _*$ is the nuclear norm of *Z* [62], and $\Vert E\Vert _{2,1}$ is the $l_{2,1}$-norm, respectively (mathematical model for Eq. 4 in Additional file 1: Section 1.1).

Independence of feature learning and affinity graph construction fails to characterize patterns in spatial omics data, resulting in unsatisfactory spatial domain identification performance entirely, i.e., MNMST without joint learning. Previous studies [15] demonstrate that joint learning is more precise to characterize structure of networks, and [63] verifies that joint learning is also applicable to spatial omics data. Inspired by previous studies, MNMST joints feature learning and affinity graph construction by combining Eqs. 3 and 4; objective function of feature learning is formulated as

5 $$\begin{aligned}{} & {} \left\| W^{[e]}-B{F^{[e]}} \right\|^2+ \left\| M^{[s]}-B{F^{[s]}} \right\|^2 + \gamma \Vert Z\Vert _{*}+\lambda \Vert E\Vert _{2,1}\nonumber \\{} & {} \text {s.t.} \quad B\ge 0, F^{[e]} \ge 0, F^{[s]} \ge 0, B^{'}=B^{'}Z+E, \end{aligned}$$

where $\gamma$ and $\lambda$ are parameters for tuning. The Alternating Direction Method of Multipliers (ADMM) method [64] is employed to optimize Eq. 5, and update rules are deduced (Additional file 1: Section 1.3).

### Clustering and visualization

Based on the learned affinity graph, MNMST employs Leiden algorithm [65] to obtain graph partitioning, where each cluster corresponds to a spatial domain. If the number of spatial domains is known in advance, MNMST performs grid search with gap of 0.01 until the number of clusters is reached. Instability of nonnegative matrix factorization is adopted to obtain the number of domains when it is unknown [60]. To visualize distributions of cells in tissues, the uniform manifold approximation and projection (UMAP) algorithm is selected [66].

### Clustering criteria

When annotations of spatial domains are missing, two extensively adopted clustering criteria, including Silhouette Coefficient (SC) and Davies-Bouldin (DB) scores, are selected to validate the performance of clustering in terms of computation. Specifically, SC takes into account compactness within and separation across clusters as $(b-a) / max(a, b)$, where *a* is the mean intra-cluster distance, and *b* is the mean nearest-cluster distance. It ranges between −1 and 1, where a higher score refers to more coherent clusters. SC=0 means that the sample is on or close to the boundary of neighboring clusters, whereas negative values denote potentially wrong clusters. DB score is the average ratio of within-cluster distances to between-cluster distances, favoring farther apart and less dispersed clusters with low values.

### Spatial trajectory inference

PAGA [32] infers trajectories of cell types for single-cell transcriptomics data, which generates graph-like maps of cells by preserving continuous and disconnected structures at multiple resolutions. Specifically, it outputs a graph, where nodes are clusters and edges are connectivity (or similarity) of clusters. Here, we adopt it to infer relations of spatial domains by inputting affinity graph of cells as well as labels of domains.

### Identification and functional analysis of differentially expressed genes

MNMST performs differential expression analysis of genes for each spatial domain by using Wilcoxon rank-sum test implemented in SCANPY package [16]. Genes are expressed in 80% cells/spots in each domain and are with the absolute value of log fold change $\ge$ 2, and an adjusted FDR $\le$ 0.05 is selected as differentially expressed genes (DEGs). The overlapping genes between DEGs and highly variable genes (HVG) obtained by spatialDE [27] are used for down-stream analysis. Gene ontology enrichment analysis is performed with clusterProfiler [67].

### Benchmarking

Seven state-of-the-art methods, namely SCANPY [16], Giotto [29], stLearn [28], SEDR [22], BayesSpace [18], SpaGCN [20], and DeepST [25], are selected as baselines, where SCANPY is non-spatial, and others are spatial clustering algorithms. All these algorithms are executed with the suggested values of parameters to achieve the best performance for fair comparison.

For datasets with known annotations of spatial domains, given ground truth $P^{*}$ and predicted domains *P*, the adjusted rank index (ARI) is defined as [31]

$$\begin{aligned} ARI(P^*,P)=\frac{\sum \limits _{ij}\left( {\begin{array}{c}n_{ij}\\ 2\end{array}}\right) -\left[ \sum \limits _i \left( {\begin{array}{c}n_{i}\\ 2\end{array}}\right) + \sum \limits _j\left( {\begin{array}{c}n_{j}\\ 2\end{array}}\right) \right] \left/ \left( {\begin{array}{c}n\\ 2\end{array}}\right) \right.}{\frac{1}{2}\left[ \sum \limits _i \left( {\begin{array}{c}n_{i}\\ 2\end{array}}\right) + \sum \limits _j \left( {\begin{array}{c}n_{j}\\ 2\end{array}}\right) \right] - \left[ \sum \limits _i \left( {\begin{array}{c}n_{i}\\ 2\end{array}}\right) + \sum \limits _j \left( {\begin{array}{c}n_{j}\\ 2\end{array}}\right) \right] \left/ \left( {\begin{array}{c}n\\ 2\end{array}}\right)\right. }, \end{aligned}$$

where *n* is the number of cells, $n_{ij}$ is the number of cells of class label $C^{*} \in P^{*}$ assigned to cluster $C_i$ in partition *P*, and ${n_i}$ / ${n_j}$ is the number of cells in cluster $C_i$ / $C_j$ of partition *P*.

### Supplementary information

Additional file 1: Supplementary information. Supplementary notes describing the detailed derivations of the MNMST algorithm, and Supplementary figures.

Additional file 2: Supplementary table, differentially expressed genes (DEGs) identified between Domain 2 and Domain 1, Domain 2 and Domain 0, and Domain 1 and Domain 0 in human breast cancer dataset.

Additional file 3: Supplementary table, gene ontology enrichment analysis results corresponding for DEGs in Additional file 2.

Additional file 4: Review history.

## Data Availability

The code for the MNMST algorithm is implemented in Python and detailed tutorials are freely available at GitHub [68] as well as Zenodo [69]. The source code is released under the MIT License. All datasets used in this paper are published datasets available for download. Human DLPFCs is accessible within the SpatialLIBD [70] at http://spatial.libd.org/spatialLIBD. Human breast cancer and mouse brain tissue sections datasets are collected from the 10 $\times$ Genomics website at https://www.10xgenomics.com/resources/datasets. The mouse visual cortex STARmap data [71] is accessible at https://www.wangxiaolab.org/data-portal-1. The mouse brain cortex osmFISH data [72] is accessible at http://linnarssonlab.org/osmFISH. Slide-seqV2 dataset [73] is available at the Broad Institute Single Cell Portal at https://singlecell.broadinstitute.org/single_cell/study/SCP815. The processed Stereo-seq data [74] from mouse olfactory bulb tissue is accessible at https://github.com/JinmiaoChenLab/SEDR_analyses. The MERFISH data [75] collected from the mouse hypothalamic preoptic region is accessible at https://zhuang.harvard.edu/merfish.
