# Supplementary material for: MNMST: topology of cell networks leverages identification of spatial domains from spatial transcriptomics data
Source: Genome Biol. 2024 May 23;25:133. doi: 10.1186/s13059-024-03272-0 (PMC11112797; doi:10.1186/s13059-024-03272-0)
Supplement: Supplementary file 1 — Additional file 1: Supplementary information. Supplementary notes describing the detailed derivations of the MNMST algorithm, and Supplementary figures. [file 13059_2024_3272_MOESM1_ESM.pdf]

Supplementary Materials for

MNMST: Topology of cell networks leverages identification of spatial domains from spatial transcriptomics data

Yu Wang [1,2], Zaiyi Liu [3,4] and Xiaoke Ma [1,2*]

[1] School of Computer Science and Technology, Xidian University, No.2 South Taibai Road, Xi'an Shaanxi, 710071, China,

[2] Key Laboratory of Smart Human-Computer Interaction and Wearable Technology of Shaanxi Province, Xidian University, No.2 South Taibai Road, Xi'an Shaanxi, 710071, China,

[3] Department of Radiology, Guangdong Provincial People's Hospital (Guangdong Academy of Medical Sciences), Southern Medical University, 106 Zhongshan Er Road, Guangzhou, 510080, China,

[4] Guangdong Provincial Key Laboratory of Artificial Intelligence in Medical Image Analysis and Application, Guangdong Provincial People's Hospital, Guangdong Academy of Medical Sciences, Guangzhou, 510080, China

Corresponding author. Email: xkma@xidian.edu.cn

Keywords: Spatial transcriptomic, Spatial domain, Network model, Joint learning, Topological structure, Integrative analysis

1. Supplementary materials of MNMST

1.1 Mathematical model for MNMST

1.1.1 Mathematical model for learning cell multi-layer networks

Here, we present the detailed description of the MNMST algorithm, which first constructs cell spatial neighbor graph $W^{[s]}$ with cell spatial location by using k-nearest neighbor algorithm (by following Squidpy [1] , the number of neighbors k is 6 for 10 × Genomics data, 8 for Stereo-seq and Slide-seq V2 data, and 15 for imaging-based platforms). In the cell spatial neighbor network $W^{[s]}$, weights on edges measure the similarity of cell pairs in spatial. Recently, evidence demonstrates that high-order topological structure is more precise to characterize patterns [2] . By following Ref. [3] , MNMST takes pointwise mutual information matrix (PMI) to construct cell spatial network $M^{[s]}$, and each element is defined as

$$m_{ij}^{[s]} = log\frac{w_{ij}^{[s]} \sum_l d_l}{d_i d_j} - log\,\kappa \tag{1}$$

where d_l is the degree of l-th cell in network $W^{[s]}$, and κ is the number of non-negative samples (default set to 1).

On the construction of cell expression network $W^{[e]}$, given the low-dimensional cell expression feature matrix X, it is difficult to select an appropriate manner to precisely characterize distances of cells. MNMST addresses this issue by employing sparse self-representation learning (SRL) [8] under the assumption that cells are well represented by their neighbors. Thus, the objective function is formulated as

$$\min\left\|X - XW^{[e]}\right\|^2, \quad \text{s.t.}\, diag(W^{[e]}) = 0,$$

where $\|\cdot\|^2$is l_2−norm, and constraint ensures no self-loop in the learned cell expression network $W^{[e]}$. One limitation of the above equation is that the learned network is dense, i.e., clique, where any pair of cells exist an edge. Actually, networks of cells are sparse. Thus, we ensure sparsity of $W^{[e]}$ with l_1-norm constraint, i.e.,

$$\min\left\|X - XW^{[e]}\right\|^2 + \alpha\left\|W^{[e]}\right\|_1, \quad \text{s.t.}\, diag(W^{[e]}) = 0. \tag{2}$$

Moreover, we also expect cells proximal in the original feature space to have similar reconstructive coefficients. In other words, we expect the cell expression network $W^{[e]}$ to preserve the intrinsic geometrical structure of cell expression matrix X. The locality preserving projections

[9] shows that preservation of topological structure in X can be expressed as trace optimization. i.e.,

$$\min_{W^{[e]}} \sum_{ij} \left\| W_{.i}^{[e]} - W_{.j}^{[e]} \right\| w_{ij} = Tr(W^{[e]} L W^{[e]\prime}) \tag{3}$$

where $Tr(\cdot)$ denotes matrix trace, L is the Laplacian matrix of graph $X'X$, and $W_{.i}^{[e]}$ is the i-th column of matrix $W^{[e]}$. By combing Eq. (2) and Eq. (3), the objective function for cell expression network construction is formulated as follows:

$$\mathscr{L}(W^{[e]}) = \left\|X - XW^{[e]}\right\|^2 + \alpha\left\|W^{[e]}\right\|_1 + \beta Tr(W^{[e]} L W^{[e]'}), \tag{4}$$

$$\text{s.t. } W^{[e]} \geq 0, diag(W^{[e]}) = 0.$$

where α and β are regularization parameters that balance the relative importance of the reconstruction error term, sparse term, and trace optimization term, respectively.

1.1.2 Mathematical model for learning spatial and transcriptional feature of cells

MNMST learns the spatial and transcriptional feature of cells by exploiting cell multi-layer networks. To obtain the relations between different layers of multi-layer networks, MNMST adopts joint nonnegative matrix factorization [4] to simultaneously decompose the cell multi-layer network, i.e.,

$$\min \| W^{[e]} - BF^{[e]} \|^2 + \| M^{[s]} - BF^{[s]} \|^2$$

$$s.t.\ B \geq 0, F^{[e]} \geq 0, F^{[s]} \geq 0, \tag{5}$$

where matrix B is the common basis matrix with the shared features across different layers of the multi-layer network.

Then, MNSMT aims to learn the structural relations of cells, i.e., affinity graph, by exploiting the shared features of cells. Analogously, MNMST also adopts SRL to learn the affinity graph, which is formulated as:

$$B' = B'Z + E, \tag{6}$$

where Z denotes the coefficient matrix, and E is the error term. To capture the global structure of the network, a low-rank constraint is imposed on the coefficient matrix Z. Eq. (6) is transformed into the regularized rank minimization problem as:

$$\min\ rank(Z) + \|E\|_l \tag{7}$$

The Eq. (7) is difficult to solve because of the low-rank constraint. Following [6] , MNMST adopts nuclear norm as an alternative. In this way, Eq. (7) is re-formulated as:

$$min \ \|Z\|_* + \|E\|_{2,1} \tag{8}$$

where $\|\cdot\|_*$ and $\|\cdot\|_{2,1}$ are the nuclear norm and $l_{2,1}$-norm, i.e., $\|E\|_{2,1} = \sum_{j=1}^{n}\sqrt{\sum_{i=1}^{d} E_{ij}^2}$.

By combing Eq. (5) and Eq. (8), the objective of affinity graph learning is:

$$\|W^{[e]} - BF^{[e]}\|^2 + \|M^{[s]} - BF^{[s]}\|^2 + \gamma\|Z\|_* + \lambda\|E\|_{2,1}$$

$$s.t. \ \ B \geq 0, F^{[e]} \geq 0, F^{[e]} \geq 0, B' = B'Z + E \tag{9}$$

where γ and λ are parameters controlling the relative importance of the low-rank constraint and the error term.

1.2 Optimization of cell expression network construction

Since the objective function in Eq. (4) consists of a differentiable portion $\left\|X - XW^{[e]}\right\|^2 + Tr(W^{[e]}LW^{[e]'})$ and a non-differentiable portion $\left\|W^{[e]}\right\|_1$, we use an alternating optimization approach to update one variable by fixing the others, and algorithm continues until the convergence criterion is met.

By introducing auxiliary variables J_1, Eq. (4) is transformed into the equivalent form, i.e.,

$$\mathscr{L}(W^{[e]}, J_1) = \left\|X - XW^{[e]}\right\|^2 + \alpha\|J_1\|_1 + \beta Tr(W^{[e]}LW^{[e]'}),$$

$$\text{s.t. } W^{[e]} \geq 0, diag(W^{[e]}) = 0, W^{[e]} = J_1. \tag{10}$$

The augmented Lagrange function of Eq. (10) is defined as

$$\mathscr{L}(W^{[e]}, J_1; T_1) = \left\|X - XW^{[e]}\right\|^2 + \alpha\|J_1\|_1 + \beta Tr\left(W^{[e]}LW^{[e]'}\right)$$

$$+ < T_1, W^{[e]} - J_1 > + \mu_1\left\|W^{[e]} - J_1\right\|^2, \tag{11}$$

where $\mu_1 > 0$ is the positive penalty scalar, T_1 is the Lagrange multiplier, and $<\cdot,\cdot>$ denotes the inner product of matrices.

According to ADMM (Alternating Direction Method of Multipliers) algorithm [5] , problem in Eq. (11) can be solved with the following sub-problems, i.e.,

$$\begin{cases} W^{[e]} \leftarrow \underset{W^{[e]}}{argmin}\left\|X - XW^{[e]}\right\|^2 + \beta Tr(W^{[e]}LW^{[e]'}) + \mu_1\left\|W^{[e]} - J_1 + \frac{T_1}{\mu_1}\right\|^2 \\ J_1 \leftarrow \underset{J_1}{argmin}\mu_1\left\|W^{[e]} - J_1 + \frac{T_1}{\mu_1}\right\|^2 + \alpha\|J_1\|_1 \\ T_1 = T_1 + \mu_1(W^{[e]} - J_1) \end{cases} \tag{12}$$

Sub-problem $W^{[e]}$ can be approximated with Eq.(12). By setting the partial derivative $\frac{\partial\mathscr{L}}{\partial W^{[e]}}$=0, the update rule for $W^{[e]}$ is formulated as

$$W^{[e]} = W^{[e]} \odot \frac{X^T X + \mu J_1 - T_1 + \beta W}{X^T X W^{[e]} + \beta D W^{[e]} + \mu_1 W^{[e]}} \ , \tag{13}$$

where e represents element-wise multiplication.

By fixing $W^{[e]}$, sub-problem J_1 can be approximated by the above iteration, then

$$J_1 = \underset{J_1}{argmin}\, \mu \left\| W^{[e]} - J_1 + \frac{T_1}{\mu_1} \right\|^2 + \alpha \|J_1\|_1 = Soft_{\alpha/\mu}(J_1 - \frac{T_1}{\mu}). \tag{14}$$

where $Soft$ is a soft threshold function defined as

$$Soft_{\varepsilon}[x] = \begin{cases} x + \varepsilon, & if\ x > \varepsilon, \\ x - \varepsilon, & if\ x < -\varepsilon, \\ 0, & otherwise. \end{cases}$$

These equations lead to the following updating rules as

$$W^{[e]} \leftarrow W^{[e]} \odot \frac{X^T X + \mu J_1 - T_1 + \beta W}{X^T X W^{[e]} + \beta D W^{[e]} + \mu_1 W^{[e]}},$$

$$T_1 \leftarrow T_1 + \mu_1 (W^{[e]} - J_1). \tag{15}$$

1.3 Optimization of learning cell spatial and transcriptional features

The problem in Eq. (9) is also solved by the alternating direction method of multipliers. By introducing the auxiliary variables J_2 and H, Eq. (9) is equivalent to the following problem, i.e.,

$$min \| W^{[e]} - BF^{[e]} \|^2 + \|M^{[s]} - BF^{[s]}\|^2 + \gamma \|J_2\|_* + \lambda \|E\|_{2,1}$$

$$\text{s.t. } B \geq 0, F^{[e]} \geq 0, F^{[s]} \geq 0, H = HZ + E, H = B', Z = J_2. \tag{16}$$

The augmented Lagrange function of Eq. (16) is formulated as

$$\mathscr{L}(B, F^{[e]}, F^{[s]}, H, E, Z, J_2) = \left\|W^{[e]} - BF^{[e]}\right\|^2 + \left\|M^{[s]} - BF^{[s]}\right\|^2 + \gamma \|J_2\|_* + \lambda \|E\|_{2,1}$$

$$+ < T_2, H - HZ - E > + \mu_2 \|H - HZ - E\|^2$$

$$+ < T_3, H - B' > + \mu_2 \|H - B'\|^2$$

$$+ < T_4, Z - J_2 > + \mu_2 \|Z - J_2\| \tag{17}$$

where $\mu_2 > 0$ is the positive penalty scalar, $T_2, T_3, and\ T_4$ are the Lagrange multipliers, $<\cdot,\cdot>$ denotes the inner product of matrices. MNMST updates one variable while fixing the others, which continues until the convergence criterion is reached. We exploit this special structure and optimize the objective by alternatingly updating the variable sets B, $F^{[e]}$, $F^{[s]}$, E, Z and H.

According to ADMM (Alternating Direction Method of Multipliers) algorithm [5] , problem in Eq. (17) can be solved with the following sub-problems, i.e.,

$$\begin{cases} F^{[e]} \leftarrow \underset{F^{[e]}}{argmin} \left\| W^{[e]} - BF^{[e]} \right\|^2 \\ F^{[s]} \leftarrow \underset{F^{[s]}}{argmin} \left\| M^{[s]} - BF^{[s]} \right\|^2 \\ B \leftarrow \underset{B}{argmin} \left\| W^{[e]} - BF^{[e]} \right\|^2 + \left\| M^{[s]} - BF^{[s]} \right\|^2 + \mu_2 \left\| H - B' + \frac{T_3}{\mu_2} \right\|^2 \\ H \leftarrow \underset{H}{argmin} \mu_2 \left\| H - HZ - E + \frac{T_2}{\mu_2} \right\|^2 + \mu_2 \left\| H - B' + \frac{T_3}{\mu_2} \right\|^2 \\ Z \leftarrow \underset{Z}{argmin} \mu_2 \left\| H - HZ - E + \frac{T_2}{\mu_2} \right\|^2 + \mu_2 \left\| Z - J_2 + \frac{T_4}{\mu_2} \right\|^2 \\ E \leftarrow \underset{E}{argmin} \mu_2 ||H - HZ - E + \frac{T_2}{\mu_2}||^2 + \lambda ||E||_{2,1} \\ J_2 \leftarrow \underset{J_2}{argmin} \gamma ||J_2||_* + \mu_2 ||Z - J_2 + \frac{T_4}{\mu_2}||^2 \\ T_2 \leftarrow T_2 + \mu_2 (H - HZ - E) \\ T_3 \leftarrow T_3 + \mu_2 (H - B') \\ T_4 \leftarrow T_4 + \mu_2 (Z - J_2) \end{cases} \quad (18)$$

Specifically, MNMST first optimizes $F^{[e]}$ and $F^{[s]}$ by fixing the other variables. Sub-problem $F^{[e]}$ and $F^{[s]}$ can be approximated by the above iterations. By setting the partial derivatives $\frac{\partial \mathcal{L}}{\partial F^{[e]}}$ and $\frac{\partial \mathcal{L}}{\partial F^{[s]}}$ to 0, the update rules for $F^{[e]}$ and $F^{[s]}$ are formulated as

$$F^{[e]} = F^{[e]} \odot \frac{B^T W^{[e]}}{B^T B F^{[e]}},$$

$$F^{[s]} = F^{[s]} \odot \frac{B^T M^{[s]}}{B^T B F^{[s]}}. \quad (19)$$

Sub-problem B: by setting the partial derivative $\frac{\partial \mathcal{L}}{\partial B}$ to 0, the update rule for B is deduced as

$$B = B \odot \frac{W^{[e]} F^{[e]\prime} + M^{[s]} F^{[s]\prime} + \mu_2 H' + T_3'}{B F^{[e]} F^{[e]\prime} + B F^{[s]} F^{[s]\prime} + \mu_2 B}. \quad (20)$$

Analogously, the update rule for H and B is formulated as

$$H = H \odot \frac{(E - \frac{T_2}{\mu_2})(I - Z)^T + B^T - \frac{T_3}{\mu_2}}{H(I - Z)(I - Z)^T + H}, \quad (21)$$

$$Z = Z \odot \frac{H^T H - H^T E + J_2 + \frac{H^T T_2 - T_4}{\mu_2}}{H^T H Z + Z}. \quad (22)$$

Sub-problem E: let $A = H - HZ + T_2/\mu_2$, the optimization of E is reformulated as

$$E \leftarrow \underset{E}{argmin}\, \mu_2 ||E - A||^2 + \lambda ||E||_{2,1} \quad (23)$$

The above problem can be solved as Eq. (24) according to Ref.[6] as

$$E_{:,j} = \begin{cases} \frac{||A_{:,j}||_2 - \frac{\lambda}{\mu}}{||A_{:,j}||_2} A_{:,j}, & ||A_{:,j}||_2 \geq \frac{\lambda}{\mu} \\ 0, & otherwise. \end{cases} \quad (24)$$

Sub-problem J_2 can be approximated as

$$J_2 \leftarrow \underset{J_2}{argmin} \frac{\gamma}{\mu_2} ||J_2||_* + \left\| J_2 - \left(Z + \frac{T_4}{\mu_2} \right) \right\|^2, \quad (25)$$

which can be solved by using a singular value thresholding operator according to Ref.[7] .

1.4 Identification of spatial domains

After obtaining the matrix Z, the affinity graph is constructed as $(|Z| + |Z'|)/2$ since Z is unnecessarily symmetric. The Leiden algorithm [11] is deployed on the affinity graph to obtain spatial domains. The procedure of MNMST is described in Algorithm 1.

1.5 Algorithm analysis

On the time complexity issue, time for updating Z, H and J_2 requires $O(n^2 l)$, where l is the number of iterations. Time for updating $W^{[e]}$ and J_1 is $O(n^2 m)$, where m is the number of dimensions. Time for $F^{[e]}$, $F^{[s]}$and B is $O(n^2 dl)$. The complexity for updating E is $O(n^2)$. Therefore, the time complexity of MNMST is $O(n^2 dl)$.

On the space complexity issue, MNMST constructs the cell spatial and expression network, requiring space $O(2n^2)$. The space for feature matrix is $O(nd)$, and space for cell affinity graph is $O(n^2)$. Therefore, the overall space complexity of MNMST is $O(n^2)$.

1.6 Running time of MNMST and acceleration strategy

For a fair comparison, all algorithms are executed on the same platform with Intel i9 CPU, 64GB RAM, and an NVIDIA RTX4090.

On the 10×Genomics data, we independently execute each algorithm for each slide of DLPFC data [12] , where the distributions of running time and space of algorithms are summarized in Fig. S4 A1 and A2, respectively. From Fig. S4 A1, it is easy to conclude that Giotto [13] and BayesSpace [14] are slower than stLearn [15] and MNMST, and SCANPY [16] , SpaGCN [17] and DeepST [18] are faster than others. Specifically, running time of MNMST is 4.3±2.5 minutes, while that is 10.6 ±2.9 (Giotto), 6.3±2.2 (BayesSpace), 3.6±0.4 (stLearn), 2.2±0.4 (DeepST), 1.4±0.3 (SpaGCN), 0.6±0.1 (SEDR) and 0.1±0.0 (SCANPY), respectively. Fig. S4 A2 illustrates distributions of spaces for various algorithms for DLPFC data, where space complexity of Gitto, stLearn and DeepST much higher than others. In details, space of MNMST is 3.6±0.1 Gigabytes, while that is 5.5±0.4 (Giotto), 1.2±0.1 (BayesSpace), 8.5±1.5 (stLearn), 9.3±0.7 (DeepST), 1.7±0.1 (SpaGCN), 1.9±0.1 (SEDR) and 1.5±0.2 (SCANPY), respectively.

From Fig. S4 A1 and A2, it is easy to assert that: First, Gitto and stLearn are time- and space-consuming, hampering the applications of identification of spatial domains in ST data. And, DeepST reduces running time by sacrificing space, whereas BayesSpace reduces space complexity by sacrificing running time. Second, SCANPY, SEDR and SpaGCN are efficient in terms of running time and space, whereas performance of these is undesirable. In other words, these algorithms improve efficiency by sacrificing accuracy of algorithms. Third, MNMST reaches a good tradeoff between space and running time. Furthermore, it achieves the best performance on the identification of spatial domains, demonstrating that MNMST provides an excellent alternative for current algorithms.

On the MERFISH data [19] , we validate efficiency of algorithms by increasing the number of spots/cells from 1,000 to 30,000. Since SEDR、SpaGCN, and DeepST employ graph convolutional network (GCN), and DeepST is superior to them in terms of accuracy. Therefore, only SCANPY, DeepST and MNMST are selected for a comparison. Fig. S4 B1 and B2 describe the running time and space of algorithms with various sizes of data respectively, where missing bar represents failure of algorithms to address the corresponding data. When the number of spots is less than or equal to 20,000, MNMST is worse than DeepST on both space and time. However, MNMST addresses ST data with more than 20,000 spots that DeepST fails to handle. The reason why DeepST fails to address large-scale ST data is that it employs deep learning to obtain features of spots/cells, where the number of parameters for learning exponentially increases, resulting in the expensive time complexity.

1.7 Parameter effect for spatial transcriptomics data generated with various platforms

By applying MNMST to spatial transcriptomics data generated by different platform, we investigate how ARI of MNMST changes as values of parameters vary as shown in Fig. S5, where panel A is for 10 × Genomics, and B for osmFISH [20] , and C for STARmap [21] data, respectively. Fig. S5 A depicts how ARI of MNMST changes as parameters (γ, λ) increase from 0.1 to 80 for DLPFC data, where MNMST is quit stable. In details, as parameter γ increases from 0.1 to 1, ARI of MNMST dramatically improves. And, MNMST is quit stable when $\gamma \in$ [1, 80].

Moreover, as parameter $\lambda \in [0.1, 20]$, MNMST achieves the best performance. However, it performance dramatically decreases when $\lambda > 20$.

There are several possible reasons to explain this tendency. When γ is too small, the contribution of structure constraint for cell affinity graph is limited, thereby reducing performance of MNMST. Increasing value of parameter γ ensures structure of spatial domains in affinity graph, enhancing performance of MNMST. Furthermore, when parameter λ is too large, MNMST over-emphasizes sparsity of cell affinity graph, resulting in unbalance between structure of cell affinity network and spatial domains.

Fig. S5 B depicts how ARI of MNMST changes as parameters (γ, λ) increase from 0.1 to 80 for MERFISH data, where MNMST achieves the best performance if $\lambda \in [10,30]$, and $\gamma \in [20, 80]$. Furthermore, Fig. S5 C depicts how ARI of MNMST changes as parameters (γ, λ) increase from 0.1 to 80 for STARmap data, where MNMST obtains the optimal performance as $\lambda \in [40,80]$, and $\gamma \in [1, 40]$. Careful comparison among panels of Fig. S5 demonstrates that parameters of MNMST are highly associated with spatial transcriptomics data because different platforms generate data with unique intrinsic structures.

Parameters of MNMST differ for various types of spatial transcriptomics data largely due to the intrinsic structure of data, and we suggest $\lambda \in [1,80]$, and $\gamma \in [1, 20]$ for 10 × Genomics data, $\lambda \in [10,30]$, and $\gamma \in [20, 80]$ for MERFISH data, and $\lambda \in [40,80]$, and $\gamma \in [1, 40]$, respectively.

Algorithm 1: The MNMST Algorithm

Input:

(X, A): Gene expression X and spatial information A;

d: Dimensional of common subspace;

λ, γ: Regularization parameters;

Output:

$\{C_i\}_{i=1}^{c}$: Clusters.

Part I: Cell Multi-layer network construction

1. Construct the cell topology network by using spatial proximity and morphological similarity;
2. Construct the cell expression network with Eq. (4);

Part II: Initialization

3. Initialize $J_2, F^{[e]}, F^{[s]}, B, Z, H, E$;

Part III: Optimization

4. Update $F^{[e]}, F^{[s]}$ according to Eq. (19);
5. Update B according to Eq. (20);
6. Update H according to Eq. (21);
7. Update Z according to Eq. (22);
8. Update E according to Eq. (24);
9. Update J_2 according to Eq. (25);
10. Go to step 4 until convergence;
11. Let $Z = \frac{|Z|+|Z|^T}{2}$;
12. Identify spatial domains with Leiden algorithm based on Z;

return $\{C_i\}_{i=1}^{c}$.

2. Supplementary Figures

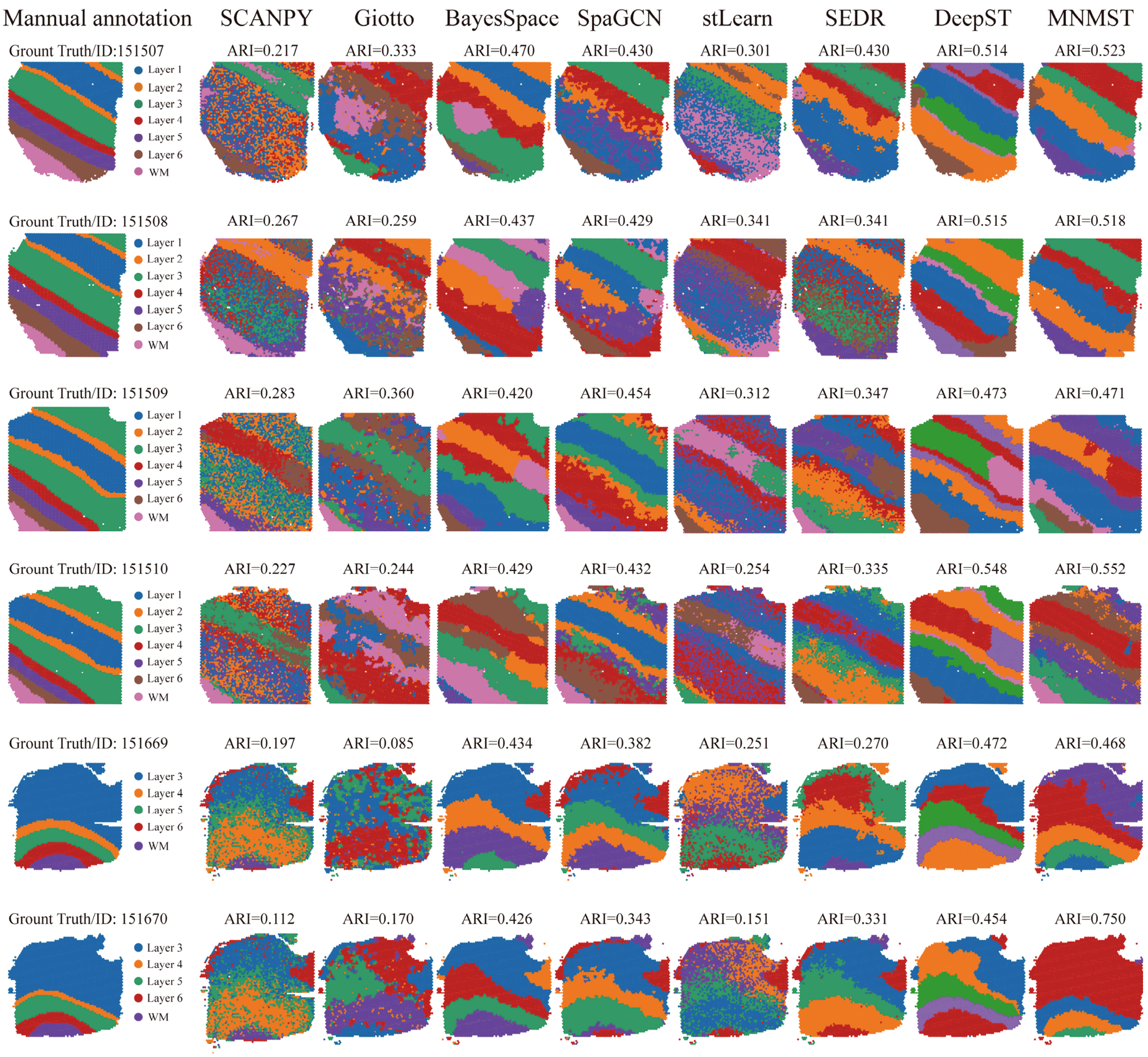

Fig. S1 Performance of various algorithms for spatial domain identification on Annotated dorsolateral prefrontal cortex (DLPFC, http://spatial.libd.org/spatialLIBD) data (151507, 151508, 151509, 151510, 151669, 151670), where ground truth spots are mapped on their spatial location, divided into various cortical layers (L1-L6 or L3-L6) and white matter (WM) layer, and each column corresponds to performance of an algorithm for various slices in terms of ARI.

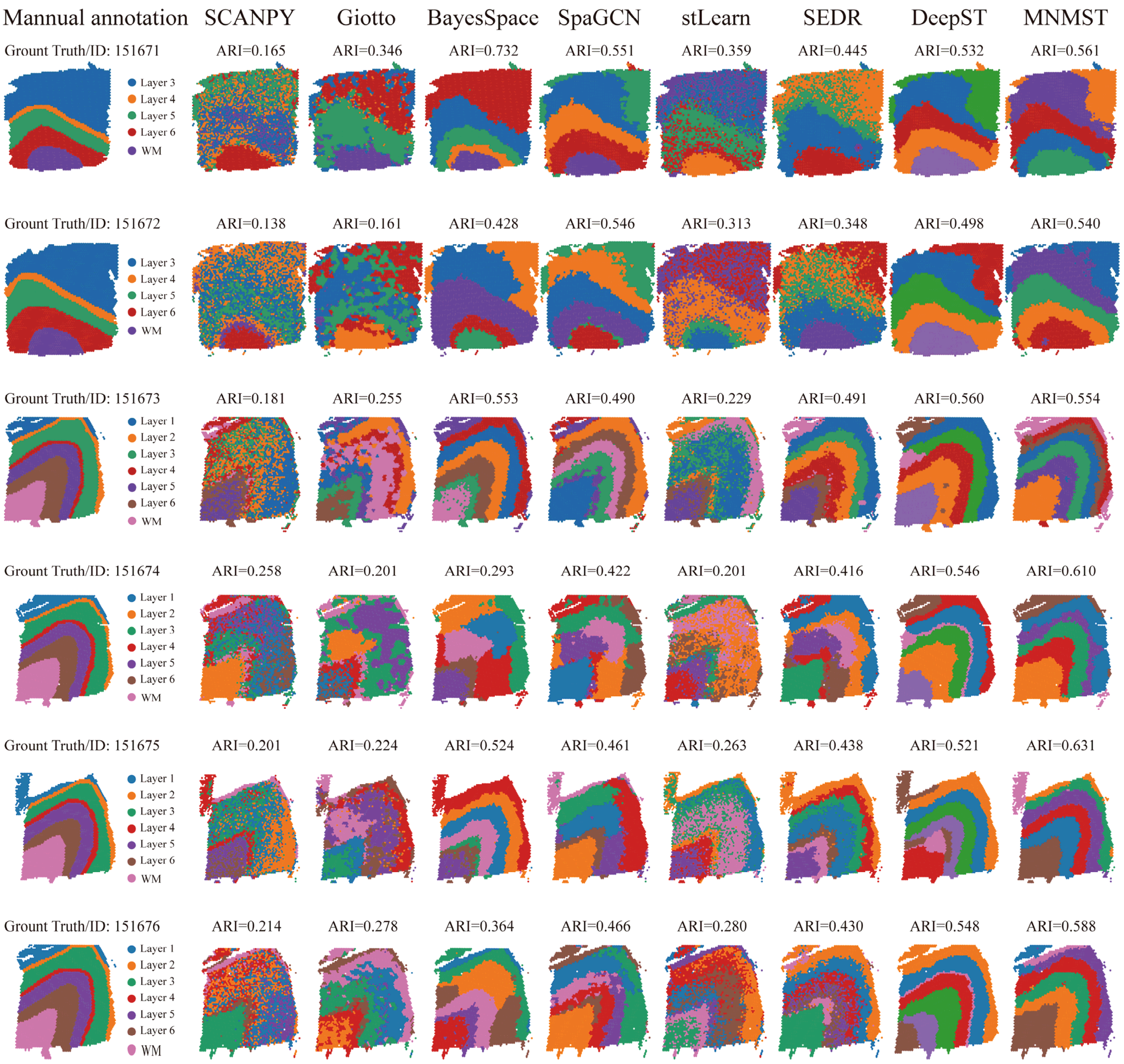

Fig. S2 Performance of various algorithms for spatial domain identification on Annotated dorsolateral prefrontal cortex (DLPFC, http://spatial.libd.org/spatialLIBD) data (151671, 151672, 151673, 151674, 151675, 151676), where ground truth spots are mapped on their spatial location, divided into various cortical layers (L1-L6 or L3-L6) and white matter (WM) layer, and each column corresponds to performance of an algorithm for various slices in terms of ARI.

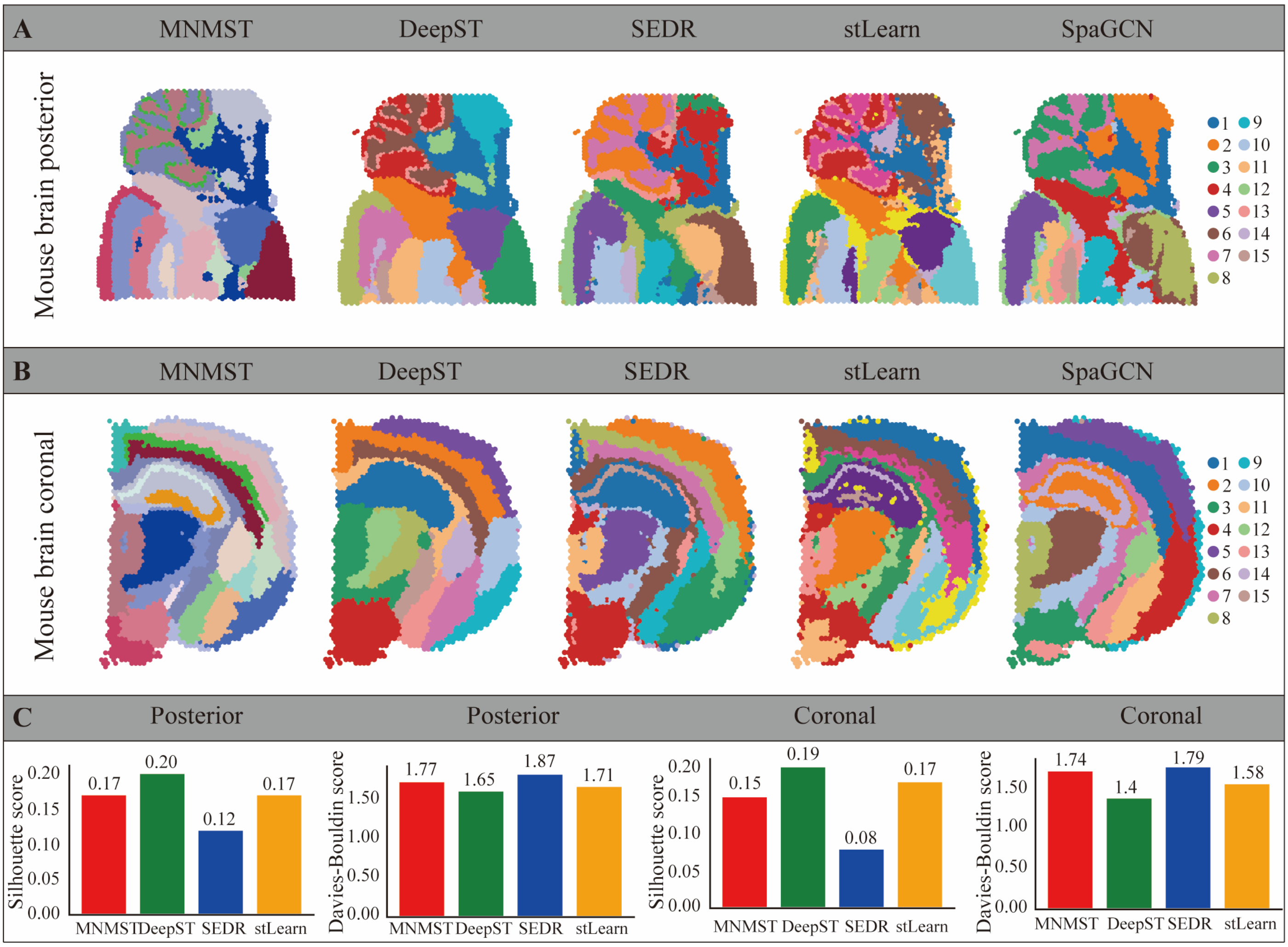

Fig. S3 Spatial domain identification for mouse brain tissue. (A) and (B) Spatial domains identified by MNMST, DeepST, SEDR, stLearn, and SpaGCN in mouse brain posterior and coronal, respectively. (C) Histograms of Silhouette Coefficient (SC) and Davies-Bouldin (DB) scores for spatial domains identified by various algorithms for mouse brain posterior and coronal data.

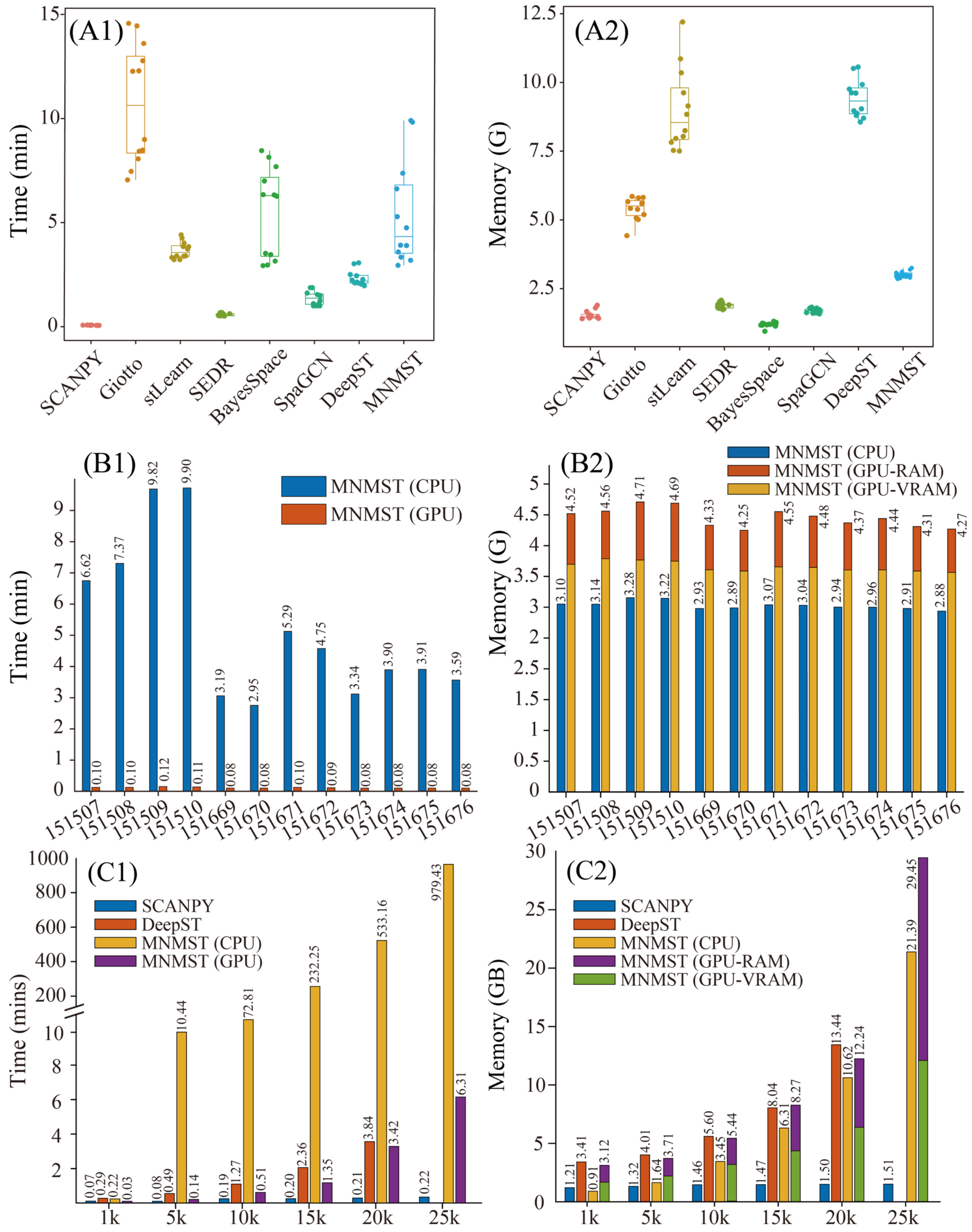

Fig. S4 Running time and space of algorithms for different spatial transcriptomics data: Distributions of running time (minutes) (A1) and space (Gigabyte) (A2) of algorithms on the DLPFC dataset, Running time (minutes) (B1) and space (Gigabyte) (B2) of MNMST with CPU and GPU on the DLPFC dataset, and Running time (minutes) (C1) and space (Gigabyte) (C2) of different algorithms on MERFISH data, respectively, where missing bar represents failure of algorithms to address the corresponding data.

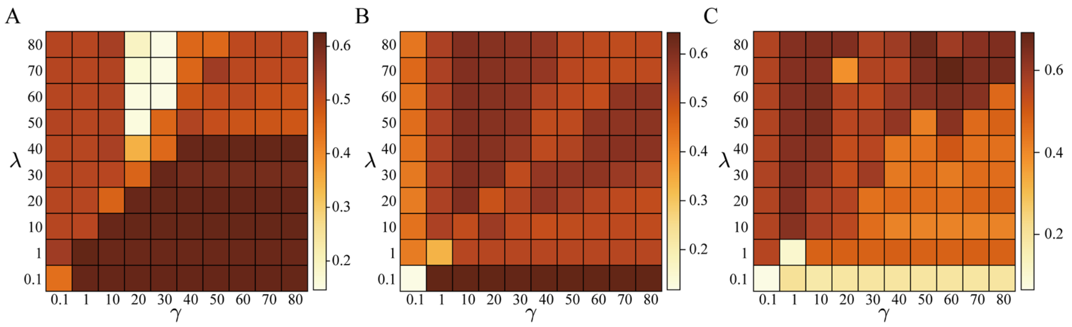

Fig. S5 Parameter effects of MNMST for various spatial transcriptomics data: (A) ARI vs parameter (γ, λ) for 10 × Genomics data, (B) ARI vs parameter (γ, λ) for osmFISH data, and (C) ARI vs parameter (γ, λ) for STARmap data.

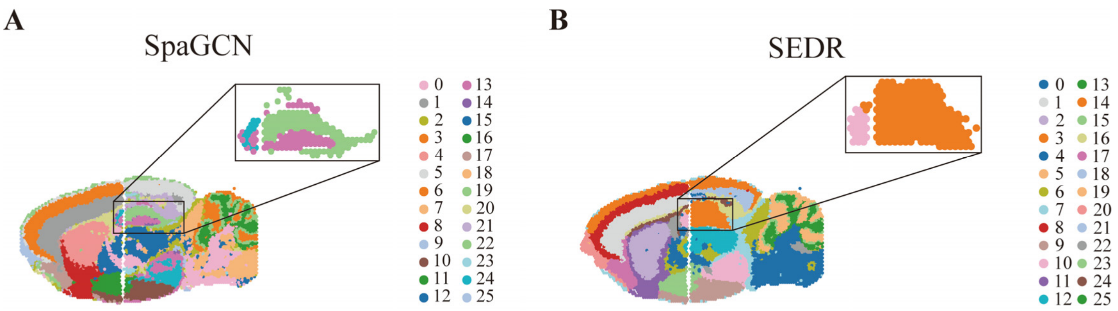

Fig. S6 Spatial domains identified by various algorithms in horizontally aligned mouse brain samples: (A) SEDR, and (B) SpaGCN, respectively.

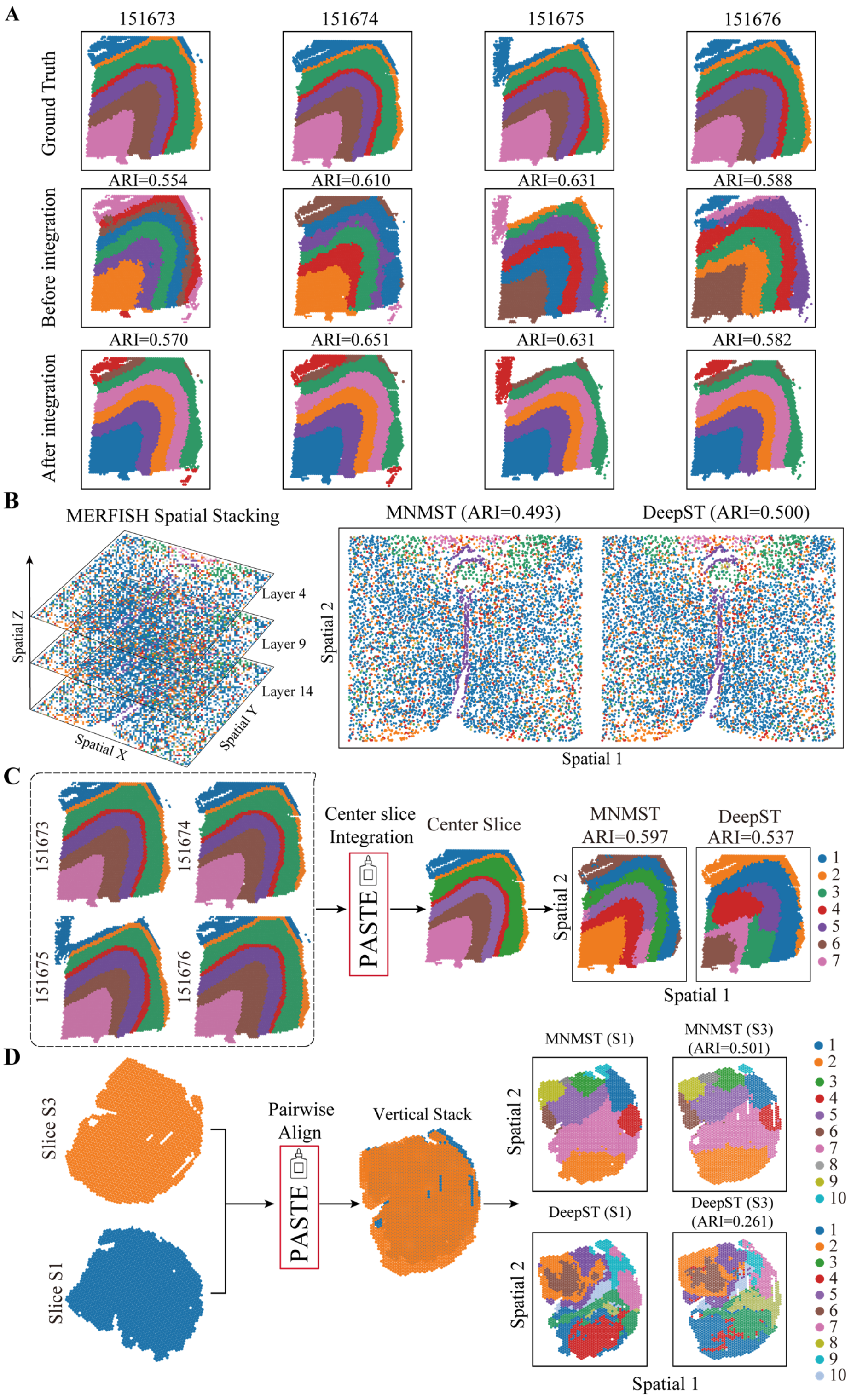

Fig. S7 Performance of MNMST on vertical integration of spatial transcriptomics data. (A)Spatial domains identified by MNMST for slices 151673, 151674, 151675, and 151676 in DLPFC data before and after integration. (B) Performance of DeepST and MNMST on the 3D coordinates of MERFISH data. (C) Performance of DeepST and MNMST on the center slice generated by PASTE. (D) Performance of MNMST and DeepST for mouse brain slice S3 in terms of ARI, where the manual annotation of slide S3 as ground truth.

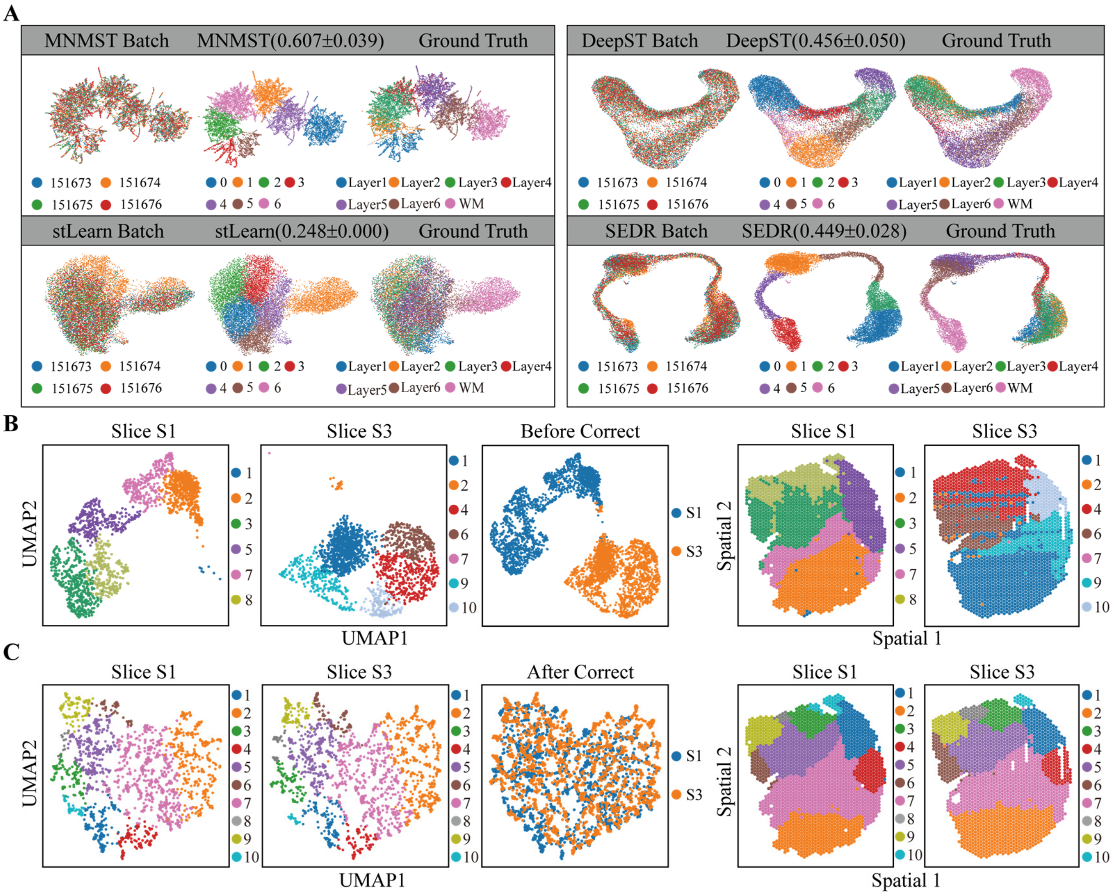

Fig. S8 Performance of MNMST on removing batch effects of spatial transcriptomics data. (A) UMAP plots of spatial integrated algorithms, including MNMST, DeepST, stLearn and SEDR, for slices 151673, 151674, 151675, and 151676 in DLPFC data. They represent batches, recognition spatial domains, and ground truth labels, respectively. (B) UMAP plots of the S1, S3 and stacked slice before integration. UMAP plots of spatial domains identified by MNMST for slice S1 and S3 in mouse breast cancer data. (C) UMAP plots of the S1, S2 and stacked slice after integration. UMAP plots of spatial domains identified by MNMST for slice S1 and S3 in mouse breast cancer data.

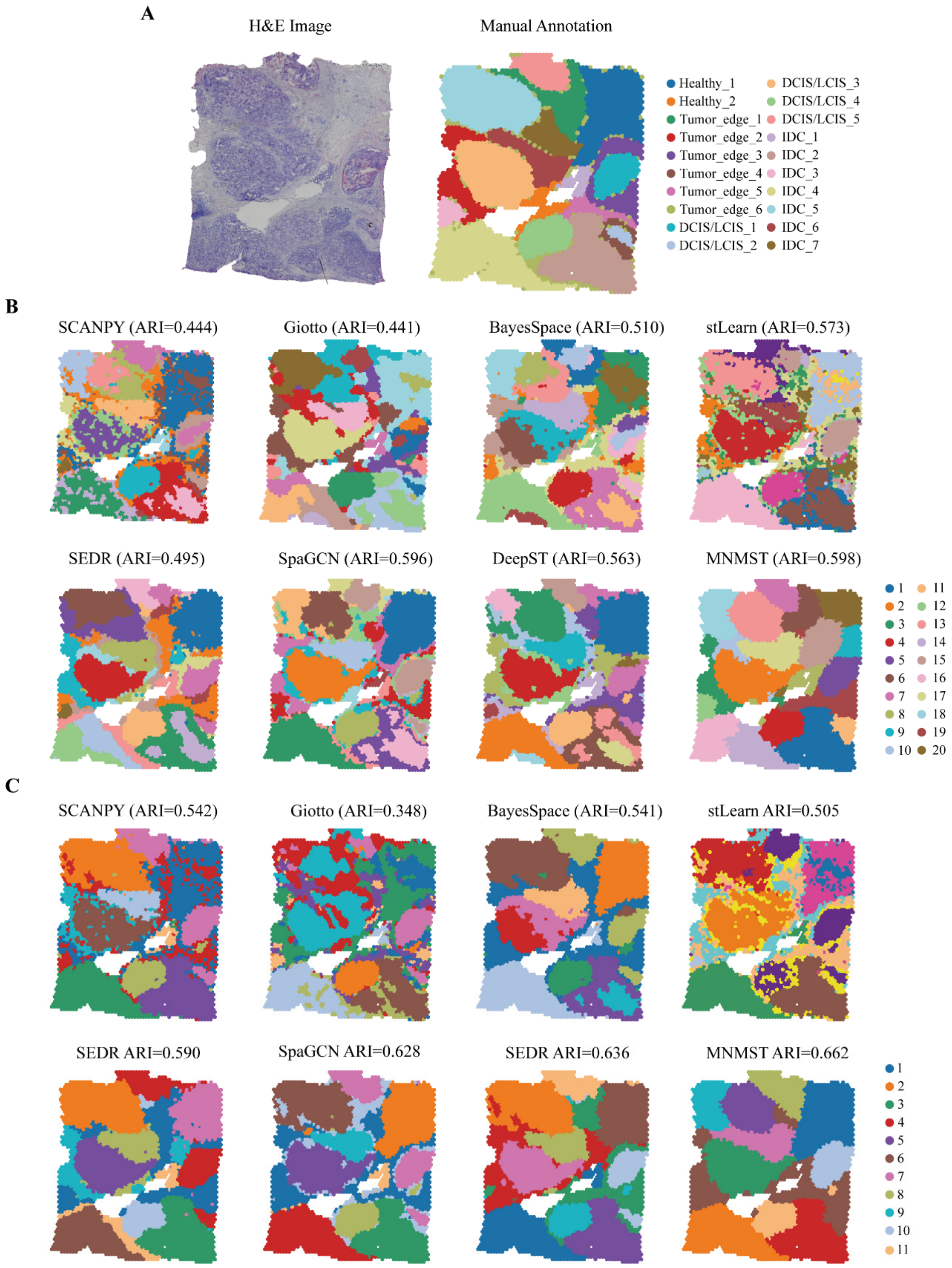

Fig. S9 Performance of various algorithms for human breast cancer data. (**A**) H&E image (left) and manual annotation (right). (**B**) Comparison of spatial domains identified by SCANPY, Giotto, BayesSpace, stLearn, SEDR, SpaGCN, DeepST, and MNMST on the human breast cancer data, and visualization of identified spatial domains (domains=20). (**C**) Visualization of spatial domains identified by different methods with domains=11.

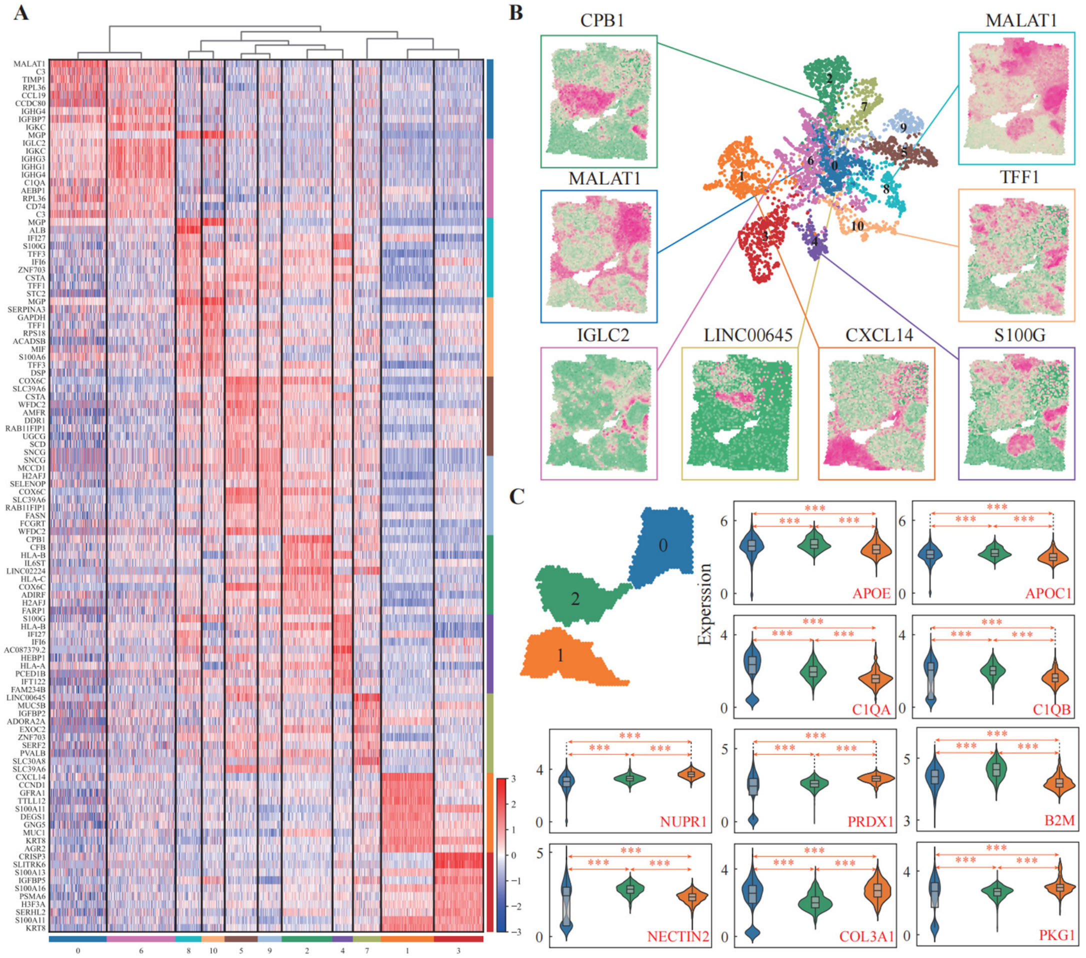

Fig. S10 Differential expressed genes of spatial domains identified by MNMST. (**A**) Heatmap of DEGs for 11 identified spatial domains on the breast cancer data. (**B**) UMAP visualization of domains and a typical DEG in each domain. (**C**) Violin plots of DEGs among Healthy (blue) vs IDC (orange), and DCIS/LCIS (green), where significance is calculated with Wiscoxon sum-rank test (* denotes p<0.05, ** denotes p<0.01, and *** denotes p<0.001).

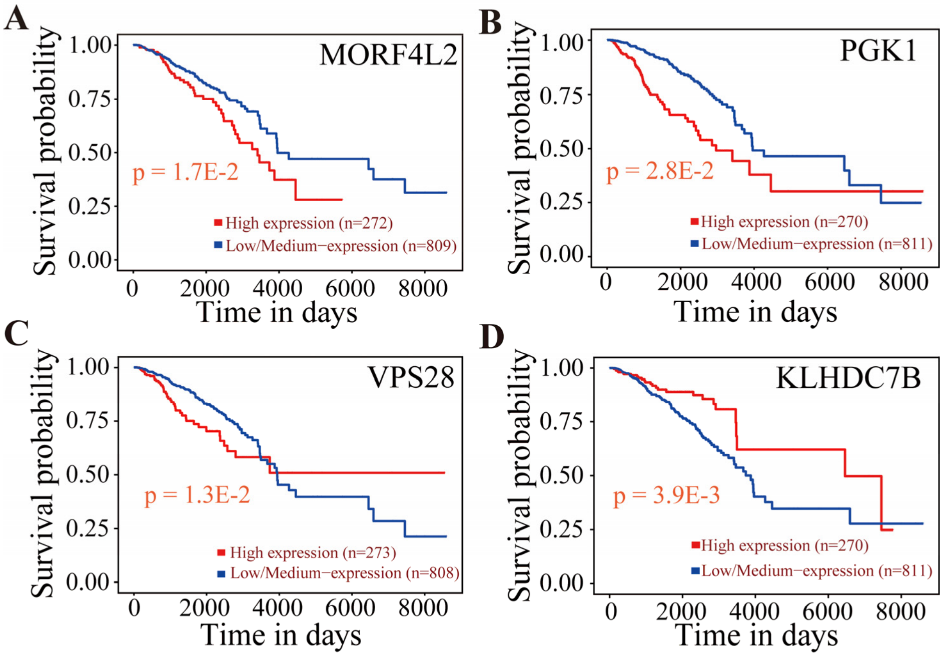

Fig. S11 Differential expressed genes of spatial domains identified by MNMST associated with survival time of patients using Kaplan-Meier survival analysis (log-rank for significance): (**A**) MORF4L2, (**B**) PGK1, (**C**) VPS28, and (**D**) KLHDC7B.

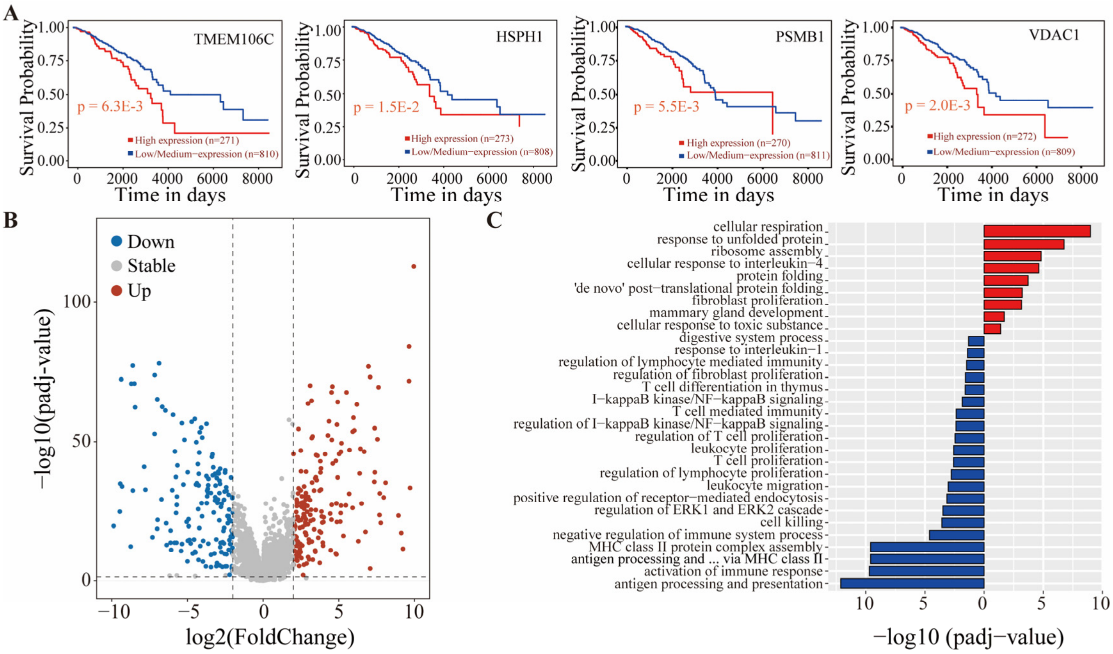

Fig. S12 Differential expression analysis between domain 1 and 0 on human breast data. **(A)** Spatial domain related DEGs predict survival time of patients with Kaplan-Meier survival analysis (log-rank test for significance). **(B)** Volcano plot of DEGs (*domains*=11), where x-axis denotes log2(Fold Chang), and y-axis represents -log10(padj-value). **(C)** Biological functions significantly enriched by up-regulated (red) and down-regulated (blue) DEGs, where x-axis denotes -log10(padj-value) (hypergeometric test for significance).

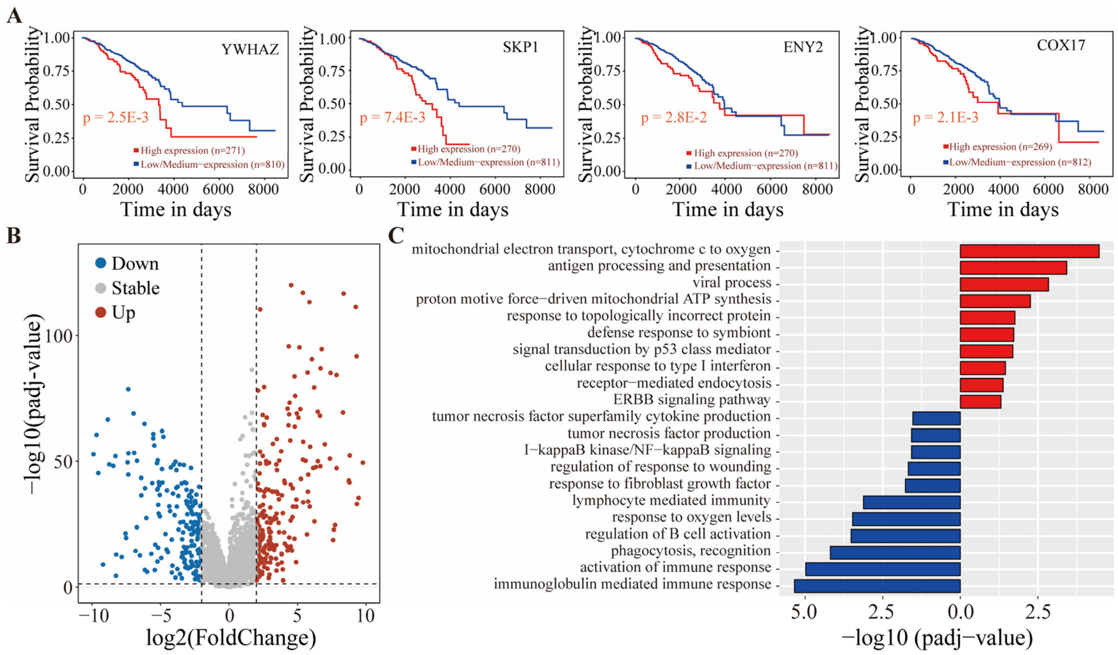

Fig. S13 Differential expression analysis between domain 2 and 0 on human breast data. **(A)** Spatial domain related DEGs predict survival time of patients with Kaplan-Meier survival analysis (log-rank test for significance). **(B)** Volcano plot of DEGs (*domains*=11), where x-axis denotes log2(Fold Chang), and y-axis represents -log10(padj-value). **(C)** Biological functions significantly enriched by up-regulated (red) and down-regulated (blue) DEGs, where x-axis denotes -log10(padj-value) (hypergeometric test for significance).

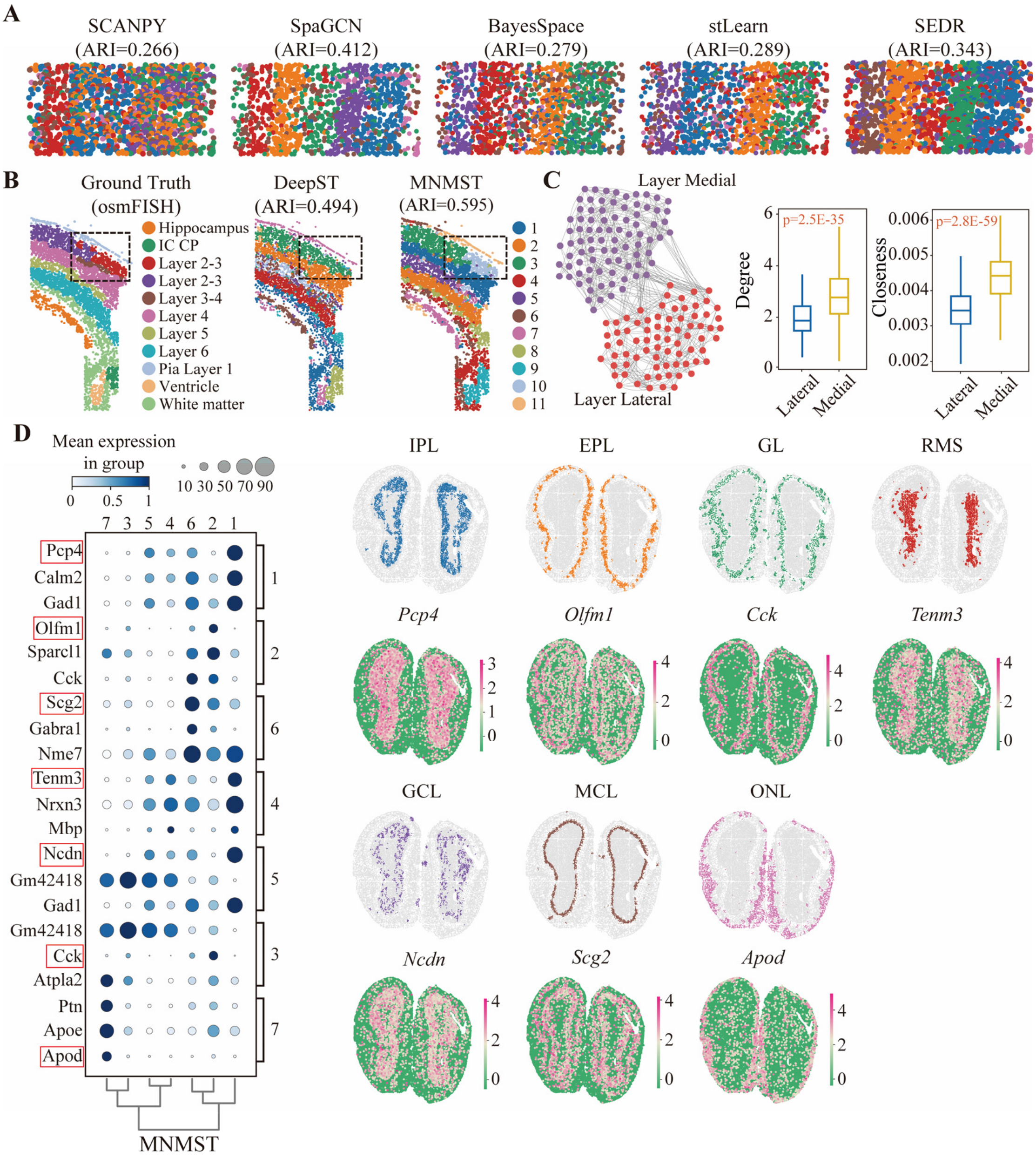

Fig. S14 Multi-layer network model works on data from various platforms. **(A)** Visualization of spatial domains identified by SCANPY, SpaGCN, BayesSpace, stLearn, SEDR, DeepST, and MNMST on STARmap data. **(B)** Visualization of osmFISH data (left). Spatial domains identified by DeepST (middle, ARI=0.494) and MNMST (right, ARI=0.595), where L1 and L2/3 domains are surrounded with dashed squares. **(C)** Topological structure of affinity graph for cells in nateral and medial layer, and distributions of degrees (middle) and closeness (right) between nateral and medial layer (Student's t-test for significance). **(D)** Dotplot of the top 3 DEGs of identified domains from mouse olfactory bulb data generated from Stereo-seq (left), and scatter plot of spatial clustering generated by MNMST, including genes *Pcp4*, *Olfm1*, *Cck*, *Tenm3*, *Ncdn*, *Scg2*, and *Apod* (right).

Reference

[1] Palla G, Spitzer H, Klein M, Fischer D, Schaar AC, Kuemmerle LB, et al. Squidpy: a scalable framework for spatial omics analysis. Nat Methods. 2022;19:171-178.

[2] Benson AR, Gleich DF, Leskovec J. Higher-order organization of complex networks. Science. 2016;353(6295):163–166.

[3] Li Y, Sha C, Huang X, Zhang Y. Community detection in attributed graphs: An embedding approach. In: Proceedings of the AAAI Conference on Artificial Intelligence. vol. 32; 2018.

[4] Ma X, Dong D, Wang Q. Community detection in multi-layer networks using joint nonnegative matrix factorization. IEEE Trans Knowl Data Eng. 2018;31:273–286.

[5] Boyd S, Parikh N, Chu E, Peleato B, Eckstein J, et al. Distributed optimization and statistical learning via the alternating direction method of multipliers. Found Trends Mach Learn. 2011;3:1–122.

[6] Liu G, Lin Z, Yan S, Sun J, Yu Y, Ma Y. Robust recovery of subspace structures by low-rank representation. IEEE transactions on pattern analysis and machine intelligence. 2012;35(1):171–184.

[7] Cai JF, Cand`es EJ, Shen Z. A singular value thresholding algorithm for matrix completion. SIAM Journal on optimization. 2010;20(4):1956–1982.

[8] Elhamifar E, Vidal R. Sparse subspace clustering: Algorithm, theory, and applications. IEEE transactions on pattern analysis and machine intelligence. 2013;35(11):2765–2781.

[9] Fang X, Xu Y, Li X, Fan Z, Liu H, Chen Y. Locality and similarity preserving embedding for feature selection. Neurocomputing. 2014;128:304–315.

[10] Zeira R, Land M, Strzalkowski A, Raphael BJ. Alignment and integration of spatial transcriptomics data. Nat Methods. 2022;19:567–575.

[11] Traag VA, Waltman L, Van Eck NJ. From Louvain to Leiden: guaranteeing well connected communities. Sci Rep. 2019;9:5233.

[12] Maynard KR, Collado-Torres L, Weber LM, Uytingco C, Barry BK, Williams SR, et al. Transcriptome-scale spatial gene expression in the human dorsolateral prefrontal cortex. Nat Neurosci. 2021;24:425–436.

[13] Dries R, Zhu Q, Dong R, Eng CHL, Li H, Liu K, et al. Giotto: a toolbox for integrative analysis and visualization of spatial expression data. Genome Biol. 2021;22:1–31.

[14] Zhao E, Stone MR, Ren X, Guenthoer J, Smythe KS, Pulliam T, et al. Spatial transcriptomics at subspot resolution with BayesSpace. Nat Biotechnol. 2021;39:1375–1384.

[15] Pham D, Tan X, Xu J, Grice LF, Lam PY, Raghubar A, et al. stLearn: integrating spatial location, tissue morphology and gene expression to find cell types, cell-cell interactions and spatial trajectories within undissociated tissues. bioRxiv. 2020;p. 2020–05.

[16] Wolf FA, Angerer P, Theis FJ. SCANPY: large-scale single-cell gene expression data analysis. Genome Biol. 2018;19:1–5.

[17] Hu J, Li X, Coleman K, Schroeder A, Ma N, Irwin DJ, et al. SpaGCN: Integrating gene expression, spatial location and histology to identify spatial domains and spatially variable genes by graph convolutional network. Nat Methods. 2021;18:1342–1351.

[18] Xu C, Jin X, Wei S, Wang P, Luo M, Xu Z, et al. DeepST: identifying spatial domains in spatial transcriptomics by deep learning. Nucleic Acids Res. 2022;50:e131–e131.

[19] Moffitt JR, Bambah-Mukku D, Eichhorn SW, Vaughn E, Shekhar K, Perez JD, et al. Molecular,

spatial, and functional single-cell profiling of the hypothalamic preoptic region. Science. 2018;362(6416):eaau5324.

[20] Codeluppi S, Borm LE, Zeisel A, La Manno G, van Lunteren JA, Svensson CI, et al. Spatial organization of the somatosensory cortex revealed by osmFISH. Nat Methods. 2018;15:932–935.

[21] Wang X, Allen WE, Wright MA, Sylwestrak EL, Samusik N, Vesuna S, et al. Three dimensional intact-tissue sequencing of single-cell transcriptional states. Science. 2018;361:eaat5691.
