# Supplementary material for: MNMST: topology of cell networks leverages identification of spatial domains from spatial transcriptomics data
Source: Genome Biol. 2024 May 23;25:133. doi: 10.1186/s13059-024-03272-0 (PMC11112797; doi:10.1186/s13059-024-03272-0)
Supplement: Supplementary file 4 — Additional file 4: Review history. [file 13059_2024_3272_MOESM4_ESM.docx]

Review History

**First round of review**

**Reviewer 1**

**Were you able to assess all statistics in the manuscript, including the appropriateness of statistical tests used?**

No

**Were you able to directly test the methods?**

No

**Comments to author:**

The authors developed a novel method, called MNMST, for clustering spatial transcriptomics data that takes both gene expression and spatial structure into account. They benchmark their method on several data sets generated with different technologies against a number of competitor methods, and show that MNMST outperforms its competitors. However, there are some benchmarks missing, and the manucript generally suffers from lack of detail in its methods. Moreover, the implementation is not in a state that can be easily used by the community. Furthermore, the manuscript would greatly benefit from language editing. Some sections, for example lines 21-34 on page 3, are particularly difficult to follow.

major comments:
- Section 2.4: Vertical integration as described uses custom preprocessing and relies on the fact that there is a 1:1 correspondence between nodes/spots in all slices.
- The caption for Fig. S15 states that "nodes that do not exist in the main slice but exist in other slices are deleted". Does that mean that these nodes are not assigned to a spatial domain?
- In real data, 1:1 correspondence is rare, since tissue sections can become warped or torn during cutting, which is why alignment methods like PASTE/PASTE2 exist. Moreover, vertical integration as described is only applicable to spatial technologies with spots on a lattice, such as Visium, but not to FISH-based methods. However, a vertical stack of slices simply adds a 3rd dimension to the spatial coordinates, so it should be possible to run MNMST without any preprocessing using 3D spatial coordinates, which would also be applicable to FISH-based methods. I would like to see how that compares to the currently implemented vertical integration method, and if it is possible to combine it with alignment methods such as PASTE or PASTE2.

- A runtime comparison of MNMST with its competitors is missing. Many practicitioners will trade off some accuracy if it results in a much faster runtime. Therefore, the algorithms should be compared on multiple (possibly simulated) data sets with different numbers of cells/spots, ranging from ~1000 (a Visium data set) to ~1e6 (large-ish MERFISH data set)

- The implementation is a mix of Python and MATLAB code, requiring the user to switch languages during the analysis, which is not userfriendly and will severely limit adoption of the method. Requiring MATLAB, which is an expensive, proprietary software, will limit adoption further: I do not personally know anyone in the single-cell omics field who has a MATLAB license. Ideally, the implementation should not require the user to switch analysis environments, conform to the respective language's packaging guidelines, be installable as a package (for Python by running `pip install`) and play well with the rest of the community. The Python single-cell community has recently embraced scverse (https://scverse.org), which provides a Python package template following current best practices at https://github.com/scverse/cookiecutter-scverse .

minor comments:
- SCANPY is not a clustering algorithm, it is a framework for scRNAseq analysis that implements different algorithms and provides a consistent API. I'm assuming that you used Leiden clustering, but this needs to be explicit.

- Figure 2B: The caption says that SCANPY results are shown, but they are not

- Section 2.1 (overview of the model) is not really understandable without reading the methods first. It should be clearer, perhaps providing a bit more detail.

- all figures: The captions for box plots and "pirate graphs" should explain what exactly the individual components of the plot are showing (mean/median/quartiles etc). Also, the statistical tests used for calculating p-values should be included in the figure captions, and not just in the main text. Sometimes, this information is missing (e.g. line 33 on page 8).

- suppl. methods section 1.1: "the value of k is verified by Squidpy". What does that mean exactly? How was Squidpy used to determine k?

- suppl. methods section 1.1: The X matrix is not defined. I assume it's the data matrix, but it should be explicit.

- suppl. methods section 1.2: The l1 norm is convex.

- the supplementary methods are missing some references (feel free to cite additional papers that were used in the development of the method):
- Li et al. (2018) and Fang et al. (2014) should also be cited in the supplementary methods, not just in the main text
- sparse self-representation was introduced in E. Elhamifar and R. Vidal, "Sparse subspace clustering: Algorithm, theory, and applications," IEEE Trans. Pattern Anal. not Recognit. Mach. Intell., vol. 35, no. 11, pp. 2765-2781, Nov. 2013
- low rank representation was introduced in G. Liu, Z. Lin, S. Yan, J. Sun, Y. Yu and Y. Ma, "Robust Recovery of Subspace Structures by Low-Rank Representation," in IEEE Transactions on Pattern Analysis and Machine Intelligence, vol. 35, no. 1, pp. 171-184, Jan. 2013, doi: 10.1109/TPAMI.2012.88.

- Fig. S14: I assume that the x and y axes in the plots show the base-10 logarithm of the hyperparameters, but this should be explicit

- Section 2.3: The methods section should include details on how the alternative networks were constructed, e.g. which k for KNN, which kernel (e.g. Gaussian) was used for Euclidean/Cosine distance, etc

- Section 2.3: What does MNMST without joint learning look like? Was only the gene expression matrix used without spatial information?

- The description of batch effect correction in Section 2.4 is very unclear. A detailed description should be added to the methods or the supplementary methods.

**Reviewer 2**

**Were you able to assess all statistics in the manuscript, including the appropriateness of statistical tests used?**

**Yes:** I was able to do this.

**Were you able to directly test the methods?**

No

**Comments to author:**

The authors applied a multi-layer network construction coupled with joint matrix factorization to pinpoint spatial domains within spatial transcriptomics, offering a novel computational approach. Additionally, they thoroughly assessed the impact of model parameters, enhancing the model's interpretability. Despite the computational ingenuity and extensive evaluations, the study does have notable limitations and areas of concern:

1. The method's explanation within the main text and methods section is ambiguous, leading to comprehension difficulties. For instance, the importance of the high-order topological structure for the cell spatial network is critical, yet its explanation is almost absent from the manuscript, hindering the reader's grasp of the theoretical benefits of such topology. While the algorithm is elaborated in the supplementary materials, its coverage in the main text and methods section is insufficient.

2. The definition of spatial domains relies heavily on the general understanding of tissue structure, like the L1-L6 cortical layers. Despite this, there are additional variabilities within the tissue, such as cell-type differences. For example, while Scanpy seems to discern clusters of cell types ignoring spatial context, MNMST accurately determines spatial domains using both spatial and expression data. It's not certain if choosing different hyperparameters might lead the model to focus more on learning expression patterns or spatial information. Clarification is needed on how hyperparameters were managed during the benchmark tests. Additionally, have the authors noted whether spatial domains of certain tissues depend more on the expression profiles of cells, while others depend more on the spatial organization of cells?

3. Could information from images, like H&E staining or staining of cell nuclei, be helpful in identifying spatial domains? If that's the case, could this aspect be integrated into the MNMST model?

4. The sequence of discussion in the manuscript does not consistently correspond with the order of subplots in Figures 2 and 3. For example, Figure 2C is referenced before Figure 2B; Figure 3B before Figure 3A, and Figure 3D before Figure 3C. It is strongly recommended to synchronize the narrative with the figures to prevent confusion.

5. Supplementary Figures S1-S12 could be consolidated into a single comprehensive figure. Presenting similar outcomes across twelve separate figures is overly burdensome.

6. The performance of MNMST appears comparable to that of stLearn as shown in Figure S13. An explanation for this similarity would be beneficial.

7. The systematic examination of parameters presented in Figure 3 is great, but certain aspects are not clearly articulated:

7.1. The acronym 'SRL' used in Figure 3A is not defined—does it refer to a self-supervised learning strategy? It should be fully spelled out at least once.
7.2. The comparison presented in Figure 3A might be misleading, contrasting the best Pearson network results with the suboptimal SRL network results, which could confuse readers initially.
7.3. On page 8, line 28, the stated ARI values for Pearson and KNN do not seem to be supported by the corresponding dots in the ARI plot in Figure 3A.
7.4. The term 'cumulative distribution probability' in Figure 3D requires elucidation.

8. In the section "Algorithm analysis" of Supplementary Materials, it is reported that MNMST is computationally intensive, with a time complexity of 𝑂(𝑛3), and a memory requirement of 𝑂(𝑛2) this could be a limitation for large datasets with high-dimensional data.
Based on Supplementary Figures 1-12, the ARIs between DeepST and MNMST are quite similar for 10x Visium datasets (MNMST outperforms for the slice 151670). Even after calculating the Silhouette Coefficient (SC) and Davies-Bouldin (DB) scores for mouse brain posterior and coronal data, the DeepST and MNMST performances are quite similar. Based on the Supplementary Figure 1 of DeepST publication (DeepST: identifying spatial domains in spatial transcriptomics by deep learning), DeepST peformed the analysis on the same human dorsolateral prefrontal cortex slices in under 50 minutes and required less 12GB of memory. What is the computational time of MNMST on these slices? Is this comparable to DeepST? As reported in the discussion, it is possible to perform parallel computing with MNMST?
9. In the Method section "Identification and functional analysis of differentially expressed genes" it is reported that differential expression analysis of genes for each spatial domain was performed used Wilcoxon rank-sum test implemented in SCANPY. The Wilcoxon rank-sum test should not be used for differential gene expression analysis because it does not account for within-sample correlation as reported in the review by Heumos, Schaar et at. (Best practices for single-cell analysis across modalities). I would suggest to use SpatialDE as reported in the Single-cell best practices web page (https://www.sc-best-practices.org/spatial/spatially_variable_genes.html ). In addition, for the gene ontology enrichment analysis clusterProfiler was used. Was this analysis performed using the enrichGO function? If the enrichGO function was used, why the authors plotted the -log10(pval) instead of the pAdjust provided by the tool? When performing multiple tests, it is better to use the adjusted p-value because it incorporates multiple testing correction.
10. Improved documentation on the GitHub page with clear guidance on how to select optimal parameters for different spatial transcriptomics methodologies would be beneficial for users. The provided tutorial only shows the analysis for the 10x Visium dataset.


Minor point:
1. Line 2 = "many cell types (sub-populations) locating" should be changed with "many cell types (sub-populations) located";
2. Line 37 = "domains are roughly categories into two classes" should be changed with "domains are roughly categorized into two classes".

In summary, we appreciate the computational innovation of MNMST, but there are concerns regarding its description and performance. We would like to see the authors' responses to the comments above.

**Authors Response**

**Point-by-point responses to the reviewers’ comments:**

Reviewer # 1

*The authors developed a novel method, called MNMST, for clustering spatial transcriptomics data that takes both gene expression and spatial structure into account. They benchmark their method on several data sets generated with different technologies against a number of competitor methods, and show that MNMST out­performs its competitors. However, there are some benchmarks missing, and the manuscript generally suffers from lack of detail in its methods. Moreover, the implementation is not in a state that can be easily used by the community. Furthermore, the manuscript would greatly benefit from language editing. Some sections, for example lines 21-34 on page 3, are particularly difficult to follow.*

REPLY: Thanks great for your valuable comments. We address all these suggestions one by one. On the minor issues, some suggestions that are highly related are addressed together.

*S1: Section 2.4: Vertical integration as described uses custom preprocessing and relies on the fact that there is a 1:1 correspondence between nodes/spots in all slices. The caption for Fig. S15 states that “nodes that do not exist in the main slice but exist in other slices are deleted”. Does that mean that these nodes are not assigned to a spatial domain? In real data, 1:1 correspondence is rare, since tissue sections can become warped or torn during cutting, which is why alignment methods like PASTE/PASTE2 exist. Moreover, ver­tical integration as described is only applicable to spatial technologies with spots on a lattice, such as Visium, but not to FISH-based methods. However, a vertical stack of slices simply adds a 3rd dimension to the spatial coordinates, so it should be possible to run MNMST without any preprocessing using 3D spatial coordinates, which would also be applicable to FISH-based methods. I would like to see how that compares to the currently implemented vertical integration method, and if it is possible to combine it with alignment methods such as PASTE or PASTE2.*

REPLY: Thanks great for the valuable suggestion and we really appreciate it. In our original thought, we utilize one-to-one mapping as the pre-processing procedure to generate reliable data. We totally agree with the reviewer that one-to-one mapping data is limited. To address this issue, we first show MNMST also handle vertical integration of spatial transcriptomics data without one-to-one mapping. And, we then demonstrate that MNMST is a flexible network-based framework to combine other softwares, such as PASTE [1].

Vertical integration of spatial transcriptomics data without pre-processing

We hypothesize that vertically adjacent slices are very similar, and integrate them to facilitate spatial domain identification. The proposed multi-layer network model naturally provides an effective strategy for integration of multiple vertically adjacent slices. Specifically, MNMST first stacks multiple slices into one as input, and then learns the affinity graph from the stacked slice to promote identification of spatial domains. Experiments demonstrate that integrative analysis enhances performance of spatial domain identification on DLPFC data (slice 151673, 151674, 151675 and 151676) as shown in Fig. 1A. In details, ARI of spatial domains after integration is 0.570 for slice 151673, whereas it is 0.554 before integration, which proves that MNMST precisely captures complementary information from adjacent slices to promote spatial domain identification.

Then, we show MNMST effectively integrate MERFISH data, where three slices of MERFISH dataset [2] to construct 3D spatial coordinates as shown in left panel of Fig. 1B. For each slice, MNMST constructs a spatial network by concatenating three dimensional coordinates. Finally, MNMST performs spatial domain identification on the constructed multi-layer networks. Right panel of Fig. 1A visualizes spatial domains identified by DeepST [3] and MNMST on Layer 4, where these two algorithms achieve the similar perfor­mance. In details, ARI of MNMST is 0.493, whereas ARI of DeepST is 0.500. Performance of other slices is similar, which is missed to remove redundancy.

**A**	151673	151674	151675	151676

**B**	MERFISH Spatial Stacking	MNMST (ARI=0.493)	DeepST (ARI=0.500)

After integration Before integration	Ground Truth

ARI=0.554	ARI=0.610	ARI=0.631	ARI=0.588

ARI=0.570	ARI=0.651	ARI=0.631	ARI=0.582

**D**

**C**

Spatial Z

151673

151675

Slice S1	Slice S3

151676	151674

Layer 4

Layer 9

Layer 14

Pairwise Align

PASTE

Spatial 2

Center slice	MNMST

Center Slice

Integration	ARI=0.597

PASTE

Vertical Stack

Spatial 2

Spatial 2

MNMST (S1) MNMST (S3)	1

2

3

4

5

6

7

8

9

10

DeepST (S1)	DeepST (S3)	1

2

3

4

5

6

7

8

9

Spatial 2

Spatial 1

Spatial 1

DeepST ARI=0.537

1

2

3

4

5

6

7

| Spatial 1 |  | 10 |
| --- | --- | --- |

Figure 1: MNMST integrates different spatial	3transcriptomics data with various strategies: (A) Spatial

domains identified by MNMST for slices 151673, 151674, 151675, and 151676 in DLPFC data before and after integration. (B) Performance of DeepST and MNMST on the 3D coordinates of MERFISH data. (C) Performance of DeepST and MNMST on the center slice generated by PASTE [1]. (D) Performance of DeepST and MNMST on the vertical stack slice generated by PASTE.

**Vertical integration via combining MNMST and PASTE**

Furthermore, MNMST can also be flexibly integrated with current alignment algorithms for spatial transcriptomics data, such as PASTE [1], for downstream analysis. In other words, PASTE generates integrated data, and MNMST is directly applied to the generated data to identify spatial domains. PASTE [1] provides two ways to integrate spatial transcriptomics data, i.e., central slice as well as vertical stacking, as shown in Fig. 1C and D.

Performance of DeepST and MNMST on the central slice for DLPFC 151673-151676 is shown in Fig. 1C, where ARI of MNMST and DeepST is 0.597, and 0.537, respectively. It demonstrates that MNMST is more precise to capture structure of spatial domains in the integrated central slide. Finally, we also verify the performance of DeepST and MNMST on the vertical stacking for two slices from the same mouse with breast cancer [4] is shown in Fig. 1D, where MNMST significantly outperforms DeepST because the spatial domains identified by MNMST for these two slices are highly consistent, whereas these obtained by DeepST differ greatly. There results demonstrate that MNMST is also more precise to analyze data generated by other softwares, indicating that MNMST can be combined with other tools to execute complicated tasks.

**Conclusion: MNMST is a flexible and efficient multi-layer network-based algorithm for the vertical integration of spatial transcriptomics data. Furthermore, it can directly be combined with other softwares, such as PASTE [1], to perform spatial domain identification, bringing in convenience for users.**

**Modification:**

We made the following modification:

- We re-organize section 2.4 by modifying the experiments (Page: 12-13, Section: 2.4, Color: red); - Experimental results in Fig. 1 are added (Supplmentary Figure: 7).

***S2****: A runtime comparison of MNMST with its competitors is missing. Many practicitioners will trade off some accuracy if it results in a much faster runtime. Therefore, the algorithms should be compared on mul­tiple (possibly simulated) data sets with different numbers of cells/spots, ranging from 1000 (a Visium data set) to 1e6 (large-ish MERFISH data set)*

**REPLY:** Thanks great for the valuable suggestion and we really appreciate it. To address this issue, we first make a comparative comparison among various algorithms on the running time with different spatial tran-scriptomics datasets, including 10 × Genomics and MERFISH data. And, we then accelerate the proposed algorithm with GPU.

**Space and time of various algorithms**

**Platform:** For a fair comparison, all algorithms are executed on the same platform with Intel i9 CPU, 64GB RAM, and an NVIDIA RTX4090.

On the 10 × Genomics data, we independently execute each algorithm for each slice of DLPFC data [5], where the distributions of running time and space of algorithms are summarized in Fig. 2 A1 and A2, respectively. From Fig. 2 A1, it is easy to conclude that Giotto and BayesSpace are slower than stLearn and MNMST, and SCANPY, SpaGCN and DeepST are faster than others. Specifically, running time of MNMST is 4.3 ± 2.5 minutes, while that is 10.6 ± 2.9 (Giotto), 6.3 ± 2.2 (BayesSpace), 3.6 ± 0.4 (stLearn), 2.2 ± 0.4 (DeepST), 1.4 ± 0.3 (SpaGCN), 0.6 ± 0.1 (SEDR) and 0.1 ± 0.0 (SCANPY), respectively. Fig. 2 A2 illustrates distributions of spaces for various algorithms for DLPFC data, where space complexity of Gitto, stLearn and DeepST much higher than others. In details, space of MNMST is 3.6 ± 0.1 Gigabytes, while that is 5.5 ± 0.4 (Giotto), 1.2 ± 0.1 (BayesSpace), 8.5 ± 1.5 (stLearn), 9.3 ± 0.7 (DeepST), 1.7 ± 0.1 (SpaGCN), 1.9 ± 0.1 (SEDR) and 1.5 ± 0.2 (SCANPY), respectively.

From Fig. 2 A1 and A2, it is easy to assert that

- Gitto and stLearn are time- and space-consuming, hampering the applications of identification of spatial domains in ST data. And, DeepST reduces running time by sacrificing space, whereas BayesSpace reduces space complexity by sacrificing running time.

12.5

(A2)

15

(A1

10.0

10

Memory (G)

Time (min)

7.5

5.0

5

2.5

0

1000

(B1)

45

979.43

(B2)

42.45

40

800

SCANPY
DeepST
MNMST

SCANPY
DeepST
MNMST

35

600

Memory (G)	Memory (G)	Memory (G)

Time (min)	Time (min)	Time (min)

533.16

30

400

25

232.25

20

200

19.

.81

10.44

**\\**

15

13.44

4

3.84

10

8.35

2.36

8.04

2

5.60

4.15

5

1.2

4.01

3.41

0.29	0.49	0.19

0.07 ^0.22^ 0.08

1.95

1.46

1.47

1.50

1.51

1.51

0.25

0.97 1.32

1.21

0.22

0.20

0.21

0

0

1k 5k 10k 15k 20k 25k 30k

1k 5k 10k 15k 20k 25k 30k

5

MNMST (CPU) MNMST (GPU)

MNMST (CPU) MNMST (GPU)

9.82	9.90

(C1)

(C2)

4.8

9

4.71	4.69

4.6

8

4.56

4.55

4.52

4.48

44

.37

4.4

7

4.37

6.62

4.33

.31	4.27

4.25

4.2

6

5.29

5

4

4.

4

3.79 3.78

3.90	3.91

3.8

3.67 3.65

3.66

3.61

3.60	3.6

3.1

3.61

3

.

3.6

2.95

3.54	3.55

3.50

2

3.4

1

3.2

0.10

0.10	0.12

0.11	0.08

0.08	0.10

.09 0.08

.08	0.08

.08

0

3

45

979.43

1000

(D1)

(D2)

42.45

800

40

MNMST (CPU) MNMST (GPU)

MNMST (CPU) MNMST (GPU)

600

35

533.16

400

30

29.45

232.25

200

25

**\\**

72.81

10.44

10

9.55

20

8

15

6.3

6

12.24

10

4

8.35

8.27

3.42

5.44

5

2

4.15

3.12	3.71

1.35

1.95

0.97

0.51

0.29 0.03

0.14

0

0

1k 5k 10k 15k 20k 25k 30k

1k 5k 10k 15k 20k 25k 30k

Figure 2: Running time and space of algorithms for different spatial transcriptomics data: (A1) Distributions of running time of algorithms on the DLPFC dataset (minutes), (A2) Distributions of space of algorithms on the DLPFC dataset (Gigabyte), (B1) Running time of algorithms on MERFISH data with various sizes, (B2) Running time of algorithms on MERFISH data with various sizes, (C1) Running time of MNMST with and without acceleration on the DLPFC dataset, (C2) Space of MNMST with and without acceleration on the DLPFC dataset, (D1) Running time of MNMST with and without acceleration on the DLPFC dataset, and (D2) Space of MNMST with and without acceleration on the MERFISH dataset.

- SCANPY, SEDR and SpaGCN are efficient in terms of running time and space, whereas performance of these is undesirable. In other words, these algorithms improve efficiency by sacrificing accuracy of algorithms.

- MNMST reaches a good tradeoff between space and running time. Furthermore, it achieves the best performance on the identification of spatial domains, demonstrating that MNMST provides an excellent alternative for current algorithms.

On the MERFISH data [2], we validate efficiency of algorithms by increasing the number of spots/cells from 1,000 to 30,000. Since SEDR , SpaGCN, and DeepST employ graph convolutional network (GCN), and DeepST is superior to them in terms of accuracy. Therefore, only SCANPY, DeepST and MNMST are selected for a comparison. Fig. 2 B1 and B2 describe the running time and space of algorithms with various sizes of data respectively, where missing bar represents failure of algorithms to address the corresponding data. When the number of spots is less than or equal to 20,000, MNMST is worse than DeepST on both space and time. However, MNMST addresses ST data with more than 20,000 spots that DeepST fails to handle. The reason why DeepST fails to address large-scale ST data is that it employs deep learning to obtain features of spots/cells, where the number of parameters for learning exponentially increases, resulting in the expensive time complexity.

**Accelerating MNMST with GPU**

Even though we demonstrate that MNMST reaches a good balance between time and space with the best performance of identifying spatial domains, its efficiency is still undesirable since it fails to address large-scale ST data. To solve this problem, we accelerate MNMST with graphic processing unit (GPU), i.e., accelerating MNMST with hardware. The running time and space of MNMST with CPU and GPU for DPLDC are summarized in Fig. 2 C1 and C2 respectively, where MNMST(GPU) accelerates 100 times by sacrificing extra 20% space, demonstrating that GPU is promising for improving applicability of MNMST. The acceleration strategy is also efficient for MERFISH, where the improvement of time and space is shown in Fig. 2 D1 and D2, respectively.

**Conclusion: MNMST reaches a good balance between time and space, and it achieves the best perfor­mance with the acceptable running time. Furthermore, GPU dramatically accelerates speed of MNMST, which is applicable for large-scale ST data.**

**Modification:**

We made the following modification:

- A comparative comparison among typical algorithms for efficiency on various spatial transcriptomics data is added (Section: 2.3, Page: 9, Color: Red, Supplementary Figure: S4);

- The GPU version of MNMST software is also provided (website: <https://github.com/xkmaxidian/> MNMST/blob/main/MNMST_gpu.py).

*S3: The implementation is a mix of Python and MATLAB code, requiring the user to switch languages during the analysis, which is not userfriendly and will severely limit adoption of the method. Requiring MATLAB, which is an expensive, proprietary software, will limit adoption further: I do not personally know anyone in the single-cell omics field who has a MATLAB license. Ideally, the implementation should not require the user to switch analysis environments, conform to the respective language’s packaging guidelines, be installable as a package (for Python by running ‘pip install‘) and play well with the rest of the community. The Python single-cell community has recently embraced scverse (*[*https://scverse.org*](https://scverse.org)*), which provides a Python package template following current best practices at* [*https://github.com/scverse/cookiecutter-scverse.*](https://github.com/scverse/cookiecutter-scverse.)

REPLY: Thanks great for the suggestion. To address this issue, we first re-write the code of MNMST with python, and now the users do not need to switch environments, and then we extend MNMST with GPU. Now, we provide two versions of MNMST, CPU- and GPU-version for users to select the better one. We already submit MNMST to python community, and it is now under processing (<https://pypi.org/project/mnmstpy/).>

Modification:

We made the following modification:

- The GPU and CPU version of MNMST software are provided (website: <https://github.com/xkmaxidian/> MNMST).

Minor Problems

S4: *SCANPY is not a clustering algorithm, it is a framework for scRNAseq analysis that implements differ­ent algorithms and provides a consistent API. I’m assuming that you used Leiden clustering, but this needs to be explicit. Figure 2B: The caption says that SCANPY results are shown, but they are not.*

REPLY: Thanks great for the suggestion. Yes, we utilize Leiden clustering for SCANPY, and now we clarify it to avoid confusion. And, we proofreading the whole manuscript, and now we remove all these typos. Thanks for the considerable suggestion.

S5: *Section 2.1 (overview of the model) is not really understandable without reading the methods first. It should be clearer, perhaps providing a bit more detail.*

REPLY: Thanks great for the suggestion. To improve the reading and understanding of MNMST, we re­organize the section of overview of the multi-layer network model for spatial domain identification, where the rationale and components of the proposed algorithm are described.

Overview of the multi-layer network model for spatial domain identification

To smooth the reading and understanding of this study, we first describe the rationale of MNMST in this section (more technical description can be referred in section of Methods). For the sake of convenience, we use cells to represent measurement units in spatial transcriptomics, which can be freely replaced to spots in various platforms, such as 10× Visium.

For any spatial transcriptomics data, the traditional clustering approaches [6] first perform feature learn­ing by preserving spatial and expression information of cells, and then identify spatial domains by clustering the low-dimensional features of cells. However, these algorithms are criticized for the undesirable performance because the learned features fail to model and characterize the intrinsic and indirected relations among cells. To overcome this limitation, network-based algorithms, such as DeepST [3], SpaGCN [7] and STAGATE [8], first construct a graph for cells by integrating spatial, expression information, etc. And, these algorithms then learn features by exploiting topological structure of the constructed cell network, where the indirected relations among cells are exploited and preserved in features of cells, thereby dramatically enhancing perfor­mance of spatial domain identification.

Even though network-based methods significantly outperform the traditional approaches by promoting quality of features of cells, they still have several typical limitations. First of all, current algorithms construct cell network by directly concatenating spatial, expression and etc., to calculate distances of cell pairs, which are criticized for ignoring heterogeneity of spatial transcriptomics data, reducing quality and reliability of cell networks. Although there are many alternatives for measuring distances among cells, it is difficult to select an appropriate manner to precisely characterize distances of cells (how measurement of distance effects performance of algorithms is studied in section 2.3). Finally, the learned low-dimensional features of cells

**A**

Spots

*y*

0

1

1

0

1

Spots

0 _0_1

...Spots

1

1 1

1

*x*

0

Spatial Location	Distance Matrix	Indirected relations	Adjacency Matrix

Cell Spatial Network

Genes

Genes

Data preprocessing

Data augmentation

Spots

Scale PCA

Adjacency matrix Gene expression

Spots

Gene Expression

Enhanced Gene Expression

Self Representation

Cell Expression Network

**B**

Cell expression network

Joint Matrix Factorization

Affinity Graph

≈

*_F_*[*e*]

of Cells

*_G_*[*e*]

*_M_*[*s*]

[*e*]

*G*

Joint Learing

*B*

*_M_*[*s*]

Common Subspace

≈

*F* ^[^*^s^*^]^

Cell spatial network

Multi-Layer Networks

**C**

Tumor

Left Slice	Right Slice

Healthy

Spatial domain

DEGs detection Data integration

...

Tumor edge

Spatial domain identification	Topology analysis	Integrative analysis of data	Other functions

Figure 3: Overview of multi-layer network model (MNMST) for spatial domain identification. (A) Cell multi-layer network construction. MNMST constructs cell spatial network of cells by exploiting indirected relations among cells, and it learns cell expression network of cells with self-representation and local preservation techniques in machine learning. (B) Spatial and expression feature learning. MNMST jointly factorizes cell multi-layer networks with non-negative matrix factorization by projecting cells into a common subspace to learn compatible features of cells. And, it simultaneously learns an affinity graph of cells, which reflects structure of the constructed multi-layer networks. (C) Down-stream analysis of affinity graph of cells, including spatial domain identification, topology analysis, integrative analysis of spatial transcriptomics data.

only preserved preserve topological structure of cell networks, which neglect relations among spatial and expression information, failing to fully characterize structure of spatial domains.

Here, we address these issues by proposing the multi-layer network model (MNMST) to characterize and identify spatial domains in spatial transcriptomics data by integrating gene expression and spatial information of cells (Fig. 3). To remove heterogeneity of spatial transcriptomics data, MNMST constructs two cell networks for spatial and expression data, converting heterogeneous data into homogeneous graphs. Furthermore, MNMST learns features of cells by jointly exploiting topological structure of cell spatial and expression network, where spatial and expression information are simultaneously learned, thereby improving quality of features of cells. Specifically, as shown in Fig. 3, MNMST consists of three major components, i.e., cell multi-layer network construction, cell feature learning, and spatial domain identification. On multi-layer network construction issue, MNMST first employs Euclidean distance to construct cell spatial network by exploiting indirected topological structure. Then, it automatically learns cell expression networks by utilizing self-representation learning (SRL) with local preservation constraint, where cell spatial data are augmented to enhance quality of expression profiles of cells (Fig. 3A, Methods). In this case, MNMST not only avoids selecting measurements of distances for cells, but also enhances quality of constructed graph.

On cell feature learning issue, current algorithms independently learn spatial and expression features of cells, ignoring the latent relation among them. To overcome this limitation, MNMST simultaneously learns spatial and expression features of cells by jointly decomposing cell multi-layer networks with nonnegative matrix factorization, ensuring the compatibility and quality of features (Fig. 3B). To perform spatial domain identification, MNMST automatically learns an affinity graph for cells with low-rank self-representation technique, which improves interpretability of spatial domains (Fig. 3B). On the basis of affinity graph, MNMST performs graph clustering by exploiting topological structure of graph to identify spatial domains (Fig. 3C).

Notice that, as far as we know, MNMST is the first multi-layer network model for spatial domain iden­tification, which provides an alternative for modeling and analyzing spatial transcriptomics data.

Modification:

We made the following modification:

- The overview of MNMST is re-organized (Page: 4-6, Section: 2.1, Color: red).

S6: *all figures: The captions for boxplots and ”pirate graphs” should explain what exactly the individual com­ponents of the plot are showing (mean/median/quartiles etc). Also, the statistical tests used for calculating p-values should be included in the figure captions, and not just in the main text. Sometimes, this information is missing (e.g. line 33 on page 8).*

REPLY: Thanks great for the suggestion, and we correct all these issues in Figures.

S7: *(1). suppl. methods section 1.1: ”the value of k is varies by Squidpy”. What does that mean exactly? How was Squidpy used to determine k? (2). suppl. methods section 1.1: The X matrix is not defined. I assume it’s the data matrix, but it should be explicit. (3). suppl. methods section 1.2: The l1 norm is convex. (4). I assume that the x and y axes in the plots show the base-10 logarithm of the hyperparameters, but this should be explicit.*

REPLY: Thank you for the suggestion.

- MNMST first constructs a initial cell spatial network with using *K*-nearest neighbor algorithm, where the *k* nearest neighbors are selected for each cell. By following Squidpy [9], the number of neighbors *k* is 6 for 10 × Genomics data, 8 for Slide-seq and Stereo-seq data, and 15 for imaging-based platforms.

- *X* is the low-dimensional cell expression feature matrix, which is obtained with PCA on the cell expression profiles.

- We already correct this issue.

- We already modify hyperparameter analysis.

Modification:

We made the following modification:

- The explanation for parameter *k* is added (Section: 5.2, Page: 19, Color: red); - The explanation for matrix *X* is added (Section: 5.1, Page: 19, Color: red);

- The description and plot of hyperparameters are corrected (Additional file1: Section 1, Supplementary Figure: S4).

*S8: - the supplementary methods are missing some references (feel free to cite additional papers that were used in the development of the method):*

*- Li et al. (2018) and Fang et al. (2014) should also be cited in the supplementary methods, not just in the main text*

*- sparse self-representation was introduced in E. Elhamifar and R. Vidal, “Sparse subspace clustering: Algorithm, theory, and applications,” IEEE Trans. Pattern Anal. not Recognit. Mach. Intell., vol. 35, no. 11, pp. 2765-2781, Nov. 2013*

*- low rank representation was introduced in G. Liu, Z. Lin, S. Yan, J. Sun, Y. Yu and Y. Ma, “Robust Recovery of Subspace Structures by Low-Rank Representation,” in IEEE Transactions on Pattern Analysis and Machine Intelligence, vol. 35, no. 1, pp. 171-184, Jan. 2013, doi: 10.1109/TPAMI.2012.88.*

REPLY: Thanks great the suggestion, and references are added in supplementary materials.

Figure 4: Performance of MNMST with and without joint learning for DLPFC data in terms of ARI.

*S9: 1. Section 2.3: The methods section should include details on how the alternative networks were constructed,*

*e.g. which k for KNN, which kernel (e.g. Gaussian) was used for Euclidean/Cosine distance, etc.*

*2. Section 2.3: What does MNMST without joint learning look like? Was only the gene expression matrix*

*used without spatial information?*

**REPLY**: Thank you for the suggestion, and we address these issues one by one.

On the KNN issue, MNMST first constructs a initial cell spatial network W[s] with using K-nearest neighbor algorithm (by following Squidpy [9], the number of neighbors k is 6 for 10 × Genomics data, 8 for

Figure 5: Performance of MNMST on removing batch effects of spatial transcriptomics data. (A) UMAP plots of spatial integrated algorithms, including MNMST, DeepST, stLearn and SEDR, for slices 151673, 151674, 151675, and 151676 in DLPFC data. They represent batches, recognition spatial domains, and ground truth labels, respectively. (B) UMAP plots of the S1, S3 and stacked slice before integration. UMAP plots of spatial domains identified by MNMST for slice S1 and S3 in mouse breast cancer data. (C) UMAP plots of the S1, S3 and stacked slice after integration. UMAP plots of spatial domains identified by MNMST for slice S1 and S3 in mouse breast cancer data.

Modification:

We made the following modification:

- The detailed description of KNN is added (Section: 5.2, Page: 19, Color: red);

- The comparison between joint learning and non-joint learning is added (Section: 2.2, Figure: 3C).

*S10: The description of batch effect correction in Section 2.4 is very unclear. A detailed description should be added to the methods or the supplementary methods.*

REPLY: Thank you for the suggestion. We address this issue by investigate the capability of MNMST to remove batch effect from spatial transcriptomics data. Up-to-date methods (DeepST, stLearn, and SEDR) address these issues with additional computational modules, while MNMST can be directly applied without modifying algorithms. Specifically, it constructs cell networks from multiple batches by concatenating these in each batch, and then executes MNMST on the constructed graphs. With four DLPFC slices (1516173­151676), MNMST achieves the best performance as shown in Fig. 5A, where the ARI of MNMST is 0.609 (ARI of DeepST: 0.448, ARI of stLearn: 0.248, and ARI of SEDR: 0.421). Interestingly, integration of multiple slices enhances the performance of spatial domain identification, which shows that MNMST can effectively remove batch effect by retaining biological content.

Except for DLPFC data, mouse slices with breast cancer are aligned vertically using PASTE, where there is obvious batch effect as shown in Fig. 5B. It is observed that spatial domains in vertically adjacent slices are highly consistent after integration Fig. 5C, whereas these domains are mixed without integration (Fig. 5B). These results demonstrates that MNMST effectively leverages spatial information to correct batch effects in spatial transcriptomics data.

Modification:

We made the following modification:

- The experiments for removing batch effect are updated (Page: 12-13, Section: 2.4, Color: red, Supple­mentary Figure: 8).

**Reviewer # 2**

*The authors applied a multi-layer network construction coupled with joint matrix factorization to pinpoint spatial domains within spatial transcriptomics, offering a novel computational approach. Additionally, they thoroughly assessed the impact of model parameters, enhancing the model’s interpretability. Despite the com­putational ingenuity and extensive evaluations, the study does have notable limitations and areas of concern.*

**REPLY:** Thank you for your valuable comments, and we address all these suggestions one by one.

*S1: The method’s explanation within the main text and methods section is ambiguous, leading to compre­hension difficulties. For instance, the importance of the high-order topological structure for the cell spatial network is critical, yet its explanation is almost absent from the manuscript, hindering the reader’s grasp of the theoretical benefits of such topology. While the algorithm is elaborated in the supplementary materials, its coverage in the main text and methods section is insufficient.*

**REPLY:** Thanks great for the suggestion. To improve the reading and understanding of MNMST, we re­organize the section of overview of the multi-layer network model for spatial domain identification, where the rationale and components of the proposed algorithm are described.

**Overview of the multi-layer network model for spatial domain identification**

To smooth the reading and understanding of this study, we first describe the rationale of MNMST in this section (more technical description can be referred in section of Methods). For the sake of convenience, we use cells to represent measurement units in spatial transcriptomics, which can be freely replaced to spots in various platforms, such as 10× Visium.

For any spatial transcriptomics data, the traditional clustering approaches [6] first perform feature learn­ing by preserving spatial and expression information of cells, and then identify spatial domains by clustering the low-dimensional features of cells. However, these algorithms are criticized for the undesirable performance because the learned features fail to model and characterize the intrinsic and indirected relations among cells. To overcome this limitation, network-based algorithms, such as DeepST [3], SpaGCN [7] and STAGATE [8], first construct a graph for cells by integrating spatial, expression information, etc. And, these algorithms then learn features by exploiting topological structure of the constructed cell network, where the indirected relations among cells are exploited and preserved in features of cells, thereby dramatically enhancing perfor­mance of spatial domain identification.

Even though network-based methods significantly outperform the traditional approaches by promoting quality of features of cells, they still have several typical limitations. First of all, current algorithms construct cell network by directly concatenating spatial, expression and etc., to calculate distances of cell pairs, which are criticized for ignoring heterogeneity of spatial transcriptomics data, reducing quality and reliability of cell networks. Although there are many alternatives for measuring distances among cells, it is difficult to select an appropriate manner to precisely characterize distances of cells (how measurement of distance effects performance of algorithms is studied in section 2.3). Finally, the learned low-dimensional features of cells only preserved preserve topological structure of cell networks, which neglect relations among spatial and expression information, failing to fully characterize structure of spatial domains.

Here, we address these issues by proposing the multi-layer network model (MNMST) to characterize and identify spatial domains in spatial transcriptomics data by integrating gene expression and spatial information of cells (Fig. 6). To remove heterogeneity of spatial transcriptomics data, MNMST constructs two cell networks for spatial and expression data, converting heterogeneous data into homogeneous graphs. Furthermore, MNMST learns features of cells by jointly exploiting topological structure of cell spatial and expression network, where spatial and expression information are simultaneously learned, thereby improving quality of features of cells. Specifically, as shown in Fig. 6, MNMST consists of three major components, i.e., cell multi-layer network construction, cell feature learning, and spatial domain identification. On multi-layer

**A**

Spots

*y*

0

1

1

0

1

Spots

0 _0_1

...Spots

1

1 1

1

*x*

0

Spatial Location	Distance Matrix	Indirected relations	Adjacency Matrix

Cell Spatial Network

Genes

Genes

Data preprocessing

Data augmentation

Spots

Scale PCA

Adjacency matrix Gene expression

Spots

Gene Expression

Enhanced Gene Expression

Self Representation

Cell Expression Network

**B**

Cell expression network

Joint Matrix Factorization

Affinity Graph

≈

*_F_*[*e*]

of Cells

*_G_*[*e*]

*_M_*[*s*]

[*e*]

*G*

Joint Learing

*B*

*_M_*[*s*]

Common Subspace

≈

*F* ^[^*^s^*^]^

Cell spatial network

Multi-Layer Networks

**C**

Tumor

Left Slice	Right Slice

Healthy

Spatial domain

DEGs detection Data integration

...

Tumor edge

Spatial domain identification	Topology analysis	Integrative analysis of data	Other functions

Figure 6: Overview of multi-layer network model (MNMST) for spatial domain identification. (A) Cell multi-layer network construction. MNMST constructs cell spatial network of cells by exploiting indirected relations among cells, and it learns cell expression network of cells with self-representation and local preservation techniques in machine learning. (B) Spatial and expression feature learning. MNMST jointly factorizes cell multi-layer networks with non-negative matrix factorization by projecting cells into a common subspace to learn compatible features of cells. And, it simultaneously learns an affinity graph of cells, which reflects structure of the constructed multi-layer networks. (C) Down-stream analysis of affinity graph of cells, including spatial domain identification, topology analysis, integrative analysis of spatial transcriptomics data.

14

network construction issue, MNMST first employs Euclidean distance to construct cell spatial network by exploiting indirected topological structure. Then, it automatically learns cell expression networks by utilizing self-representation learning (SRL) with local preservation constraint, where cell spatial data are augmented to enhance quality of expression profiles of cells (Fig. 6A, Methods). In this case, MNMST not only avoids selecting measurements of distances for cells, but also enhances quality of constructed graph.

On cell feature learning issue, current algorithms independently learn spatial and expression features of cells, ignoring the latent relation among them. To overcome this limitation, MNMST simultaneously learns spatial and expression features of cells by jointly decomposing cell multi-layer networks with nonnegative matrix factorization, ensuring the compatibility and quality of features (Fig. 6B). To perform spatial domain identification, MNMST automatically learns an affinity graph for cells with low-rank self-representation technique, which improves interpretability of spatial domains (Fig. 6B). On the basis of affinity graph, MNMST performs graph clustering by exploiting topological structure of graph to identify spatial domains (Fig. 6C).

Notice that, as far as we know, MNMST is the first multi-layer network model for spatial domain iden­tification, which provides an alternative for modeling and analyzing spatial transcriptomics data.

Modification:

We made the following modification:

- The overview of MNMST is re-organized (Page: 4-6, Section: 2.1, Color: red).

*S2: The definition of spatial domains relies heavily on the general understanding of tissue structure, like the L1-L6 cortical layers. Despite this, there are additional variabilities within the tissue, such as cell-type differences. For example, while Scanpy seems to discern clusters of cell types ignoring spatial context, MNMST accurately determines spatial domains using both spatial and expression data. It’s not certain if choosing different hyperparameters might lead the model to focus more on learning expression patterns or spatial information. Clarification is needed on how hyperparameters were managed during the benchmark tests. Additionally, have the authors noted whether spatial domains of certain tissues depend more on the expression profiles of cells, while others depend more on the spatial organization of cells?*

REPLY: Thanks great for the valuable suggestion. To address this issue, we investigate how parameters effect performance of MNMST with data generated by various platforms, and find that these parameters differ for different data.

By applying MNMST to spatial transcriptomics data generated by different platform, we investigate how ARI of MNMST changes as values of parameters vary as shown in Fig. 7, where panel A is for 10x Genomics, and B for MERFISH, and C for STARmap data, respectively. Fig. 7 A depicts how ARI of MNMST changes as parameters ('y, A) increase from 0.1 to 80 for DLPFC data, where MNMST is quit stable. In details, as parameter 'y increases from 0.1 to 1, ARI of MNMST dramatically improves. And, MNMST is quit stable when 'y E [1, 80]. Moreover, as parameter A E [0.1, 20], MNMST achieves the best performance. However, its performance dramatically decreases when A > 20.

There are several possible reasons to explain this tendency. When 'y is too small, the contribution of structure constraint for cell affinity graph is limited, thereby reducing performance of MNMST. Increasing value of parameter 'y ensures structure of spatial domains in affinity graph, enhancing performance of MN-MST. Furthermore, when parameter A is too large, MNMST over-emphasizes sparsity of cell affinity graph, resulting in unbalance between structure of cell affinity network and spatial domains.

Fig. 7 B depicts how ARI of MNMST changes as parameters ('y, A) increase from 0.1 to 80 for MERFISH data [2], where MNMST achieves the best performance if A E [10,30], and 'y E [20, 80]. Furthermore, Fig. 7 C depicts how ARI of MNMST changes as parameters ('y, A) increase from 0.1 to 80 for STARmap data [13], where MNMST obtains the optimal performance as A E [40,80], and 'y E [1, 40]. Careful comparison among panels of Fig. 7 demonstrates that parameters of MNMST are highly associated with spatial transcriptomics data because different platforms generate data with unique intrinsic structures.

Figure 7: Parameter effects of MNMST for various data: (A) ARI vs parameter (γ, λ) for 10x Genomics data, (B) ARI vs parameter (γ, λ) for MERFISH data, and (C) ARI vs parameter (γ, λ) for STARmap data.

**Conclusion**: Parameters of MNMST differ for various types of spatial transcriptomics data largely due to the intrinsic structure of data, and we suggest λ E [1,80], and γ E [1, 20] for 10 x Genomics data, λ E [10,30], and γ E [20, 80] for MERFISH data, and λ E [40,80], and γ E [1, 40], respectively.

**Modification:**

We made the following modification:

- The parameter analysis is updated (Page: 9, Section: 2.3, Color: red, Supplementary Figure: S5, Supplementary Materials: Section 1.7).

*S3: Could information from images, like H*&*E staining or staining of cell nuclei, be helpful in identifying spatial domains? If that’s the case, could this aspect be integrated into the MNMST model?*

**REPLY:** Thanks great for the excellent suggestion, and we really appreciate it. To be honest, it is one of the critical problems in my lab now. Actually, MNMST is a flexible multi-layer network framework for ST data, which can be easily extended to incorporate morphological information of images. To address this issue, we first provided two possible extension of MNMST, and then explain why morphological information is absent.

**Extension of MNMST**

There are two possible ways to extend MNMST for morphological information of ST data by either manipulating input data (MNMST-DI) or modifying structure of MNMST (MNMST-MS) as shown in Fig. 2, which are summarized as

- MNMST-DI first integrates spatial, expression and image information into a cell similarity network and then input the constructed network into MNMST. In details, we take image processing to obtain morphological features of spots, and then concatenate morphological features with expression feature, and spatial features for each spots. Notice that there are many sophisticated alternatives can be selected, and we aim to show the flexibility of MNMST, rather than to choose the best components. The advantage of MNMST-DI is that it only needs pre-processing of ST data without changing structure of MNMST.

- MNMST-MS first learns features of spots from images, and then construct morphological similarity network for spots. Then MNMST-MS updates update rules for three-layer networks such that the cell spatial network, cell expression network, and morphological similarity network are jointly learned. In this case, the objective function of MNMST-MS is formulated as

**(A)**

**(B)	(C)**

0.7

0.6

0.6

0.6

0.5

0.4

0.4

ARI

ARI

ARI

0.4

0.2

0.2

0.3

0

0

0.2

0.1

**A**

*y*

0

1

1

0

1

0 _0_1

Spots

...

1

1 1

(*x, y, z*)

**+	=**

1

*x*

0

Spatial proximity

&

morphological similarity

Cell Spatial Network

Spatial Location (*x, y*)

H&E staining image

Input

Cell expression network

Joint Matrix Factorization

Affinity Graph

*_G_*[*e*]

*_M_*[*s*]

*_F_*[*e*]

of Cells

[*e*]

*G*

Joint Learing

*B*

*_M_*[*s*]

Common Subspace

*F* ^[^*^s^*^]^

Multi-Layer Networks

Cell spatial network

**B**

...

Image Processing

Segment

...

Morphological Similarity

Morphological feature	Matrix

H&E staining image

Image tiles	Feature learning algorithm

Input

Cell expression network

*_G_*[*e*]

*_G_*[*e*]

*_M_*[*s*]

Joint Matrix Factorization

*_F_*[ *e*]

Affinity Graph

Cell spatial network

*_W_*[ *i*]

Joint

*_M_*[*s*]

*_F_*[*s*]

*B*

Common Subspace

Learning

*W* ^[^*^i^*^]^

Input

*_F_*[ *i*]

Expression Spatial

Morphological Similarity Network

Figure 8: Extension of MNMST: (A) MNMST-DI incorporates images of spots/cells by data manipulation with changing structure of algorithms, and (B) MNMST-MS modifies structure of MNMST for three-layer networks by constructing morphological similarity network.

17

| **A**  Histological image | Grount Truth/ID:151675 | MNMST-DI  ARI=0.508 | MNMST-MS  ARI=0.373 | MNMST  ARI=0.631 |
| --- | --- | --- | --- | --- |

Layer 1 Layer 2 Layer 3 Layer 4 Layer 5 Layer 6 WM

Histological image	Grount Truth/ID:151507	ARI=0.426	ARI=0.155	ARI=0.523

Layer 1 Layer 2 Layer 3 Layer 4 Layer 5 Layer 6 WM

Histological image

Grount Truth/ID: 151671

ARI=0.467	ARI=0.373	ARI=0.561

Layer 3 Layer 4 Layer 5 Layer 6 WM

1

2

3

4

5

6

7

1

2

3

4

5

6

7

1

2

3

4

5

11

12

13

14

15

16

17

18

19

20

**B**	Grount Truth/ID: BRCA

Figure 9: Performance of variants of MNMST for various ST data: (A) DLPFC, and (B) Breast cancer data.

1

2

3

4

5

6

7

8

9

10

Healthy Healthy Tumor_edge Tumor_edge Tumor_edge Tumor_edge Tumor_edge Tumor_edge DCIS/LCIS DCIS/LCIS DCIS/LCIS DCIS/LCIS DCIS/LCIS IDC IDC IDC IDC IDC IDC IDC

ARI=0.537

ARI=0.513	ARI=0.598

18

**Performance of MNMST-DI and MNMST-MS**

Performance of MNMST, MNMST-DI and MNMST-MS on different data is summarized in Fig. 9, where panel A is for DLPFC, B for Breast cancer data, respectively. To our surprise, integrating morphological information reduces performance of MNMST. In details, ARI of MNMST is 0.631 for slide 151675 in DLPFC, whereas that is 0.508 for MNMST-DI, and 0.373 for MNMST-MS, respectively. Furthermore, ARI of MNMST is 0.598 for breast cancer data, whereas that is 0.537 for MNMST-DI, and 0.513 for MNMST-MS, respectively.

Interestingly, we also observed that when morphological information is incorporated as one layer in the multi-layer network model, MNMST-MS can identify domains with significant morphological changes. For example, in Fig. 9A, MNMST-MS effectively identifies disrupted regions in slice 151507 and the gray-white area in slice 151675. In the BRCA region, both MNMST variants identify more accurate tumor boundaries (black dashed lines in Fig. 9B) compared to the standard MNMST. Additionally, MNMST-DI effectively separates healthy regions from the whole slice. One possible reason is that morphological information only provides partial information for spatial domains, posing a great challenge on design algorithms.

Actually, integrating of morphological information reduces performance of spatial domain identification is also reported in Refs. [18] via <https://github.com/AltschulerWu-Lab/MUSE/issues/3>. There are sev­eral possible reasons to explain why integration of images of ST data results in reduction of performance. First, features of images and expression are heterogeneous, bringing in incompatibility of features. Second, complexity of tissues is also one of the key factors. For example, expression and spatial information are suffi­cient to comprehensively characterize tissues whose structure is clear, where image information is redundant. However, the complicated tissues requires image features to further reveal the intrinsic structure.

**Why image is missing**: To be honest, we have progression on this issue such as by setting a parameter to control the relative importance of image features, which enhances performance of identifying spatial domains. But, we currently still cannot figure out the following problems:

- Under what circumstance image information is needed for spatial domain identification? What types of ST data is suitable for the integration of image information?

- How to remove heterogeneity of image and expression features of spots/cells to improve performance of MNMST.

In order to provide rigorous assertion and reliable tools, we do not extend MNMST to images. Actually, we totally agree with reviewer that image information is critical, and we will focus on solving the above problems in future study.

Modification:

We made the following modification:

- We list the integration of morphological information of spatial transcriptomics data as one of future research directions (Section: 3);

- We also provide the version of MNMST integrated with histology images on the request of users and readers, and clarify the current version for integrating images is not perfect (https://github.com/ xkmaxidian/MNMST/blob/main/tutorials/tutorials_mnmst_histology.ipynb).

*S4: The sequence of discussion in the manuscript does not consistently correspond with the order of subplots in Figures 2 and 3. For example, Figure 2C is referenced before Figure 2B; Figure 3B before Figure 3A, and Figure 3D before Figure 3C. It is strongly recommended to synchronize the narrative with the figures to prevent confusion.*

REPLY: Thanks great for the great suggestion. To address this issue, we first explain why sequence and panels are inconsistent, and then how to overcome it.

Reasons: There are several panels in Figure 2 and Figure 3, and we set layout of figure depends on sizes of panels for aesthetic. In this case, biological stories does not consistently correspond with the order of subplots, resulting in confusion.

Modification: To prevent confusion, we synchronize the narrative with the figures by simultaneously adjusting layout of figures and sequence of discussion. Now, we synchronize the narrative with the figures.

S5: *Supplementary Figures S1-S12 could be consolidated into a single comprehensive figure. Presenting sim­ilar outcomes across twelve separate figures is overly burdensome.*

REPLY: Thank you for the suggestion. Now, we merge S1-S12 into two figures, which dramatically reduce burdensome of this study. Thanks great for the suggestion.

Modification:

We made the following modification:

- The supplementary figures are updated (Supplementary Figure: S1, S2).

S6: *The performance of MNMST appears comparable to that of stLearn as shown in Figure S13. An explanation for this similarity would be beneficial.*

REPLY: Thanks great for the suggestion. We first explain why MNMST and stLearn [19] have a similar performance on Figure S13, and then perform a comparable performance between them.

As shown in Fig. 10 A, MNMST and stLearn are comparable to stLearn on the mouse brain data, demon­strating that these algorithms accurately characterize structure of spatial domains. First, stLearn integrates expression, spatial and morphological information, which provides a comprehensive way to characterize struc­ture of spatial domains. In contrast, MNMST explores expression, spatial and topological structure of cell networks. These results demonstrate that topological structure of cell networks is also promising for spatial domains, which is one of the major motivation of this study.

**A**	MNMST	stLearn	MNMST	stLearn

Figure 10: Comparison of spatial domains identified by MNMST and stLearn. (A) Comparison of identified spatial domains on mouse brain slices. (B) Comparison of identified spatial domains on DLPFC slices.

MNMST ARJ=0.523	stLearn ARJ=0.301	MNMST ARJ=0.588	stLearn ARJ=0.280

**B**

Mouse brain coronal

Mouse brain posterior

DLPFC 151507

DLPFC 151676

However, careful comparison between MNMST and stLearn also shows that the boundary of spatial do­mains identified by stLearn is non-smooth. The reason is that stLearn utilizes morphological information to quantify relative importance of neighbor spots, and then augments expression of spots for spatial domains. In this case, it fails to fully exploit the relations among expression, spatial and morphological information, whereas MNMST jointly learns features of multi-layer networks, thereby improving performance of spatial domain identification as shown Fig. 10.

Modification:

We made the following modification:

- We added the explanation for this similarity between MNMST compared to stLearn (Pages: 8-9, Section: 2.2, Color: red).

S7: *The systematic examination of parameters presented in Figure 3 is great, but certain aspects are not clearly articulated: 7.1 The acronym ’SRL’ used in Figure 3A is not defined as it refer to a self-supervised learning strategy? It should be fully spelled out at least once; 7.2 The comparison presented in Figure 3A might be misleading, contrasting the best Pearson network results with the suboptimal SRL network results, which could confuse readers initially; 7.3 On page 8, line 28, the stated ARI values for Pearson and KNN do not seem to be supported by the corresponding dots in the ARI plot in Figure 3A; 7.4 The term ’cumulative distribution probability’ in Figure 3D requires elucidation.*

REPLY: Thanks great for the suggestion. And, we systematically examine parameters in Figure 3, which are summarized as.

- 7.1: SRL denotes Self-Representation Learning (SRL), which is a popular method for learning similarity graph for spots/cells. Now, we correct as SRL (Self-representation learning).

- 7.2: We replace the optimal solution for SRL to replace the original and suboptimal one in Figure 3A. - 7.3: We mistakenly use variance as standard deviation, and we remove all these typos in this study.

- 7.4 ”Cumulative distribution probability” is also called ”Cumulative Distribution Function (CDF)” such that

*F*(*x*) = *Pr*(*X* ≤ *x*)*,*	(2)

where *X* is a random variable. In Figure 3D, y-axis denotes *F*(*X*) and x-axis represents *X*. We elucidate definition of Cumulative Distribution Function (CDF).

S8: *In the section ”Algorithm analysis” of Supplementary Materials, it is reported that MNMST is compu­tationally intensive, with a time complexity of O* (*n^2^dl*)*, and a memory requirement of O*(*n^2^*) *this could be a limitation for large datasets with high-dimensional data. Based on Supplementary Figures 1-12, the ARIs between DeepST and MNMST are quite similar for 10x Visium datasets (MNMST outperforms for the slice 151670). Even after calculating the Silhouette Coefficient (SC) and Davies-Bouldin (DB) scores for mouse brain posterior and coronal data, the DeepST and MNMST performances are quite similar. Based on the Supplementary Figure 1 of DeepST publication (DeepST: identifying spatial domains in spatial transcrip-tomics by deep learning), DeepST peformed the analysis on the same human dorsolateral prefrontal cortex slices in under 50 minutes and required less 12GB of memory. What is the computational time of MNMST on these slices? Is this comparable to DeepST?*

REPLY: Thanks great for the valuable suggestion and we really appreciate it. To address this issue, we first make a comparative comparison among various algorithms on the running time with different spatial tran-scriptomics datasets, including 10 × Genomics and MERFISH data. And, we then accelerate the proposed algorithm with GPU.

**Space and time of various algorithms**

**Platform:** For a fair comparison, all algorithms are executed on the same platform with Intel i9 CPU, 64GB RAM, and an NVIDIA RTX4090.

On the 10 × Genomics data, we independently execute each algorithm for each slice of DLPFC data [5], where the distributions of running time and space of algorithms are summarized in Fig. 11 A1 and A2, respectively. From Fig. 11 A1, it is easy to conclude that Giotto and BayesSpace are slower than stLearn and MNMST, and SCANPY, SpaGCN and DeepST are faster than others. Specifically, running time of MNMST is 4.3 ± 2.5 minutes, while that is 10.6 ± 2.9 (Giotto), 6.3 ± 2.2 (BayesSpace), 3.6 ± 0.4 (stLearn), 2.2 ± 0.4 (DeepST), 1.4 ± 0.3 (SpaGCN), 0.6 ± 0.1 (SEDR) and 0.1 ± 0.0 (SCANPY), respectively. Fig. 11 A2 illustrates distributions of spaces for various algorithms for DLPFC data, where space complexity of Gitto, stLearn and DeepST much higher than others. In details, space of MNMST is 3.6 ± 0.1 Gigabytes, while that is 5.5 ± 0.4 (Giotto), 1.2 ± 0.1 (BayesSpace), 8.5 ± 1.5 (stLearn), 9.3 ± 0.7 (DeepST), 1.7 ± 0.1 (SpaGCN), 1.9 ± 0.1 (SEDR) and 1.5 ± 0.2 (SCANPY), respectively.

From Fig. 11 A1 and A2, it is easy to assert that

- Gitto and stLearn are time- and space-consuming, hampering the applications of identification of spatial domains in ST data. And, DeepST reduces running time by sacrificing space, whereas BayesSpace reduces space complexity by sacrificing running time.

- SCANPY, SEDR and SpaGCN are efficient in terms of running time and space, whereas performance of these is undesirable. In other words, these algorithms improve efficiency by sacrificing accuracy of algorithms.

- MNMST reaches a good tradeoff between space and running time. Furthermore, it achieves the best performance on the identification of spatial domains, demonstrating that MNMST provides an excellent alternative for current algorithms.

On the MERFISH data [2], we validate efficiency of algorithms by increasing the number of spots/cells from 1,000 to 30,000. Since SEDR , SpaGCN, and DeepST employ graph convolutional network (GCN), and DeepST is superior to them in terms of accuracy. Therefore, only SCANPY, DeepST and MNMST are selected for a comparison. Fig. 11 B1 and B2 describe the running time and space of algorithms with various sizes of data respectively, where missing bar represents failure of algorithms to address the corresponding data. When the number of spots is less than or equal to 20,000, MNMST is worse than DeepST on both space and time. However, MNMST addresses ST data with more than 20,000 spots that DeepST fails to handle. The reason why DeepST fails to address large-scale ST data is that it employs deep learning to obtain features of spots/cells, where the number of parameters for learning exponentially increases, resulting in the expensive time complexity.

**Accelerating MNMST with GPU**

Even though we demonstrate that MNMST reaches a good balance between time and space with the best performance of identifying spatial domains, its efficiency is still undesirable since it fails to address large-scale ST data. To solve this problem, we accelerate MNMST with graphic processing unit (GPU), i.e., accelerating MNMST with hardware. The running time and space of MNMST with CPU and GPU for DPLDC are summarized in Fig. 11 C1 and C2 respectively, where MNMST(GPU) accelerates 100 times by sacrificing extra 20% space, demonstrating that GPU is promising for improving applicability of MNMST. The acceleration strategy is also efficient for MERFISH, where the improvement of time and space is shown in Fig. 11 D1 and D2, respectively.

**Conclusion: MNMST reaches a good balance between time and space, and it achieves the best perfor­mance with the acceptable running time. Furthermore, GPU dramatically accelerates speed of MNMST, which is applicable for large-scale ST data.**

Modification:

We made the following modification:

- A comparative comparison among typical algorithms for efficiency on various spatial transcriptomics data is added (Section: 2.3, Page: 9, Color: Red, Supplementary Figure: S4);

- The GPU version of MNMST software is also provided (website: <https://github.com/xkmaxidian/> MNMST/blob/main/MNMST_gpu.py).

S0: *In the Method section ”Identification and functional analysis of differentially expressed genes” it is re­ported that differential expression analysis of genes for each spatial domain was performed used Wilcoxon rank-sum test implemented in SCANPY. The Wilcoxon rank-sum test should not be used for differential gene expression analysis because it does not account for within-sample correlation as reported in the review by Heumos, Schaar et at. (Best practices for single-cell analysis across modalities). I would suggest to use SpatialDE as reported in the Single-cell best practices web page* ***( https: // www. sc-best-practices. org/ spatial/ spatially_ variable_ genes. html)****. In addition, for the gene ontology enrichment analysis clus-terProfiler was used. Was this analysis performed using the enrichGO function? If the enrichGO function was used, why the authors plotted the -log10(pval) instead of the pAdjust provided by the tool? When perform­ing multiple tests, it is better to use the adjusted p-value because it incorporates multiple testing correction.*

REPLY: Thanks great for the suggestion. Actually, we perform differential expression genes with spatialDE [20], and find that spatialDE identifies 661 DEGs among 672 DEGs. Therefore, we add p-values and q-values of genes with both strategies. On the enrichment issue, we already correct the typos.

S1: *Improved documentation on the GitHub page with clear guidance on how to select optimal parameters for different spatial transcriptomics methodologies would be beneficial for users. The provided tutorial only shows the analysis for the 10x Visium dataset.*

REPLY: Thank you for the comment. We update tutorials about different spatial technologies, including imaging-based STARmap, osmFISH, and sub-cell resolution Stereo-seq, which can be accessed at https: //[github.com/xkmaxidian/MNMST/tree/main/tutorials.](http://github.com/xkmaxidian/MNMST/tree/main/tutorials.)

Minor point:

*1 Line 2 = ”many cell types (sub-populations) locating” should be changed with “many cell types (sub-populations) located”.*

*2 Line 37 = ”domains are roughly categories into two classes” should be changed with “domains are roughly categorized into two classes”.*

REPLY: Thank you for your comments. We proofread the whole manuscript and correct typos by turning to native speakers. Furthermore, we also employ language edit service to address this issue. There are too many to list them all.

**References**

[1] Zeira R, Land M, Strzalkowski A, Raphael BJ. Alignment and integration of spatial transcriptomics data. Nat Methods. 2022;19(5):567–575.
[2] Moffitt JR, Bambah-Mukku D, Eichhorn SW, Vaughn E, Shekhar K, Perez JD, et al. Molec-

ular, spatial, and functional single-cell profiling of the hypothalamic preoptic region.	Science.
2018;362(6416):eaau5324.

[3] Xu C, Jin X, Wei S, Wang P, Luo M, Xu Z, et al. DeepST: identifying spatial domains in spatial transcriptomics by deep learning. Nucleic Acids Res. 2022;50:e131–e131.
[4] Long Y, Ang KS, Li M, Chong KLK, Sethi R, Zhong C, et al. Spatially informed clustering, integration, and deconvolution of spatial transcriptomics with GraphST. Nat Commun. 2023;14(1):1155.
[5] Maynard KR, Collado-Torres L, Weber LM, Uytingco C, Barry BK, Williams SR, et al. Transcriptome-scale spatial gene expression in the human dorsolateral prefrontal cortex. Nat Neurosci. 2021;24:425–436.
[6] Wolf FA, Angerer P, Theis FJ. SCANPY: large-scale single-cell gene expression data analysis. Genome Biol. 2018;19:1–5.
[7] Hu J, Li X, Coleman K, Schroeder A, Ma N, Irwin DJ, et al. SpaGCN: Integrating gene expression, spa­tial location and histology to identify spatial domains and spatially variable genes by graph convolutional network. Nat Methods. 2021;18:1342–1351.
[8] Dong K, Zhang S. Deciphering spatial domains from spatially resolved transcriptomics with an adaptive graph attention auto-encoder. Nat Commun. 2022;13:1–12.
[9] Palla G, Spitzer H, Klein M, Fischer D, Schaar AC, Kuemmerle LB, et al. Squidpy: a scalable framework for spatial omics analysis. Nat Methods. 2022;19:171–178.
[10] St˚ahl PL, Salmén F, Vickovic S, Lundmark A, Navarro JF, Magnusson J, et al. Visualization and analysis of gene expression in tissue sections by spatial transcriptomics. Science. 2016;353:78–82.
[11] Wei X, Fu S, Li H, Liu Y, Wang S, Feng W, et al. Single-cell Stereo-seq reveals induced progenitor cells involved in axolotl brain regeneration. Science. 2022;377:eabp9444.
[12] Stickels RR, Murray E, Kumar P, Li J, Marshall JL, Di Bella DJ, et al. Highly sensitive spatial transcriptomics at near-cellular resolution with Slide-seqV2. Nat Biotechnol. 2021;39:313–319.
[13] Wang X, Allen WE, Wright MA, Sylwestrak EL, Samusik N, Vesuna S, et al. Three-dimensional intact-tissue sequencing of single-cell transcriptional states. Science. 2018;361:eaat5691.
[14] Codeluppi S, Borm LE, Zeisel A, La Manno G, van Lunteren JA, Svensson CI, et al. Spatial organization of the somatosensory cortex revealed by osmFISH. Nat Methods. 2018;15:932–935.
[15] Lakkaraju H, Bach SH, Leskovec J. Interpretable decision sets: A joint framework for description and prediction. In: Proc. ACM SIGKDD; 2016. p. 1675–1684.
[16] Liu W, Liao X, Yang Y, Lin H, Yeong J, Zhou X, et al. Joint dimension reduction and clustering analysis of single-cell RNA-seq and spatial transcriptomics data. Nucleic Acids Res. 2022;50(12):e72–e72.
[17] Wu W, Ma X. Joint learning dimension reduction and clustering of single-cell RNA-sequencing data. Bioinformatics. 2020;36:3825–3832.
[18] Bao F, Deng Y, Wan S, Shen SQ, Wang B, Dai Q, et al. Integrative spatial analysis of cell morphologies and transcriptional states with MUSE. Nat Biotechnol. 2022;40(8):1200–1209.

[19]	Pham D, Tan X, Xu J, Grice LF, Lam PY, Raghubar A, et al. stLearn: integrating spatial location, tissue morphology and gene expression to find cell types, cell-cell interactions and spatial trajectories within undissociated tissues. bioRxiv. 2020;p. 2020–05.

[20]	Svensson V, Teichmann SA, Stegle O. SpatialDE: identification of spatially variable genes. Nature methods. 2018;15(5):343–346.

**Second round of review**

**Reviewer 1**

The authors have put considerable effort into the revision and have addressed all my comments. However, the changes made to the manuscript raise some additional points that should be addressed before publication.

major comments:
- The authors now provide a Python-only version of their method as a PyPI package, which is a big improvement over the first version. The addition of a GPU version is also highly appreciated. However, the package is very rudimentary: For one, it lacks dependency information, such that installing it in a clean virtual environment will not install any of its dependencies and trying to use the package will rase an ImportError. Packages which mnmstpy depends on should be listed in the appropriate section in pyproject.toml. Furthermore, it is not clear how the PyPI package is built from the Github repository: The package containes multiple files that are not in the Github repository (e.g. __init__.py, pyproject.toml), while the Github repository contains many files and directories that are not in the PyPI package. The package version in pyproject.toml also does not correspond to the version one gets from mnmnst.__version__. I strongly encourage the authors to familiarize themselves with current best practices for Python package development.

minor comments:
- Fig. 2E: Some of the distributions are decidedly non-Gaussian, so a t-test is not appropriate. A non-parametric test such as the Mann-Whitney U test would be more appropriate.
- Fig. S4: It is unclear if the plotted memory for the GPU version refers to host (CPU) or device (GPU) memory. Both are important and should be shown.
- Section 2.3: The authors claim that "MNMST [...] reaches a good tradeoff between space and running time". According to Fig. S4B, that claim is only true for data sets smaller than 10k spots, on larger datasets MNMST is both slower and requires more memory than its competitors.
- Fig. 3B and the corresponding main text: The DeLong test was specifically derived for comparing ROC curves, where one ROC curve yields one Mann-Whitney statistic calculated from the predicted positive and predicted negative samples. It is thus not clear to me how the DeLong test is used to compare two distributions, which only yield one Mann-Whitney statistic in total. In general, the Kolmogorov-Smirnov test is a non-parametric test for comparing probability distributions.
- Captions for Fig. 3 C and D are switched
- Captions for Fig. 3A, C, D still do not explain the boxplots. There is an explanation in the caption for panel F, but it states that whiskers show the min-max range, wihle in panel C there is a blue datapoint outside of the whiskers range.
- Fig S7D: What are the data and what is the ground truth/ARI?
- Page 14 bottom: How is SpatialDE used to select differentially expressed genes between domains? SpatialDE can detect spatially variable genes, but it cannot perform differential gene expression testing between two sets of spots.
- Supplementary section 1.2, first line: I think you intended to reference equation (4).
- Fig. S5: 3D plots in general should be avoided. For these plots in particular, parts of the plots are obscured. A normal heatmap (gamma and lambda on the axes, ARI in color) would be more useful.
- MNMST without joint learning: The explanation given in the reply to my comment S9 ("we independently execute...") really should be in the methods section.
- Batch correction: I'm sorry, I still don't understand what "It constructs cell networks from multiple batches by concatenating these in each batch" means.

**Reviewer 2**

The authors have addressed my comments and concerns.

**Authors Response**

**Point-by-point responses to the reviewers’ comments:**

**Reviewer # 1**

*The authors have put considerable effort into the revision and have addressed all my comments. However, the changes made to the manuscript raise some additional points that should be addressed before publication.*

**REPLY:** Thanks great for your valuable comments. We have addressed all these suggestions one by one. On the minor issues, some suggestions that are highly related are addressed together.

**Major Problems**

***S1****: The authors now provide a Python-only version of their method as a PyPI package, which is a big improvement over the first version. The addition of a GPU version is also highly appreciated. However, the package is very rudimentary: For one, it lacks dependency information, such that installing it in a clean virtual environment will not install any of its dependencies and trying to use the package will raise an Im-portError. Packages which mnmstpy depends on should be listed in the appropriate section in pyproject.toml. Furthermore, it is not clear how the PyPI package is built from the Github repository: The package con-*

*tains multiple files that are not in the Github repository (e.g.	init .py, pyproject.toml), while the Github
repository contains many files and directories that are not in the PyPI package. The package version in pyproject.toml also does not correspond to the version one gets from mnmnst. version . I strongly encour­age the authors to familiarize themselves with current best practices for Python package development.*

**REPLY:** Thanks great for the valuable suggestion and we really appreciate it. To address this issue, we refer to the best practices for Python package development [1] to improve MNMST package.

In details, we first add dependencies for MNMST packages, which is included in pyproject.toml. Then, we give the tutorial for installing MNMST package from github, and installation is tested on various platforms. Finally, we make the consistence between Github and PyPI packages.

**Dependencies of MNMST**

MNMST makes use of 12 python packages, and the dependencies are summarized as

- scanpy >= 1.9.3 (<https://scanpy.readthedocs.io/en/stable/installation.html)>

- numpy>=1.24.0 (<https://numpy.org/install/)>

- pandas>=1.5.0 (<https://pandas.pydata.org/getting> started.html)

- anndata>=0.9.0 (<https://github.com/scverse/anndata)>

- matplotlib>=3.7.1 (<https://matplotlib.org/stable/users/installing/index.html)>

- scipy>=1.11.2 (<https://scipy.org/install/)>

- scikit-learn>=1.2.2 (<https://scikit-learn.org/stable/install.html)>

- psutil>=5.9.0 (<https://psutil.readthedocs.io/en/latest/)>

- tqdm>=4.65.0 (<https://tqdm.github.io/)>

- leidenalg>=0.10.1 (<https://leidenalg.readthedocs.io/en/stable/install.html)>

- torch>=2.0.1 (<https://pytorch.org/get-started/locally/)>

3

- torchvision*>*=0.15.2 (<https://github.com/pytorch/vision)>

And, all these dependencies are included in the pyproject.toml, where versions of dependencies are spec­ified (<https://github.com/xkmaxidian/MNMST/blob/main/mnmstpy-package/pyproject.toml).>

**Installation and test of MNMST**

We provide multiple way to install MNMST, i.e., conda and Github. In details,

1. **Installation using python virtual environment with conda:**

*>> conda create –name mnmst python=3.9*

*>> conda activate mnmst*

*>> pip install mnmstpy*

(Optional) To run the notebook files in tutorials, please ensure the Jupyter package is installed in your

environment:

*>> conda install -n mnmst ipykernel*

*>> python -m ipykernel install –user –name mnmst –display-name mnmst-jupyter*

2. **Installation from Github**

*>> git clone* [*https://github.com/xkmaxidian/MNMST*](https://github.com/xkmaxidian/MNMST)

*>> cd <your dir path>/MNMST/MNMST-package*

*>> python* [*setup.py*](http://setup.py) *build*

*>> python* [*setup.py*](http://setup.py) *install*

The tutorial for installation is available at (<https://github.com/xkmaxidian/MNMST/blob/main/README.md).>

To ensure execution of MNMST, we testify it on four various platforms, which are list as

- **Platform 1** Operation System (OS): Linux 5.4.0, Python: 3.10.12

- **Platform 2** Operation System (OS): Google Colab (Linux 6.1.58), Python: 3.10.12

- **Platform 3** Operation System (OS): Windows 10, Python: 3.10.9

- **Platform 4** Operation System (OS): Windows 11, Python: 3.9.18

MNMST is executable on all these four typical platforms. and test for the Google Colab platform is available at (<https://colab.research.google.com/drive/19c6g02WCcj9uJlUCuyIkPXkwrnJACeEW?usp=sharing).>

**Consistence between Github and PyPI**

We also ensure consistence between Github and PyPI, including the version information, and so on.

**Minor Problems**

***S1****: Fig. 2E: Some of the distributions are decidedly non-Gaussian, so the t-test is not appropriate. A non-parametric test such as the Mann-Whitney U test would be more appropriate*

**REPLY:** Thank you for the suggestion. We replace the t-test with the Mann-Whitney U test to re-calculate p-value. The difference is even more significant. And, we make the following modification:

- We correct test for Fig. 2E with Mann-Whitney U test (Page: 7, Figure: 2, Color: red); - We correct description of Fig. 2E (Page: 8, Color: red).

***S2****+****S3: Issues for Fig. S4****:*

*- Section 2.3: The authors claim that ”MNMST [...] reaches a good tradeoff between space and running time”. According to Fig. S4B, that claim is only true for data sets smaller than 10k spots, on larger datasets MNMST is both slower and requires more memory than its competitors.*

*- Fig. S4: It is unclear if the plotted memory for the GPU version refers to host (CPU) or device (GPU) memory. Both are important and should be shown.*

REPLY: Thanks great for the suggestion. To address these issues, we first explain why we make such an assertion and memory, and then propose the modification to remove confusion.

Explanation

On the assertion issue: we claim that MNMST reaches a good tradeoff between space and running time according to Fig. 1 A. In details, Fig. 1 A1 shows that MNMST is faster than Giotto and BayesSpace, but slower than others on running time. Fig. 1 A2 demonstrates that MNMST is beter than Giotto, stLearn, and DeepST, but worse than others on space.

The original motivation for last version of Fig. S4B is to show that MNMST is still worse than SCANPY and DeepST in terms of space and time complexity, which is the reason why we develop GPU version of MNMST. But, we ignore that it is very likely to cause confusion. Thanks great for your valuable suggestion.

On the memory issue: the reported memory usage for the CPU refers to the host (CPU) memory, while the reported memory for the GPU represents the combination of host (CPU) and device (GPU) memory. It also leads to confusion for readers.

Modification

To remove confusion, we re-draw the figure as Fig. 1, which are summarized as

- To remove confusion on assertion, we move comparison among MNMST, SCANPY and DeepST into Fig. 1 C.

- To remove confusion on memory, we specify the host (CPU) and device (GPU) memory for each algorithms with different colors as Fig. 1.

***S4****+****S5: Issues for Fig. 3****:*

*- Fig. 3B and the corresponding main text: The DeLong test was specifically derived for comparing ROC curves, where one ROC curve yields one Mann-Whitney statistic calculated from the predicted positive and predicted negative samples. It is thus not clear to me how the DeLong test is used to compare two distributions, which only yield one Mann-Whitney statistic in total. In general, the Kolmogorov-Smirnov test is a non-parametric test for comparing probability distributions.*

*- (1) Captions for Fig. 3C and D are switched. (2) Captions for Fig. 3A, C, D still do not explain the boxplots. There is an explanation in the caption for panel F, but it states that whiskers show the min-max range, while in panel C there is a blue data point outside of the whiskers range.*

REPLY: Thanks great for the suggestion and we really appreciate. The Figure is updated as Fig. 2

- The significance of probability distribution of Fig. 2B is obtained by Kolmogorov-Smirnov test (p-value is also less than 2.2E-16).

- We correct the captions for Fig. 2C and D.

- We explain the specific meaning of each component in the pirateplots in Fig. 3A and D, and the boxplots in Fig. 3C and F.

Figure 3: Performance of MNMST and DeepST for mouse brain slice S3 in terms of ART, where the manual annotation of slide S3 as ground truth.

S7: *Fig. S7D: What are the data and what is the ground truth/ARI?*

REPLY:Thank you for the comment. The data in Fig. S7D consists of two mouse breast cancer slices acquired with 10× Visium, where slice S1 consists of 1,868spots, and slice S3 consists of 1950 spots from Zendo (<https://zenodo.org/records/10369465)> [2]. Ref.[2] only reports the manual annotations of slide S3. We only report ART of slide S3 for various algorithms as shown in Fig. 3, where MNMST is much better than DeepST (ART: 0.501 vs 0.261). Furthermore, these spatial domains identified by MNMST in slide S1 and S3 are consistent, whereas these differ greatly.

S8: *Page 14 bottom: How is SpatialDE used to select differentially expressed genes between domains? Spa-tialDE can detect spatially variable genes, but it cannot perform differential gene expression testing between two sets of spots.*

REPLY: Thanks great for the comment. Tt is our problem for not explaining it clearly. Actually, the differentially expressed genes (DEGs) are selected by using the Wilcoxon rank-sum test and fold change (Section 5.7) without SpatialDE. We adopt SpatialDE to remove these DEGs that are not highly variable. And,we find that 661 out of 672 DEGs for domain 0, 1, and 2 are highly variable genes. The downstream analysis are based on these 661 DEGs.

And, we correct the procedure for DEGs in Section 5.7, and remove unnecessary description.

*S9 Supplementary section 1.2, first line: I think you intended to reference equation (4).*

REPLY: Thanks great for pointing out this critical typo, and we already correct it.

S10: *Fig. S5: 3D plots in general should be avoided. For these plots in particular, parts of the plots are obscured. A normal heatmap (gamma and lambda on the axes, ARI in color) would be more useful.*

REPLY: Thank you for the suggestion, and we replace the original 3D plots with heatmaps as shown in Fig. 4.

S11:*MNMST without joint learning: The explanation given in the reply to my comment S9 (”we indepen­dently execute...”) really should be in the methods section.*

**REPLY:** Thank you for the comment, we add the explanation of MNMST without joint learning in main

text (Section: 5.3, Page: 21, Color: red).

***S12****: Batch correction: I’m sorry, I still don’t understand what ”It constructs cell networks from multiple*

*batches by concatenating these in each batch” means.*

**REPLY**: Thank you for the comment. It is our problem for not explaining it clearly. Specifically, MNMST

first stacks multiple slices from various batches into one slice with SCANPY [3], and then constructs cell

spatial network and cell expression network for the integrated slice, where cross-slice spatial proximity of

spots enhances consistence of features of adjacent spots, thereby removing batch effect at some extent.

**Modification:**

We made the following modifications:

- We correct the description (Section: 2.4, Page: 13, Color: red).

**References**

[1] Virshup I, Bredikhin D, Heumos L, Palla G, Sturm G, Gayoso A, et al. The scverse project provides a computational ecosystem for single-cell omics data analysis. Nature biotechnology. 2023:1-3.
[2] Long Y, Ang KS, Li M, Chong KLK, Sethi R, Zhong C, et al. Spatially informed clustering, integration, and deconvolution of spatial transcriptomics with GraphST. Nat Commun. 2023;14(1):1155.
[3] Wolf FA, Angerer P, Theis FJ. SCANPY: large-scale single-cell gene expression data analysis. Genome Biol. 2018;19:1-5.

**Third round of review**

**Reviewer 1**

The authors have addressed all my comments. There are two minor issues with Fig. S4C2:
- The memory requirements for the GPU version of MNMST still do not differentiate between host and GPU memory
- In the response letter, the authors state that memory of the MNMST GPU version was calculated as the aggregate of host and GPU memory. In this case, the MNMST GPU memory should be larger than the MNMST CPU memory, but it is smaller. The authors should double-check if they did not mix up the CPU and GPU versions.

These should be addressed before publication.

**Authors Response**

**Point-by-point responses to the reviewers’ comments:**

**Reviewer # 1**

*The authors have addressed all my comments. There are two minor issues with Fig. S4C2:*

*- The memory requirements for the GPU version of MNMST still do not differentiate between host and GPU memory.*

*- In the response letter, the authors state that memory of the MNMST GPU version was calculated as the aggregate of host and GPU memory. In this case, the MNMST GPU memory should be larger than the MNMST CPU memory, but it is smaller. The authors should double-check if they did not mix up the CPU and GPU versions.*

REPLY: Thanks great for the valuable suggestion. We find out that the inconsistence is due to the precision of Numpy (float64) and PyTorch (float32) in Fig. S4C2. Now, we update differentiation of host and GPU memory, as well as correct MNMST GPU and CPU, as shown in Fig. 1. Furthermore, the corresponding memory usage of MNMST-CPU is also updated.
